# Catastrophic health expenditure, incidence, trend and socioeconomic risk factors in China: A systematic review and meta-analysis

**DOI:** 10.3389/fpubh.2022.997694

**Published:** 2023-01-04

**Authors:** Fangkai Zhang, Jianjun Jiang, Min Yang, Kun Zou, Dandi Chen

**Affiliations:** ^1^West China School of Public Health, West China Fourth Hospital, Sichuan University, Chengdu, Sichuan, China; ^2^Research Center for Palliative Care, West China-PUMC C.C. Chen Institute of Health, Sichuan University, Chengdu, Sichuan, China; ^3^West China Research Centre for Rural Health Development, Sichuan University, Chengdu, Sichuan, China; ^4^Faculty of Health, Art and Design, Swinbune Technology University, Melbourne, VIC, Australia; ^5^Department of Pharmacy, West China Second University Hospital, Sichuan University, Chengdu, China; ^6^Evidence-Based Pharmacy Center, West China Second University Hospital, Sichuan University, Chengdu, China; ^7^NMPA Key Laboratory for Technical Research on Drug Products In Vitro and In Vivo Correlation, Chengdu, China; ^8^Key Laboratory of Birth Defects and Related Diseases of Women and Children, Sichuan University, Ministry of Education, Chengdu, China

**Keywords:** influencing factors, systematic review, meta-analysis, catastrophic health expenditure, social-economic risk factors

## Abstract

**Objective:**

To evaluate the incidence and trend of catastrophic health expenditures (CHE) in China over the past 20 years and explore the socioeconomic factors affecting China's CHE rate.

**Methods:**

The systematic review was conducted according to the Cochrane Handbook and reported according to PRISMA. We searched English and Chinese literature databases, including PubMed, EMbase, Web of Science, China National Knowledge Infrastructure (CNKI), Wan Fang, China Science and Technology Journal Database (CQVIP), and CBM (Sino Med), for empirical studies on the CHE rate in China and its associated socioeconomic factors from January 2000 to June 2020. Two reviewers conducted the study selection, data extraction, and quality appraisal. The secular trend of the CHE rate was examined, and factors associated with CHE were explored using subgroup analysis and meta-regression.

**Results:**

A total of 118 eligible studies with 1,771,726 participants were included. From 2000 to 2020, the overall CHE rate was 25.2% (95% CI: 23.4%−26.9%) in China. The CHE rate continued to rise from 13.0% in 2000 to 32.2% in 2020 in the general population. The CHE rate was higher in urban areas than in rural areas, higher in the western than the northeast, eastern, and central region, in the elderly than non-elderly, in low-income groups than non-low-income groups, in people with cancer, chronic infectious disease, and cardio-cerebrovascular diseases (CCVD) than those with non-chronic disease group, and in people with NCMS than those with URBMI and UEBMI. Multiple meta-regression analyses found that low-income, cancer, CCVD, unspecified medical insurance type, definition 1 and definition 2 were correlated with the CHE rate, while other factors were all non-significantly correlated.

**Conclusion:**

In the past two decades, the CHE rate in China has been rising. The continuous rise of health expenditures may be an important reason for the increasing CHE rate. Age, income level, and health status affect the CHE rate. Therefore, it is necessary to find ways to meet the medical needs of residents and, at the same time, control the unreasonable rapid increase in health expenditures in China.

## Introduction

Catastrophic health expenditure (CHE) is a global problem affecting universal health coverage and poverty reduction, and CHE is likely to occur in countries with different levels of economic development (1). CHE is defined as out-of-pocket health expenditures as a proportion of household consumption that exceeds a certain standard (2). The World Health Organization recommends that the standard for defining CHE is that household mandatory health expenditure accounts for more than 40% of non-food consumption expenditure (3). According to this standard, in 2010, 208 million people worldwide suffered CHE, and 97 million people became impoverished due to out-of-pocket (OOP) health expenditures, which is equivalent to 1.4% of the global population (4). In 2010, 2012, 2014, and 2016, the CHE rates in China were 13.58, 11.95, 11.43, and 11.06%, respectively, a relatively high level globally (5).

From 2000 to 2020, China's medical system underwent tremendous changes. In 2000, China started a series of medical system reforms, starting with the reform of the urban health system, which was the beginning of the medical system reform over the past two decades. In 2009, China began to deepen the reform of the medical system and proposed the establishment of a basic medical system and a security system to cover the basic medical and health care of urban and rural residents by 2020 (6). During the two decades of reform of the medical and health system, China has made great efforts to reduce the medical burden on residents. The initial establishment of the national essential drug system and the abolition of drug markups have played a positive role in reducing the medical burden on residents (7, 8). A major achievement was the establishment of the medical insurance system composed of basic medical insurance, critical illness insurance (CII), and medical assistance. By 2020, 1.361 billion people (remained stable at over 95%) in China were covered by basic medical insurance, including basic medical insurance for employees and basic medical insurance for urban and rural residents. In addition, China launched a health poverty alleviation program to address the problem of health expenditures for low-income people and complete poverty alleviation by 2020 (9).

However, with the development of the economy and the increase in residents' income, residents' health services needs were released. The requirements for the quality of health services have also increased correspondingly, and the total health expenditure (THE) and total OOP health expenditure have continued to rise (5). China's THE rose from 1,998.04 billion in 2010 to 7,230.64 billion in 2020, and the total OOP health expenditure rose from 705.29 billion in 2010 to 2,005.53 billion in 2020. In addition, China entered an aging society in 2000. In recent years, the degree of aging has further intensified. In 2020, China had 191 million people aged 65 or above, accounting for 13.5% of the total population. The burden of non-communicable diseases brought about by the aging of the population and changes in the disease spectrum have presented a severe challenge to China's health care system and placed a heavy burden on residents' health expenditures (10, 11). Previous studies have shown that Chinese residents have a heavy burden of OOP medical expenditures. Due to improvements in basic health insurance, the proportion of Chinese residents' total OOP health expenditure in THEs fell from 59.5% in 2000 to 27.7% in 2020. However, since 2017, the decline in the proportion of total OOP health expenditures to total health expenditures has significantly slowed (12). Worse still, the CHE had a trend of expanding from low-income to median-income families (13).

Previous studies mainly used public data to assess the trend of CHE, including China Family Panel Studies (CFPS) and the National Health Service Survey. However, due to the timeliness of data disclosure, studies using these two types of data ended in 2020 and 2008, respectively. Because of the different survey methods, the conclusions drawn by various public data studies were also different. A large number of studies have also explored the factors that affect CHE in China. However, most of these studies had a small sample size and focused on a single factor, which affected the extrapolation of conclusions. In addition, different studies adopted different definitions of CHE, which led to poor comparability.

Considering the limitations of the literature, we systematically reviewed the CHE trend and its associated factors in China over the past two decades. Moreover, we carried out a meta-analysis to quantify the magnitude of the outcomes. We included as many influencing factors related to the incidence of CHE as possible in the study to obtain a comprehensive result. We also summarized the effect of the relevant measures of China's medical system reform in reducing CHE and provided suggestions for further reforms in the future. In addition, we grouped the literature according to different definitions of CHE and analyzed the impact of different definitions on the incidence of CHE.

## Methods

### Inclusion and exclusion criteria

We included cohort studies, case-control studies and cross-sectional studies. All the participants were from mainland China. We extracted data on age, income, region, disease and medical insurance. All included studies reported the CHE rate and the number of people with and without CHE. Languages were restricted to English and Chinese, as they were the main publication languages of studies from mainland China. We excluded duplicate publications and excluded informal publications, such as conference papers and reviews.

### Literature search

We systematically searched the Chinese literature databases of the China National Knowledge Infrastructure (CNKI), China Science and Technology Journal Database (CQVIP), and English literature databases, including PubMed, EMbase, and Web of Science, from January 2000 to June 2020. The last retrieval date was June 30, 2021. We used the keywords to search. The search strategy was (Catastrophic health expenditure OR Catastrophic medical expenses OR Poverty-causing health expenditure OR Poverty due to illness OR Return to poverty due to illness) AND China (Appendix Table 1 in Supplementary material). In addition, reference lists of included studies were scanned for more eligible studies.

### Study selection

One reviewer (YXW) conducted the study selection and checked it with another (FRL or QQY). First, the title and abstract of citations were scanned, and irrelevant studies were excluded. Then, the full texts of potentially eligible studies were read and selected according to the inclusion and exclusion criteria. Different opinions were decided through discussion or consultation with a senior reviewer (KZ or DDC).

### Data extraction

We used a standardized Excel form to extract data from the included studies, including the study author, publication year, region, study period, participants, sample size, income level, types of health insurance, definition of CHE, CHE rate, and odds ratio (OR). One reviewer (YXW) evaluated the quality of the included studies using the AHRQ scale and the NOS scale, which was checked by a second reviewer (FRL or QQY). Finally, the reviewer (FKZ) reviewed the data and further extracted the required data, including age, disease type, and residential area (urban or rural area). Different opinions of researchers were resolved by discussion. If multiple reports used the same data source, the most comprehensive publication was used in the analysis.

### Quality assessment

Two reviewers independently rated the risk of bias in the cohort and case-control studies using the NOS (Newcastle–Ottawa Scale) and cross-sectional studies using the AHRQ (Agency for Health Care Research and Quality). Disagreements were resolved by consensus. We contacted authors when information was not reported in the article.

### Certainty of evidence

Two reviewers independently rated the certainty of each study on the GRADE (Grading of Recommendations, Assessment, Development and Evaluation) method.

### Statistical analysis

The pooled CHE rate and 95% confidence interval (CI) were estimated using meta-analysis. The χ^2^ test was used to judge the heterogeneity between the studies. *p* < 0.1 was considered statistically significant. If there was no significant heterogeneity between the studies (*I*^2^ < 50%), the fixed effects model was used; otherwise, the random-effects model was used.

First, the trend of CHE in China in the past two decades was estimated. All included studies and participants were divided into five subgroups, namely, 2000–2008, 2009–2012, 2013–2014, 2015–2016, and 2017–2020, considering the time points of important health policies relevant to CHE. For example, China started a new medical reform in 2009 and established a critical illness protection mechanism in 2012. In 2016, China initially established a medical insurance system consisting of basic medical insurance, CII and medical assistance and abolished drug markups in 2017. These were findings that were effective in curbing OOP health expenditure (14, 15).

In addition, to evaluate the impact of age, health status, and income on secular trends in CHE, we collated studies not specific to elderly, low-income, and disease-specific populations and classified these subjects as the general population. According to the grouping rules mentioned above, the same analysis method was used again.

Second, subgroup analysis was used to explore factors affecting CHE. To examine the differences between CHE in urban and rural areas, the included studies were divided into three groups: the rural group, the urban group, and the unspecified group. According to the classification standards of the National Bureau of Statistics of China in 2011, China could be divided into four major economic regions, namely, the eastern region, the central region, the western region and the northeast region (16). China's economic level was characterized by a prominent “stepping” feature, which shows that the eastern region > the central region > the western region. From the perspective of the economic development level, low- and medium-level areas occupied the dominant position, mainly concentrated in the central and western regions; medium-high and high-level regions were concentrated in the eastern coastal regions, and the central and western regions were scattered (17). Based on this feature, the included studies were divided into five groups: the western, central, eastern, and northeast regions, and the unspecified group.

To explore the impact of age on CHE, the primary studies were divided into three age groups, namely, the elderly group (age ≥ 65), the non-elderly group (age < 65), and the unspecified group. To explore the impact of income on CHE, the studies included were divided into three groups: low-income, non-low-income, and unspecified. By the Order of the State Council of the People's Republic of China (No. 271), we defined the low-income level as “income lower than the poverty line or minimum living standard level set by the local civil affairs department.” To explore the impact of different diseases on CHE, participants were categorized into five subgroups, including the cancer group, cardio-cerebrovascular diseases (CCVD) group, diabetes group, chronic infectious disease group (including tuberculosis, AIDS, viral hepatitis, schistosomiasis), and unspecified chronic disease group (the number of CHEs caused by each chronic disease in the studies was not clearly given), non-chronic disease group (the cause of CHE in the studies was not due to chronic diseases but was caused by accidental injuries, childbirth, etc.) and the unspecified group (the studies did not specify what disease it was). To explore the impact of different medical insurances on CHE, the studies included were divided into five subgroups: NCMS, UEBMI, URBMI, CMI and unspecified group. To explore the impact of the definition of CHE on the CHE rate, the included studies were divided into four subgroups, namely, definition 1 (family out-of-pocket health expenditure exceeding 40% of household income or expenditure within a certain period), definition 2 (family out-of-pocket expenditure on medical and health exceeding 40% of household non-food expenditure), definition 3 (the ratio of household medical and health expenditure to household consumption expenditure exceeds 40%) and definition 4 (others).

A multiple meta-regression analysis was performed to explore the relationship between the risk factors (urban-rural differences, level of socioeconomic status, age differences, income differences, disease differences, medical insurance and definition of CHE) and the CHE rate. A total of 118 studies entered the meta-regression. We used the theoretically lowest CHE group as the control group in the meta-regression.

A funnel plot was used to assess publication bias qualitatively with a visual inspection. Egger's test and trim and fill analysis were used to quantitatively evaluate publication bias. All analyses were performed using STATA 16.0.

## Results

### Characteristics of the included studies

**Figure 1:**
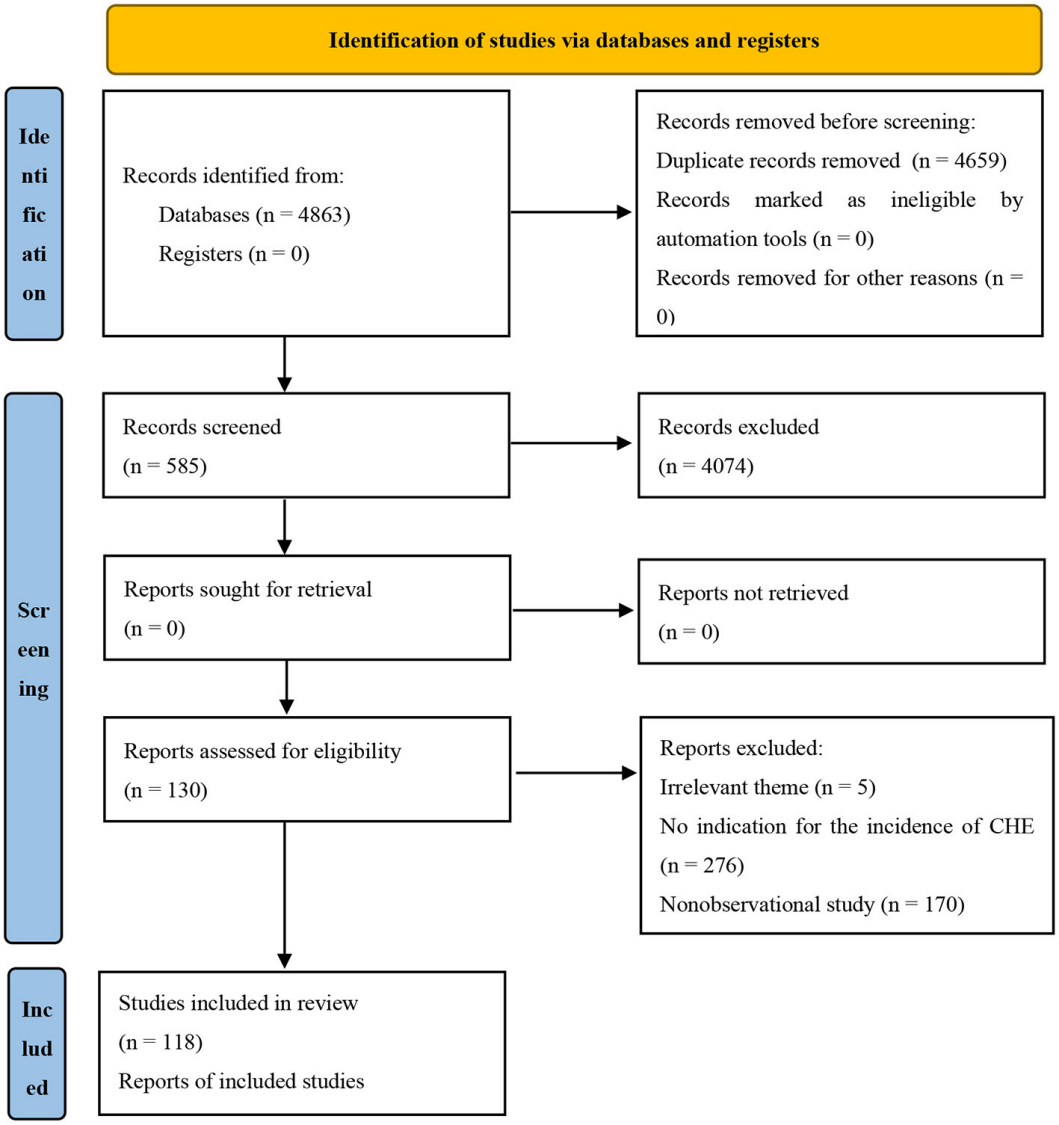
Flow chart of study selection.

In total, 4,863 citations were obtained through a systematic literature search, among which 585 citations were included after the preliminary screening of titles and abstracts. Finally, after reading the full texts, 118 studies with 1,771,726 participants were included (Figure 1).

The publication year was from 2000 to 2020. Among the 118 studies, 81 were cross-sectional, 32 were case controls, and 5 were cohort studies (Table 1).

**Table 1:** Summary of characteristics of included studies [*n*, (%)].

**Study characteristics**		**Research type**			
		**Cross sectional**	**Case control**	**Cohort**	**Total**
Definition of CHE	Definition 1	17	12	1	30
		(14.41%)	(10.17%)	(0.85%)	(25.42%)
	Definition 2	37	15	4	56
		(31.36%)	(12.71%)	(3.39%)	(47.46%)
	Definition 3	12	5	0	17
		(10.17%)	(4.24%)	(0.00%)	(14.41%)
	Others	15	0	0	15
		(12.71%)	(0.00%)	(0.00%)	(12.71%)
	Total	81	32	5	118
		(68.64%)	(27.12%)	(4.24%)	(100.00%)
Research period	2008 and before	4	6	4	14
		(3.25%)	(4.88%)	(3.25%)	(11.38%)
	2009–2012	13	5	4	22
		(10.57%)	(4.07%)	(3.25%)	(17.89%)
	2013–2014	17	6	2	25
		(13.82%)	(4.88%)	(1.63%)	(20.33%)
	2015–2016	21	8	0	29
		(17.07%)	(6.50%)	(0.00%)	(23.58%)
	2017–2020	26	7	0	33
		(21.14%)	(5.69%)	(0.00%)	(26.83%)
	Total	81	32	7	123
		(65.85%)	(26.02%)	(5.69%)	(100.00%)
Urban-rural	Rural	45	18	10	73
		(35.16%)	(14.06%)	(7.81%)	(57.03%)
	Urban	7	4	4	15
		(5.47%)	(3.13%)	(3.13%)	(11.72%)
	Unspecified	29	11	0	40
		(22.66%)	(8.59%)	(0.00%)	(31.25%)
	Total	86	35	7	128
		(67.19%)	(27.34%)	(5.47%)	(100.00%)
Region	Eastern region	26	7	2	35
		(20.63%)	(5.56%)	(1.59%)	(27.78%)
	Central region	13	7	0	20
		(10.32%)	(5.56%)	(0.00%)	(15.87%)
	Western region	21	12	0	33
		(16.67%)	(9.52%)	(0.00%)	(26.19%)
	Northeast region	4	1	0	5
		(3.17%)	(0.79%)	(0.00%)	(3.97%)
	Unspecified	19	6	8	33
		(15.08%)	(4.76%)	(6.35%)	(26.19%)
	Total	83	33	10	126
		(68.60%)	(27.30%)	(4.10%)	(100.00%)
Age	Elderly	10	2	0	12
		(7.75%)	(1.55%)	(0.00%)	(9.30%)
	Non-elderly	6	0	0	6
		(4.65%)	(0.00%)	(0.00%)	(4.65%)
	Unspecified	71	30	10	111
		(55.04%)	(23.26%)	(7.75%)	(86.05%)
	Total	87	32	10	129
		(67.44%)	(24.81%)	(7.75%)	(100.00%)
Income	Low-income	17	6	4	27
		(11.97%)	(4.23%)	(2.82%)	(19.01%)
	Non-low-income	12	4	2	18
		(8.45%)	(2.82%)	(1.41%)	(12.68%)
	Unspecified	65	26	6	97
		(45.77%)	(18.31%)	(4.23%)	(68.31%)
	Total	94	36	12	142
		(66.20%)	(25.35%)	(8.45%)	(100.00%)
Disease types	CCVD	5	4	2	11
		(3.85%)	(3.08%)	(1.54%)	(8.46%)
	Diabetes	4	1	0	5
		(3.08%)	(0.77%)	(0.00%)	(3.85%)
	Cancer	3	1	0	4
		(2.31%)	(0.77%)	(0.00%)	(3.08%)
	Infectious disease	4	1	0	5
		(3.08%)	(0.77%)	(0.00%)	(3.85%)
	Unspecified chronic disease	9	4	2	15
		(6.92%)	(3.08%)	(1.54%)	(11.54%)
	Non-chronic disease	3	0	0	3
		(2.31%)	(0.00%)	(0.00%)	(2.31%)
	Unspecified	57	24	6	87
		(43.85%)	(18.46%)	(4.62%)	(66.92%)
	Total	85	35	10	130
		(65.38%)	(26.92%)	(7.69%)	(100.00%)
Medical insurance	NCMS	28	7	4	39
		**Cross sectional**	**Case control**	**Cohort**	**Total**
		(21.21%)	(5.30%)	(3.03%)	(29.55%)
	UEBMI	3	2	0	5
		(2.27%)	(1.52%)	(0.00%)	(3.79%)
	URBMI	3	2	0	5
		(2.27%)	(1.52%)	(0.00%)	(3.79%)
	CMI	1	0	0	1
		(0.76%)	(0.00%)	(0.00%)	(0.76%)
	Unspecified	52	24	6	82
		(39.39%)	(18.18%)	(4.55%)	(62.12%)
	Total	87	35	10	132
		(65.91%)	(26.52%)	(7.58%)	(100.00%)
Total		81	32	5	118
		(68.64%)	(27.12%)	(4.24%)	(100.00%)

CHE, catastrophic health expenditure; CCVD, cardio-cerebrovascular diseases; NCMS, New rural cooperative medical scheme; UEBMI, Basic health insurance for urban employees; URBMI, Basic health insurance for urban residents; CMI, Commercial medical insurance.

Definition 1 (family out-of-pocket health expenditure exceeding 40% of household income or expenditure within a certain period), Definition 2 (family out-of-pocket expenditure on medical and health exceeding 40% of household nonfood expenditure), Definition 3 (the ratio of household medical and health expenditure to household consumption expenditure exceeds 40%), and Definition 4 (others).

Among the 118 studies included, 21 were published in English, and 97 were published in Chinese. The number of studies with a research period of 2008 and before, 2009–2012, 2013–2014, 2015–2016, and 2017–2020 were 14 (11.38%), 22 (17.89%), 25 (20.33%), 29 (23.58%) and 33 (26.83%), respectively. There were 73 (57.03%) studies conducted in rural areas, 15 (11.72%) in urban areas, and 40 (31.25%) studies that did not specify whether they were rural or urban. There were 35 (27.78%) studies conducted in the eastern region, 20 (15.78%) studies in the central region, 33 (26.19%) studies in the western region, 5 (3.97%) studies in the northeast region, and 33 (26.19%) studies that did not specify the area. There were 12 (9.30%) studies for elderly residents, 6 (4.65%) studies for non-elderly residents, and 111 (86.05%) studies that did not specify the age of the residents. Twenty-seven (19.01%) of the studies targeted low-income residents, 18 (12.68%) targeted non-low-income residents, and 97 (68.31%) of the studies did not specify the income of the residents. Eleven (8.46%) studies were conducted on residents with cardiovascular disease, 5 (3.85%) with diabetes, 4 (3.08%) with cancer, 5 (3.85%) with chronic infectious diseases, 15 (11.54%) with unspecified chronic diseases, 87 (66.92%) on residents with unspecificed health status and 3 (2.31%) of the studies for residents without chronic diseases. NCMS was studied in 39 (29.55%) studies, UEBMI in 5 (3.79%), URBMI in 5 (3.79%), CMI in 1 (0.76%), and 82 (62.12%) studies did not specify the type of health insurance (Table 1).

Among all 118 studies included, 30 (25.6%) studies adopted the criterion of “family OOP health expenditure exceeding 40% of household income or expenditure within a certain period.” Fifty-six (47.2%) studies adopted the World Bank's definition standard, that is, “family OOP expenditure on medical and health accounts for more than 40% of household non-food expenditure.” Seventeen (14.5%) studies adopted the standard definition of “the ratio of household medical and health expenditure to household consumption expenditure exceeds 40%.” In addition, 15 (12.7%) studies took into account the characteristics of the research objects, such as “OOP medical expenditures exceed farmers' annual per capita net income” (Table 1).

### Rate of catastrophic health expenditure in the last two decades

There was significant heterogeneity among the included studies; thus, a random-effect model was used. For all included studies, the pooled CHE rate was (25.2%, 95% CI: 23.4%−26.9%) from 2000 to 2020 (Appendix Figure 1 in Supplementary material). The general population's pooled CHE rate from 2000 to 2020 was 19.5% (95% CI: 17.2%−21.8%) (Figure 3).

### Subgroup analysis

#### Subgroup analysis of time trend

**Figure 2:**
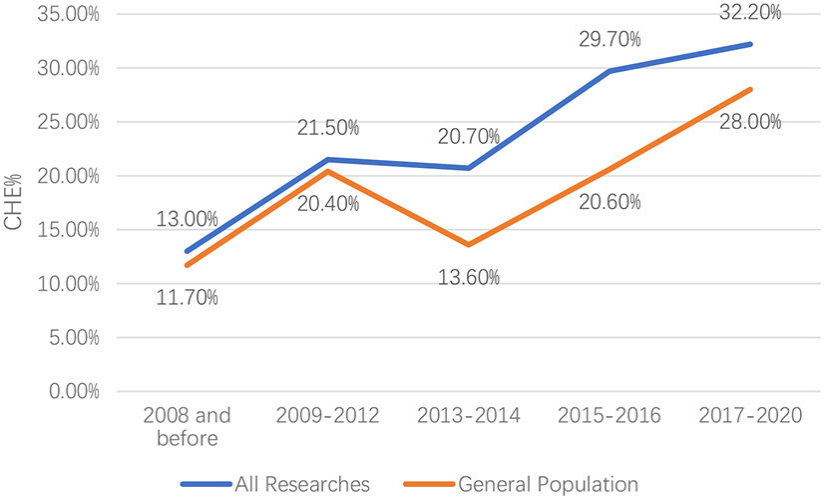
Secular trend of the rate of catastrophic health expenditures in China 2000−2020.

There was significant heterogeneity among the included studies; thus, a random-effect model was used. In the past two decades, the CHE rate in China has been rising. For all included studies, the CHE rate increased from 13.0% (95% CI: 10.6%−15.5%) in 2000–2008 to 32.2% (95% CI: 25.8%−38.7%) in 2017–2020, with only a short-term decrease from 21.5% (95% CI: 19.0%−24.0%) in 2009–2012 to 20.7% (95% CI: 16.3%−25.1%) in 2013–2014. For the general population, the CHE rate increased from 11.7% (95% CI: 9.1%−14.3%) in 2000–2008 to 28.0% (95% CI: 19.2%−36.8%) in 2017–2020, with only a short-term decrease from 20.4% (95% CI: 15.3%−25.4%) in 2009–2012 to 13.6% (95% CI: 9.3%−18.0%) in 2013–2014 (Figure 2).

#### Subgroup analysis of urban–rural differences

The CHE rate in urban areas was significantly higher than that in rural areas (*p* < 0.05). The CHE rate was 24.9% (95% CI: 23.2%−26.6%) for rural residents, 25.5% (95% CI: 20.4%−30.6%) for the unspecified group and 27.8% (95% CI: 22.0%−33.5%) for urban residents. There was significant heterogeneity among the included studies; thus, a random-effect model was used (Table 2).

**Table 2:** Subgroup analysis of the rate of catastrophic health expenditures.

**Group**		**No. of studies**	**CHE rate**	**(95% CI)**		**Weight (%)**	***p***
Urban-rural differences	Urban	15	0.278	0.220	0.335	11.80	<0.05
	Rural	73	0.249	0.232	0.266	57.03	
	Unspecified group	40	0.241	0.196	0.285	31.17	
Level of socioeconomic status	Western region	33	0.261	0.216	0.306	26.21	<0.05
	Northeast region	5	0.248	0.160	0.335	3.99	
	Eastern region	35	0.238	0.211	0.265	27.61	
	Central region	20	0.235	0.114	0.357	15.73	
	Unspecified group	33	0.241	0.196	0.285	26.45	
Age differences	Elderly	12	0.288	0.229	0.384	9.11	<0.05
	Non-elderly	6	0.225	0.095	0.354	4.44	
	Unspecified group	111	0.249	0.232	0.267	86.45	
Income differences	Low-income	26	0.297	0.213	0.381	18.32	<0.05
	Non-low-income	18	0.173	0.138	0.208	12.46	
	Unspecified group	98	0.249	0.225	0.273	69.22	
Diseases differences	Cancer	4	0.489	0.396	0.582	2.87	<0.05
	Chronic infectious disease	5	0.360	0.243	0.476	3.76	
	CCVD	10	0.353	0.274	0.433	8.47	
	Diabetes	5	0.272	0.147	0.396	3.70	
	Unspecific chronic disease	14	0.235	0.176	0.293	11.51	
	Non-chronic disease	3	0.101	0.041	0.161	2.34	
	Unspecified group	83	0.236	0.215	0.257	67.44	
Medical insurance	NCMS	39	0.171	0.160	0.181	29.81	<0.05
	UEBMI	4	0.118	0.083	0.153	3.00	
	URBMI	5	0.106	0.054	0.157	3.65	
	CMI	1	0.194	0.054	0.333	0.52	
	Unspecified group	83	0.287	0.252	0.322	63.02	
Definition of CHE	Definition 1	30	0.298	0.248	0.349	25.59	<0.05
	Definition 2	56	0.268	0.223	0.313	47.23	
	Definition 3	17	0.152	0.107	0.196	14.53	
	Definition 4	15	0.228	0.201	0.254	12.66	

CHE, catastrophic health expenditure; CCVD, cardio-cerebrovascular diseases; NCMS, New rural cooperative medical scheme; UEBMI, Basic health insurance for urban employees; URBMI, Basic health insurance for urban residents; CMI, Commercial medical insurance.

Definition 1 (family out-of-pocket health expenditure exceeding 40% of household income or expenditure within a certain period), Definition 2 (family out-of-pocket expenditure on medical and health exceeding 40% of household nonfood expenditure), Definition 3 (the ratio of household medical and health expenditure to household consumption expenditure exceeds 40%), and Definition 4 (others).

#### Subgroup analysis of regional differences

By region, the CHE rate was highest in the western region (26.1%, 95% CI: 21.6%−30.6%), followed by the northeast region (24.8%, 95% CI: 21.1%−26.5%). In comparison, the CHE rate was similar in the central region (23.5%, 95% CI: 11.4%−35.7%) and the eastern region (23.8%, 95% CI: 22.7%−28.4%), both lower than the western region and the northeast region. The difference was statistically significant (*p* < 0.05). There was significant heterogeneity among the included studies; thus, a random-effect model was used (Table 2).

#### Subgroup analysis of age differences

The CHE rate was significantly higher in the elderly (28.8%, 95% CI: 22.9%−34.8%) than in the non-elderly (22.5%, 95% CI: 9.5%−34.5%), and the CHE rate in the unspecified group was 24.9%, 95% CI: 23.2%−26.7%. There was significant heterogeneity among the included studies; thus, a random-effect model was used (Table 2).

#### Subgroup analysis of income differences

The CHE rate in the low-income group (29.7%, 95% CI: 21.3%−38.1%) was significantly higher than that in the non-low-income group (17.3%, 95% CI: 13.8%−20.8%), while that in the unspecified group was (24.9%, 95% CI: 22.5%−27.3%). There was significant heterogeneity among the included studies; thus, a random-effect model was used (Table 2).

#### Subgroup analysis of disease differences

The CHE rates in the cancer group (48.9%, 95% CI: 39.6%−58.2%), chronic infectious disease group (36.0%, 95% CI: 24.3%−47.6%) and CCVD group (35.3%, 95% CI: 27.4%−43.3%) were significantly higher than those of the other groups. The CHE rate in the non-chronic disease group (10.1%, 95% CI: 4.1%−16.1%) was significantly lower than that in the other groups. There was significant heterogeneity among the included studies; thus, a random-effect model was used (Table 2).

#### Subgroup analysis of medical insurance differences

Regarding insurance type, the CHE rates of URBMI (10.6%, 95% CI: 5.4%−15.7%) and UEBMI (11.8%, 95% CI: 8.3%−15.3%) were slightly lower than that of the NCMS (17.1%, 95% CI: 16.0%−18.1%). There was significant heterogeneity among the included studies; thus, a random-effect model was used (Table 2).

#### Subgroup analysis of CHE definition differences

According to the meta-analysis results, different definitions of CHE impact the CHE rate. The CHE rate in definition 1 group was (29.8%, 95% CI: 24.8%−34.9%); definition 2 was (26.8%, 95% CI: 22.3%−31.3%); definition 3 was (15.2%, 95% CI: 10.7%−19.6%); definition4 was (22.8%, 95% CI: 20.1%−25.4%). There was significant heterogeneity among the included studies; thus, a random-effect model was used (Table 2).

### Meta-regression analysis

Multiple meta-regression analyses found that low-income (beta = 0.12, 95% CI: 0.00–0.23, *p* = 0.043), cancer (beta = 0.40, 95% CI: 0.11–0.69, *p* = 0.007), CCVD (beta = 0.25, 95% CI: 0.01–0.50, *p* = 0.044), unspecified medical insurance type (beta = 0.17, 95% CI: 0.00–0.34, *p* = 0.050), definition 1 (beta = 0.15, 95% CI: 0.03–0.26, *p* = 0.013) and definition 2 (beta = 0.12, 95% CI: 0.01–0.22, *p* = 0.030) were correlated with the CHE rate, while other factors were all non-significantly correlated (Appendix Table 2 in Supplementary material).

### Sensitivity analyses

Dropping the influential study, the outcome was 25.3% (95% CI: 23.5%−271%), which was close to the primary outcome (25.2%, 95% CI: 23.4%−26.9%). Therefore, dropping the influential study did not change the inference for the primary outcomes.

### Quality assessment and certainty of evidence

We divided studies with an NOS score of <5 points into low-quality research, 5–8 points into medium-quality research, and 8–9 points into high-quality research. In the case-control study, there were 5 low-quality studies, 26 medium-quality studies and 1 high-quality study. All cohort studies were of medium quality. Among the cross-sectional studies included, 86% had a quality score of 4–6. Generally, the quality of the studies included in this study was moderate (Appendix Table 3 in Supplementary material). Using GRADE, we judged the certainty in our estimates to be low across outcomes. We downgraded evidence for high inconsistency, high publication bias and by one level for serious risk of bias (Appendix Table 4 in Supplementary material).

### Publication bias analysis

**Figure 3:**
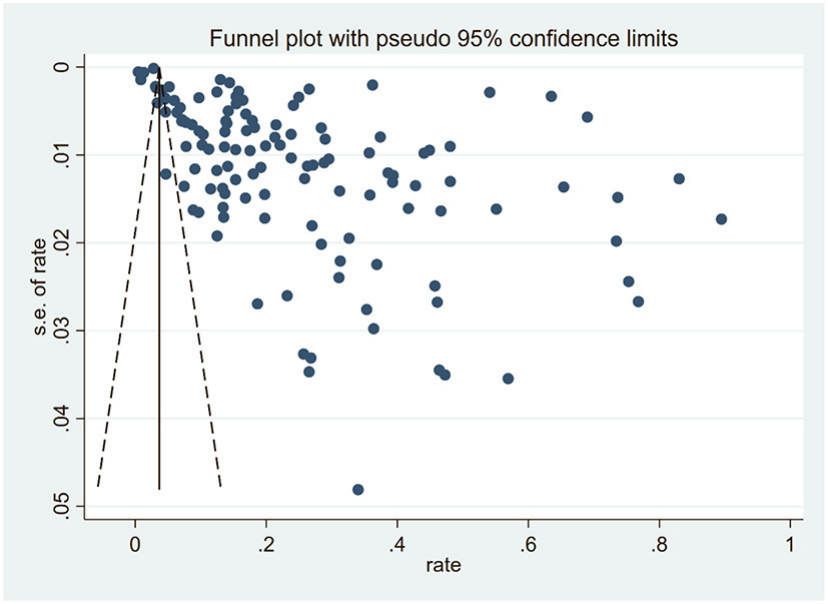
Publication bias funnel plot.

The funnel plot suggested that there was a publication bias in this study because a large number of included studies were biased to the right of the funnel plot (Figure 3). Egger's test indicated that the studies included had publication bias (*t* = 8.19, *p* < 0.01). The trim-and-fill analysis estimated that 62 studies were missing, and the effect size generated after the inclusion of these 62 studies was significantly changed from the original model (5.5%, 95% CI: 3.7%−7.4%, *p* < 0.01) (Appendix Figure 3 in Supplementary material). However, the publication bias of this study may be partly due to the existence of significant heterogeneity in the included studies.

## Discussion

There were seven main findings in this study. First, the overall CHE rate in China remained high. Second, the CHE rate continued to rise. Third, the CHE rate in underdeveloped areas is higher than that in other regions. Fourth, the CHE rate in the elderly was higher than that in the non-elderly. Fifth, the CHE rate in the low-income group was higher than that in the non-low-income group. Sixth, the CHE rate was higher in people with critical illnesses, such as cancer, CCVDs, diabetes, chronic hepatitis, AIDS and other diseases. Last, different definitions of CHE affected the CHE rate; CHE is the highest in definition 1 (family OOP health expenditure exceeding 40% of household income or expenditure within a certain period) and higher in definition 2 (family OOP expenditure on medical and health exceeding 40% of household non-food expenditure) than in definition 3 (the ratio of household medical and health expenditure to household consumption expenditure exceeds 40%).

The CHE rates found in this study at different periods were higher than those in the previous study. One study based on CFPS showed that in 2010, 2012, 2014 and 2016, the CHE rates in China were 13.58, 11.95, 11.43 and 11.06%, respectively (5). This may be because CFPS is a longitudinal survey project that attempts to gather information on a nearly nationally representative sample of families, and all members of those families were well represented (18). However, CFPS aims to reflect social, economic, demographic, educational and health changes in China (19) and was not a study specifically designed to study the CHE of Chinese residents. The CFPS used a self-rated health score to measure the health status of the study subjects, which may result in the selected sample being underrepresented in measuring CHE. In our study, nearly 35% of the included studies were carried out on people who suffered from specific diseases, and there were also studies on elderly people, low-income people and other people with a high CHE rate; therefore, the CHE rate may be overestimated. However, a meta-analysis was performed based on the included studies of the general population, and the findings were lower than the overall CHE incidence but still higher than those based on CFPS. Therefore, the different findings may be explained not only by the varied characteristics of participants but also by methodological heterogeneities.

There are many reasons for the continued increase in the CHE rate in China in the past two decades. The sustained and rapid health expenditure growth may be the most important reason for the continuous increase in the CHE rate in China. Health expenditure per capita in China has risen rapidly in the past two decades. From 2013 to 2019, the annual growth rate of per capita health care expenditure in China was higher than the growth rate of personal disposable income (20). Although the proportion of total OOP health expenditures to total health expenditures has decreased, the burden of actual total OOP health expenditures was still increasing (20, 21). The total health expenditure in China also increased rapidly. From 2008 to 2017, the average annual rate of real growth of total health expenditure in China was 12.2%, which exceeded the average annual rate of real growth of GDP. The rapid increase in health expenditure largely diminished the protective role of social health insurance in reducing CHE.

The findings of factors associated with the CHE rate in this study were in accordance with previous studies. A systematic review of CHE in Asian countries found that family economic status, hospitalization rate, whether there are elderly people in the family and the status of chronic diseases impacted the CHE rate. Research in India reached a similar conclusion and found that the CHE rate in India was also rising (22, 23). A systematic review of Iran pointed out that patients with chronic diseases, especially cancers, have a higher CHE rate than the general population. From 2001 to 2015, Iran's CHE rate also showed a continuously rising trend (24). These research findings were consistent with the findings in this study, which indicated similar challenges of CHE and its risk factors in developing countries.

First, the unreasonable use of hospital services may be another important reason for the rising CHE rate in China. In the past 20 years, the hospitalization rate in China rose rapidly, from 4.2% in 2000, to 10.59% in 2010 and 19.03% in 2019, double that of a decade ago and quadrupling that of 20 years ago. Mark W. Moses pointed out that the hospitalization rate in high-income countries, middle-income countries and low-income countries has been relatively stable over the past 10 years, while the hospitalization rate in China has grown sharply (25). China is a middle-income country, but the hospitalization rate is higher than in many high-income countries. In 2016, the inpatient admissions per capita in China were 0.14, 40% higher than the global average (0.10).

One reason for the rapid increase of hospitalization in China is that the remuneration rate of hospitalization expenditures by the national basic medical insurance has been steadily increasing in the past two decades, which gives incentives to health providers and patients for hospitalization; however, there are no effective measures, such as the legitimate gate-keeping role of primary health care, to promote the reasonable hospitalization behavior of patients. In addition, with the improvement in the coverage and security level of medical insurance, Chinese residents were more inclined to seek medical treatment in high-end medical institutions (26). Participating in medical insurance can improve the utilization rate of medical services, but it did not significantly reduce the proportion of OOP medical expenditure (27). The overuse of medical treatment behaviors also increased the CHE rate (28).

Second, in the past two decades, the disease spectrum in China has changed significantly, in which chronic non-communicable diseases have been the main burden of diseases. The main causes of disability-adjusted life-years (DALYs) have transformed into CCVD, cancer, pain and depression, while the years of life lost (YLLs) caused by neonatal death, infectious diseases and injury have been significantly reduced (29). The increasing prevalence and cost of chronic diseases and the burden of diseases have brought huge challenges to the health care system in China. The CHE rate was very high for patients with critical illnesses. Chen and colleagues found that cerebrovascular disease, hypertension, cancer, coronary heart disease, diabetes, obstructive emphysema, severe psychosis, liver cirrhosis, chronic bronchitis and kidney disease were the top 10 diseases that cause CHE, which was consistent with the findings of our study (30). Among them, cancer patients had the highest CHE rate in China due to the excessive use of life-prolonging treatments (31). China's early cancer screening rate was low, and most patients were already in the advanced stages of cancer when they were diagnosed. In 2020, China accounted for ~3 million of the estimated 9.96 million cancer deaths worldwide, accounting for 30.2% of cancer deaths worldwide. This has led to an increase in the cost of cancer treatment in China (32, 33). Currently, China is reducing the OOP expenditure on cancer treatment by including more innovative cancer drugs in the National Catalog of Medical Insurance (NRLD) and using centralized procurement to reduce their prices. This move can significantly reduce the economic burden of drugs for cancer patients, but the extent to which it can reduce the risk of CHE for cancer patients still needs further study (34).

Third, population aging may be another driver of the increased CHE in China. The health status of the elderly population was poorer than that of the younger population, and the prevalence of chronic diseases in the elderly was high. In China, people with at least one chronic disease account for more than 75% of all elderly people. Suffering from multiple diseases has increasingly become a severe problem. Over the past five years, the comorbidity rate among China's elderly population has increased from 41 to 43% (35). Multiple diseases can significantly increase the risk of CHE, even for those with health insurance coverage and high socioeconomic status (36). Due to poor health conditions, the utilization of health services by the elderly continued to increase, especially hospitalization, which increased from 11.5% in 2011 to 16.4% in 2015. The hospitalization expenditure per capita of the elderly also increased from 4,225 RMB in 2011 to 6,000 RMB in 2015. However, the hospitalization expenditure per capita compensated by medical insurance increased from 2,400 RMB in 2011 to 3,000 RMB in 2015 (37). This undoubtedly increases the OOP medical expenditures of elderly individuals.

Fourth, China's medical insurance system was still suboptimal. Although China's basic health insurance systems have expanded to almost the entire population and the reimbursement rate has steadily increased in the past two decades, there are still some deficiencies (38). For one thing, the financing level of basic health insurance in China was not enough to meet the growing needs of health care and protect the insured from the risk of poverty due to illness (39). In addition, China's critical illness insurance (CII) system needs to be improved. In 2013, China began to implement a CII plan. Of the NCMS personnel, 1.23 million received compensation for CII. Based on the basic compensation of the NCMS, the actual reimbursement rate for critical illness patients has increased by ~12% (40). However, the coverage of CII was relatively small, with only 20 diseases. In some areas, CII schemes also set a ceiling that does not cover all medical expenditures.

Fifth, we found that different definitions profoundly impacted the estimate of the CHE rate in the included studies. Since Chinese households generally have the habit of saving, it is more in line with China's spending habits to use income to calculate a household's ability to pay. Therefore, the adoption of the criterion of “mandatory household OOP health expenditure exceeds 40% of household income or expenditure within a certain period” may be best suited to the actual situation in China, and the adoption of “household health expenditure exceeds 40 percent of household consumption expenditure” may overestimate the incidence of CHE in China (41). Regardless of the definition adopted, an agreement on a unified definition of CHE may be needed for future research on CHE to facilitate national and international comparisons.

Finally, the low-income population suffered more from poor health. According to statistics from the State Council's Poverty Alleviation Office in 2016, among the poor households in China, “Poverty due to illness and return to poverty due to illness” accounted for 42.2% of the total number of poverty-stricken families registered, and the CHE rate was relatively high. The health of the low-income population in China was poor, and the utilization rate of health services was low. The 2-week prevalence rate and the 2-week chronic disease prevalence rate of the poor population were higher than the overall population level; the self-rated health score was only 76.6 points, lower than the general population level. In terms of health service demand, the 2-week consultation rate and hospitalization rate of the poor population were higher than those of the entire population, indicating that the poor population suffers more health problems; and the proportion of low-income people who needed to be hospitalized but not hospitalized was much higher than that at the population level, indicating that their health service utilization rate was low (42). In 2016, China began implementing a new health poverty alleviation policy. Health and poverty alleviation provided multiple protections for the poor, including basic medical insurance, CII and medical assistance (43). The effects of basic medical insurance, CII and critical illness relief could effectively curb the CHE rate. The poverty reduction function in descending order was the NCMS, CII, and medical assistance insurance. However, the combined effect of the three kinds of medical insurance on poverty alleviation was still not strong because the population covered by the three types of medical insurance accounts for only ~5% of the total population (44).

## Strengths and limitations

This study had several strengths. First, to the best of our knowledge, this is currently the most comprehensive systematic review that explores the CHE trend and its influencing factors in China in the last two decades. Second, this study complied with internationally recognized methodological guidelines and standards with methodological rigor. However, this study has several limitations. First, there was significant heterogeneity between the studies included, which could not be solely explained by the varied study characteristics explored. Second, in specific subgroup analyses, many studies were grouped into unspecified groups due to missing data, and there was a significant publication bias, which may affect the reliability of the results. Third, the overall quality of the literature included in this study was moderate.

## Conclusion

To control the CHE rate, it is necessary to introduce relevant policies to control the rapid rise in health expenditures, guide reasonable medical treatment, and avoid unnecessary hospitalization. First, the National Catalogue of Medical Insurance (NRLD) needs to be further revised to include more drugs and treatments that could lead to CHE and to reduce the price of drugs and medical materials. Second, the financing level of medical insurance needs to be further improved to strengthen the protective role of basic medical insurance. Third, it is also essential to further advance the reform of the hospital payment method, for example, bundle payment methods (DRGs or DIP payment), to control the excessive growth of health expenditures. Based on the existing evidence, our systematic review and meta-analysis suggest that CHE may aggravate the incidence of poverty, which in turn may aggravate the incidence of CHE. In 2020, China achieved complete poverty alleviation, and the relationship between health poverty alleviation and CHE reduction needs further evaluation.

## Data availability statement

The original contributions presented in the study are included in the article/Supplementary material, further inquiries can be directed to the corresponding authors. And authors did not detect any particular expressions.

## Author contributions

FZ was responsible for formal analysis, data curation, writing—original draft, and writing—review and editing. JJ was responsible for methodology and writing—review and editing. MY was responsible for writing—review and editing. KZ was responsible for conceptualization, methodology, writing—review and editing, and supervision. DC was responsible for methodology, writing—review and editing, project administration, and funding acquisition. All authors read and approved the final manuscript.

## References

[1] Xu K Evans DB Kawabata K Zeramdini R Klavus J Murray CJ. Household catastrophic health expenditure: a multicountry analysis. Lancet. (2003) 362:111–7. 10.1016/S0140-6736(03)13861-5 12867110

[2] Daneshkohan A Karami M Najafi F Matin BK. Household catastrophic health expenditure. Iran J Public Health. (2011) 40:94–9. 23113061 PMC3481728

[3] Xu K. Distribution of health payments and catastrophic expenditures. In: Distribution of Health Payments and Catastrophic Expenditures Methodology (2004).

[4] Gabriela F Daniel H Gretchen S Justine H Sarah T Tamás E . Tracking Universal Health Coverage: 2017 Global Monitoring Report. Geneva: World Health Organization and World Bank (2017).

[5] Yip W Fu H Chen AT Zhai T Jian W Xu R . 10 years of health-care reform in China: progress and gaps in Universal Health Coverage. Lancet. (2019) 394:1192–204. 10.1016/S0140-6736(19)32136-1 31571602

[6] Li L. Review of the progress of the new medical reform. China Health Econ. (2012) 31:5–9. 10.3969/j.issn.1003-0743.2012.01.002

[7] Jiang JH. The impact of the abolition of the drug markup policy on medical costs. Med Philos. (2010) 31:44–6.

[8] Zheng C Wang X Sun Q. Research on urban and rural medical insurance overall policy, health risk impact and precise poverty alleviation performance. J Public Admin Manag. (2021) 19:1–16. 10.16149/j.cnki.23-1523.20211009.001

[9] Wang J. Investigation and analysis of the impact of the national essential drugs system on the medical expenses of patients in different levels of medical and health institutions. China Pharm. (2012) 23:2982–4.

[10] Zhou M Wang H Zeng X Yin P Zhu J Chen W . Mortality, morbidity, and risk factors in China and its provinces, 1990–2017: a systematic analysis for the Global Burden of Disease Study 2017. Lancet. (2019) 394:1145–58. 10.1016/S0140-6736(19)30427-1 31248666 PMC6891889

[11] Wang L. Analysis of the prevalence of chronic diseases in the elderly and their influencing factors. Chin J Epidemiol. (2019) 40:277–83.

[12] Guo F Zhang Y Wan Q Zhai T Chai P Li Y . Results and analysis of total health expenditure in China in 2017. China Health Econ. (2019) 38:5–8.

[13] Xu W Chu F. A study on the level and influencing factors of catastrophic health expenditure: an analysis based on CHARLS data. Social Security Res. (2018) 5:64–72. 10.3969/j.issn.1674-4802.2018.05.007

[14] Wang X Wang H. Evaluation of the effect of basic medical security system on improving catastrophic health expenditure. Chin J Public Health. (2017) 33:901–4. 10.11847/zgggws2017-33-06-09

[15] Hu C Wang Y Wang J Zhou W Zhou D Du X . Analysis of the effect of drug addiction cancellation on medical costs. China Health Quality Manage. (2020) 27:32–5.

[16] Bai Z Zhang X He B. Comparison of urbanization development characteristics and trends of four major economic regions in China. Soft Sci. (2009) 22:104–8. 10.3969/j.issn.1001-8409.2009.01.020

[17] Sun J Zhao Q A. Study on the measurement of income distribution fairness in China: A comparative analysis based on panel data in East, Central and Western China. J Fin Econ. (2017) 2:18–27. 10.3969/j.issn.1004-4892.2017.02.003

[18] Xie Y Lu P. The sampling design of the China Family Panel Studies (CFPS). Chin J Sociol. (2015) 1:471–84. 10.1177/2057150X15614535 29854418 PMC5973535

[19] Xie Y Hu J Zhang C. A longitudinal survey of Chinese families: philosophy and practice. Sociology. (2014) 34:1–32.

[20] Cui H Chen D Gao J. A comparative analysis of the structural characteristics of total health cost financing and per capita disposable income in China. China Health Policy Res. (2017) 10:64–9. 10.3969/j.issn.1674-2982.2017.05.011

[21] Guo B Cheng H Liu Y. Research on the change trend of personal health expenditure and its proportion in China. Med Soc. (2014) 27:46–8. 10.13723/j.yxysh.2014.06.01531199806

[22] Pandey A Ploubidis GB Clarke L Dandona L. Trends in catastrophic health expenditure in India: 1993 to 2014. Bull World Health Organ. (2018) 96:18–28. 10.2471/BLT.17.191759 29403097 PMC5791868

[23] Rijal A Adhikari TB Khan JAM Berg-Beckhoff G. The economic impact of non-communicable diseases among households in South Asia and their coping strategy: a systematic review. PLoS ONE. (2018) 13:e205745. 10.1371/journal.pone.0205745 30462648 PMC6248902

[24] Doshmangir L Yousefi M Hasanpoor E Eshtiagh B Haghparast-Bidgoli H. Determinants of catastrophic health expenditures in Iran: a systematic review and meta-analysis. Cost Effect Resour. (2020) 18:1–21. 10.1186/s12962-020-00212-0 32467673 PMC7229629

[25] Moses MW Pedroza P Baral R Bloom S Brown J Chapin A . Funding and services needed to achieve universal health coverage: applications of global, regional, and national estimates of utilisation of outpatient visits and inpatient admissions from 1990 to 2016, and unit costs from 1995 to 2016. Lancet Public Health. (2019) 4:e49–73. 10.1016/S2468-2667(18)30213-5 30551974 PMC6323358

[26] Wagstaff A Lindelow M Jun G Ling X Juncheng Q. Extending health insurance to the rural population: an impact evaluation of China's new cooperative medical scheme. J Health Econ. (2009) 28:1–19. 10.1016/j.jhealeco.2008.10.007 19058865

[27] Wagstaff A Yu S. Do health sector reforms have their intended impacts? J Health Econ. (2007) 26:505–35. 10.1016/j.jhealeco.2006.10.006 17112613

[28] Wu Y Li J Chao-Hsien C. Evolution of excessive medical behavior under drug ratio control. Syst Eng Theory Pract. (2019) 39:3163–75. 10.12011/1000-6788-2018-1930-1335777803

[29] Gonghuan Y Yu W Yixin Z George FG Xiaofeng L Maigeng Z . Rapid health transition in China, 1990–2010: findings from the Global Burden of Disease Study 2010. Lancet. (2013) 381:9882. 10.1016/S0140-6736(13)61097-1 23746901 PMC7159289

[30] Chen H Wang T Wang X Liu Q. Analysis of the current situation of poverty caused by disease in rural Yulin City and the characteristics of poverty diseases. Modern Prev Med. (2019) 46:3754–7, 3763.31.

[31] Leng A Jing J Nicholas S Wang J. Catastrophic health expenditure of cancer patients at the end-of-life: a retrospective observational study in China. BMC Palliat Care. (2019) 18:43. 10.1186/s12904-019-0426-5 31122235 PMC6533646

[32] Zhao Y Zhang L Fu Y Wang M Zhang L. Socioeconomic disparities in cancer treatment, service utilization and catastrophic health expenditure in china: a cross-sectional analysis. Int J Environ Res Public Health. (2020) 17:1327. 10.3390/ijerph17041327 32092913 PMC7068279

[33] Deng P Fu Y Chen M Si L. Factors associated with health care utilization and catastrophic health expenditure among cancer patients in China: evidence from the China health and retirement longitudinal study. Front Public Health. (2022) 10:943271. 10.3389/fpubh.2022.943271 36438282 PMC9684646

[34] Yin S Zhang H Liu Y. Policy implementation effect and thinking of tumor innovative drugs included in the national medical insurance catalog. China Health Economics. (2021) 40:22–4.

[35] Wang M Zhou X Li Y Liu F Yao Z. Meta-analysis of the prevalence of chronic diseases in middle-aged and elderly people in China, 2010–2019. Chin J General Pract. (2021) 24:2085–91. 10.12114/j.issn.1007-9572.2020.00.477

[36] Zhao Y Atun R Oldenburg B McPake B Tang S Mercer SW . Physical multimorbidity, health service use, and catastrophic health expenditure by socioeconomic groups in China: an analysis of population-based panel data. Lancet Glob Health. (2020) 8:e840–9. 10.1016/S2214-109X(20)30127-3 32446349 PMC7241981

[37] Li Y Chen X Gao J Feng Z. Study on the direct economic burden of diseases in middle-aged and elderly patients with chronic diseases in China. China Health Econ. (2019) 38:71–3.

[38] Li F Wu Y Yuan Q Zou K Yang M Chen D. Do health insurances reduce catastrophic health expenditure in China? A systematic evidence synthesis. Plos ONE. (2020) 15:e239461. 10.1371/journal.pone.0239461 32970740 PMC7514005

[39] Li A Shi Y Yang X Wang Z. Effect of critical illness insurance on household catastrophic health expenditure: the latest evidence from the National Health Service Survey in China. Int J Env Res Pub Health. (2019) 16:5086. 10.3390/ijerph16245086 31847072 PMC6950570

[40] Liang X Guo H Jin C Peng X Zhang X. The effect of new cooperative medical scheme on health outcomes and alleviating catastrophic health expenditure in China: a systematic review. PLoS ONE. (2012) 7:e40850. 10.1371/journal.pone.0040850 22916098 PMC3423411

[41] Tao S Zhao Y Wan Q Zhang Y Huang J. Research on analysis methods of catastrophic health expenditure. China Health Econ. (2004) 4:9–11. 10.3969/j.issn.1003-0743.2004.04.003

[42] Fang P Su M. On the key issues and system construction of health poverty alleviation in China. China Health Policy Res. (2017) 10:60–3. 10.3969/j.issn.1674-2982.2017.06.011

[43] Qiu Y Huang G. Research on the operation mechanism of serious illness insurance: based on domestic and foreign experience. Zhongzhou J. (2014) 1:61–6. 10.3969/j.issn.1003-0751.2014.01.014

[44] Bao Z Zhao Y. Research on the anti-poverty effect of rural residents' medical insurance: an empirical analysis based on PSM. J Jiangxi Univ Fin Econ. (2018) 1:90–105.

