# Supplementary material for: Catastrophic health expenditure, incidence, trend and socioeconomic risk factors in China: A systematic review and meta-analysis
Source: Front Public Health. 2023 Jan 4;10:997694. doi: 10.3389/fpubh.2022.997694 (PMC9846062; doi:10.3389/fpubh.2022.997694)
Supplement: Supplementary file 1 [file Table_1.DOCX]

AppendixTable 1. Search strategy in PubMed

| Keywords |
| --- |
| 1、Catastrophic health expenditure  2、Catastrophic medical expenses  3、Poverty-causing health expenditure  4、Poverty due to illness  5、Return to poverty due to illness  6、1 or 2 or 3 or 4 or 5  7、China  8、6 and 7 |

Appendix Table 2. Meta regression analysis of the influencing factors of catastrophic health expenditure

| Variable name | Coefficient | t | P | [95%Conf.Interval] | |
| --- | --- | --- | --- | --- | --- |
| rural | 0.00 | -0.07 | 0.947 | -0.08 | 0.07 |
| urban | 0.02 | 0.4 | 0.688 | -0.09 | 0.14 |
| Eastern region | 0.01 | 0.2 | 0.84 | -0.10 | 0.12 |
| North-east region | 0.16 | 1.83 | 0.07 | -0.01 | 0.33 |
| Western region | 0.03 | 0.56 | 0.574 | -0.08 | 0.14 |
| Unspecified group | 0.01 | 0.11 | 0.911 | -0.10 | 0.11 |
| Elderly | -0.04 | -0.42 | 0.674 | -0.24 | 0.15 |
| Unspecified group | -0.05 | -0.57 | 0.568 | -0.21 | 0.12 |
| Low-income | 0.12 | 2.04 | 0.043 | 0.00 | 0.23 |
| Unspecified group | 0.07 | 1.48 | 0.141 | -0.02 | 0.17 |
| Cancer | 0.40 | 2.73 | 0.007 | 0.11 | 0.69 |
| Cardio-cerebro-vascular diseases | 0.25 | 2.03 | 0.044 | 0.01 | 0.50 |
| Diabetes | 0.17 | 1.23 | 0.223 | -0.11 | 0.45 |
| Major infectious disease | 0.26 | 1.85 | 0.067 | -0.02 | 0.54 |
| Unspecific chronic disease | 0.14 | 1.13 | 0.262 | -0.10 | 0.38 |
| Unspecified group | 0.14 | 1.22 | 0.224 | -0.09 | 0.36 |
| NCMS | 0.07 | 0.81 | 0.42 | -0.10 | 0.25 |
| UEBMI | 0.00 | 0.01 | 0.992 | -0.25 | 0.25 |
| CMI | 0.08 | 0.36 | 0.721 | -0.35 | 0.50 |
| Unspecified group | 0.17 | 1.97 | 0.050 | 0.00 | 0.34 |
| Definition 1 | 0.15 | 2.53 | 0.013 | 0.03 | 0.26 |
| Definition 2 | 0.12 | 2.2 | 0.030 | 0.01 | 0.22 |
| Definition 4 | 0.09 | 1.36 | 0.175 | -0.04 | 0.23 |

Appendix Table 3. Characteristics of included studies

| Author/ year | region | study design | participants | N | definition of CHE | quality score |
| --- | --- | --- | --- | --- | --- | --- |
| HJJ2018(45) | Midu County, Yunnan Province, China | Cross-sectional | Eight townships/towns within the jurisdiction of Midu County, Yunnan Province, randomly sampled diabetic patients in multiple stages according to income level | 162 | Households have paid more than 40% of their disposable income (total annual household income - food and education expenditure) in the past year | 6 |
| SY2018(46) | Shaanxi Province, China | Cross-sectional | People with diabetes in the fifth National Health Service Survey conducted in Shaanxi Province from September to October 2013 | 209 | Household medical and health expenditure accounts for 40 percent of household ≥ income-expenditure | 5 |
| HSS2018(47) | Shandong Province, China | Cross-sectional | In Shandong Province, random sampling was stratified according to the economic level | 754 | Out-of-pocket medical expenses account for 40% of household non-food expenses | 6 |
| LXJ2018(48) | Jiangsu Province, China | Cross-sectional | China Health and Pension Tracking CHALS survey 2013 Jiangsu Province follow-up survey of people over 45 years of age | 1080 | Families pay for medical expenses in household non-food consumption spending exceeds 40% | 4 |
| LXM2017(49) | China | Cross-sectional | The Ministry of Education's major research project on philosophy and social sciences, "Perfecting the Research on social assistance system,” is a low-security survey | 1472 | Household out-of-pocket pays more than 40% of household consumption for health care | 6 |
| LF2017(50) | Shanghai, China | Cross-sectional | In 2015, shanghai civil medical assistance for low-insured, low-income families, severely disabled and unemployed, and particular relief objects for civil affairs | 55500 | Out-of-pocket medical expeness≥ 40% of total personal consumption expenditure | 3 |
| LQ2017(51) | Rural areas of Yunnan Province, China | Cross-sectional | From January to September 2015, a random sample of multi-stage stratified random samples according to economic level was selected to conduct a questionnaire survey in Yunnan Province | 3909 | For a certain period, households spend more on medical care than a certain proportion of disposable household capacity, which has a disastrous impact on average household consumption, at 40% standards. | 6 |
| HX2017(52) | Jilin Province, China | Cross-sectional | Low-income families identified by the local Civil Administration as having low-security families with chronic diseases who had been treated for chronic diseases in the year before the survey | 312 | Household out-of-pocket payments for medical expenses account for more than 40% of household non-food consumption expenditure | 4 |
| LXR2017(53) | Yinchuan City, Gansu Province, China | Cross-sectional | A random group sampling method to extract the residents of Yinchuan city's rural areas | 373 | Household health expenditure accounts for 40% of household non-food consumption | 6 |
| WY2018(54) | Fushun County, Sichuan Province, China | Cross-sectional | In Fushun County, Sichuan Province, a random sample of rural residents participating in the new agricultural joint was selected at multiple stages | 999 | Household out-of-pocket payments for health care account for more than 40% of household consumer spending | 6 |
| LYB2017(55) | Jiangsu Province, China | Cross-sectional | Randomly collected 400 patients who had been admitted to a Chinese medicine hospital in a city in Jiangsu Province in 2014 because of a severe illness | 400 | In this study, the occurrence of catastrophic expenditure on inpatients in rural residents was measured by the medical expenses of rural residents exceeding 3083 yuan | 2 |
| GMT2016(56) | Rural areas of Hubei Province, China | Cross-sectional | "Fifth Health Service Survey in Hubei Province" in 2013 | 7202 | Residents' out-of-pocket and health expenditures account for more than 40% of households' ability to pay | 5 |
| PMH2017(57) | Zhongjiang County, Sichuan Province, China | Cross-sectional | In the new agricultural joint information system, the information of the third-level hospital in Deyang City in 2010 was derived from the farmers participating in the county | 1902 | Out-of-pocket out-of-the-way expenses exceed the annual per capita net income of farmers | 6 |
| WH2016(58) | Qianxi County, Hebei Province, China | Cross-sectional | The health inquiry data of the new rural cooperative medical family were collected in the county of Qianxi, Hebei Province | 1581 | The ROC curve of the result of poverty due to disease is drawn by the burden of household health expenditure, which corresponds to the critical value of the burden of household health expenditure when the index is at its highest, which is 38% in this paper | 5 |
| ZYJ2016(59) | Sichuan Province, China | Cross-sectional | Rural families in Fushun County, Zigong, Sichuan Province, 2012 | 2244 | Household out-of-pocket hygiene expenditure accounts for 40% of household non-food consumption expenditure | 6 |
| WZH2016(60) | Rural areas of Jiangsu Province, China | Cross-sectional | Data from the 2013 survey on the economic burden of disease in rural areas of Jiangsu Province were relevant to the financial burden of illness in elderly chronically ill families | 1588 | Out-of-payments health care expenditure as a percentage of household consumption expenditure exceeds 40% | 5 |
| FZY2017(61) | A city in western Hubei Province, China | Cross-sectional | Household survey data obtained in 2015 from a stratified random sample in the city | 604 | Household medical expenses (oop, out-of-pocket expenditure portion) as a percentage of household affordability more than 40% | 4 |
| XX2014(62) | Guangdong Province, China | Cross-sectional | In 2012, 300 patients with stroke were diagnosed at the Hospital of the Stroke Screening and Prevention Base in northern Guangdong Province, where the hukou is located in Shaoguan City | 300 | Household out-of-pocket payments for health care (OOP) account for more than 40% of household expenditure | 5 |
| LH2015(63) | Hami City, Xinjiang, China | Cross-sectional | 2013 Hami District Residents' Family Health Inquiry Survey | 1845 | The ratio of household health expenditure to household consumption expenditure exceeds 40% | 5 |
| FWQ2014(64) | Rural areas of Heilongjiang Province, China | Cross-sectional | Rural families surveyed by the Fourth Health Service survey in Heilongjiang Province in 2008 | 3098 | Health expenditure accounts for more than 40% of household consumption expenditure | 3 |
| CLN2014(65) | Zhejiang, Hubei and Chongqing, China | Cross-sectional | An on-site investigation in Chongqing, Hubei Province, Zhejiang Province, China | 1661 | Household out-of-pocket health care costs account for more than 40% of household non-food consumer spending | 5 |
| YJ2014(66) | Rural Lushan County, Dalizhou, Yunnan Province, China | Cross-sectional | Rural families in Lushan County, Dalizhou | 1140 | 40 percent of household health financing contribution (household health expenditure/household affordable household > 40 percent | 4 |
| SBL2014(67) | Jinan, Shandong Province, China | Cross-sectional | Survey data on the demand and utilization of health services for rural residents in Jinan in 2012 | 2180 | Family members' out-of-pocket and health expenditures account for more than 40% of household consumption | 4 |
| ZQH2014(68 | Heilongjiang Province, China | Cross-sectional | Families who suffered catastrophic health expenditures and received insurance compensation in rural Heilongjiang Province in 2007 | 296 | 40 percent is used as a criterion for the classification of catastrophic health expenditure, based on the standard of family expenditure representing family life | 3 |
| WLD2013(69) | New Agricultural Co-pilot County, Anhui Province, China | Cross-sectional | A random sample of six pilot counties in Xinnong, Anhui Province, was randomly selected according to the economic level | 3149 | Personal out-of-pocket health expenditure accounts for more than 40% of household consumption | 4 |
| QJM2013(70) | Eight cities in China | Cross-sectional | A random sample of six pilot counties in Xinnong, Anhui Province, was randomly selected according to the economic level | 4915 | Household out-of-pocket payments account for 40% of non-food expenses | 5 |
| YJE2012(71) | Mei County, Baoji City, Shaanxi Province | Cross-sectional | A sample household survey of urban residents in Mei County, Shaanxi Province | 876 | Households pay more than 40 percent of household disposable income for health care in out-of-pocket | 3 |
| CJ2012(72) | Shanghai, China | Cross-sectional | In 2008, the first treatment of active tuberculosis patients from the non-resident population was newly registered and completed in Putuo District | 97 | Household health expenditure exceeds 40% of household non-food expenditure | 4 |
| LY2012(73) | Rural China | Cross-sectional | The Fourth National Health Service Survey of Rural Families | 38945 | Household out-of-pocket hygiene expenditure exceeds 40% of household non-food expenditure | 4 |
| JQC2012(74) | Rural areas of Anhui Province, China | Cross-sectional | In 2009, Anhui Province stratified random group sampling survey | 3149 | Family members' out-of-pocket hygiene expenditure accounts for more than 40% of household non-food consumption expenditure | 5 |
| WYY2011(75) | Rural areas of Sichuan Province, China | Cross-sectional | Family health was investigated in the Fourth Health Service Survey of Sichuan Province in 2008  Ask about the survey of rural low-income families in the western expansion date | 1980 | Household out-of-pocket hygiene expenditure accounts for 40% of household non-food expenditure | 5 |
| CY2011(76) | Ledu County, Qinghai Province, Gansu Province, and Zheng County, Shanxi Province, Tocheng County, and Ping lu County | Cross-sectional | The on-site investigation of the demonstration village of hypertension system management has confirmed the patients with primary hypertension and stroke, and coronary heart disease who township hospitals have diagnosed and above medical institutions | 1189 | Household health expenditure in out-of-pocket accounts for more than 40% of household income | 5 |
| CRY2012(77) | Tengzhou City, Shandong Province, China | Cross-sectional | A sample survey was conducted in 2 towns in Tengzhou City, Shandong Province | 179 | Household out-of-pocket hygiene expenditure accounts for more than 40% of household non-food expenditure | 5 |
| ZGA2018(78) | China | Cross-sectional | 2014 CFPS survey data | 13602 | Household out-of-pocket accounts for 40% or more of household non-food expenses | 5 |
| CJY2018(79) | Ezhou City, Hubei Province, China | Cross-sectional | Ezhou City 2016 "due to disease poverty, due to illness back to poverty" population data | 20961 | Household out-of-pocket accounts for 40% or more of household non-food expenses | 4 |
| CZY2016(80) | China | Cross-sectional | CHNS database collected jointly by the University of North Carolina's Carolina Population Center and the China Centers for Disease Control and Prevention | 4120 | Higher-income households: Health expenditure accounts for more than 25% of household per capita income | 4 |
| DHB2018(81) | Hebei Province, China | Cross-sectional | Field investigation field interview in Hebei Province | 1083 | Household out-of-pocket accounts for 40% or more of household non-food expenses | 3 |
| GGY2017(82) | China | Cross-sectional | A sample survey of the whole group of patients with critical illnesses in Beijing | 497 | Out-of-pocket health expenditure accounts for 40% of household consumption | 3 |
| GN2013(83) | Zhangqiu City, Changqing District, Pinyin County, Shandong Province, China | Cross-sectional | A stratified random sampling survey | 263 | Household out-of-pocket hygiene expenditure accounts for more than 40% of household non-food expenditure | 4 |
| GMT2014(84) | Heilongjiang Province, China | Cross-sectional | Data from Heilongjiang Province in the Fourth National Health Service Survey | 5661 | When the total household expenditure is equal to or greater than the basic living expenses, and the entire household expenditure excluding out-of-pocket health expenditure is less than the basic living expenses | 5 |
| HL2006(85) | Shanxi Province, China | Cross-sectional | In 2004, 607 peasant families in poor counties in Shanxi Province conducted a quantitative survey on the financing and willingness to participate in new rural cooperative medical care | 607 | Household out-of-pocket payments account for more than 40% of household consumption | 5 |
| HXJ2013(86) | China | Cross-sectional | CHNS-related data for 2009 | 2931 | Household out-of-pocket accounts for more than 40% of household consumption expenditure | 4 |
| JXR2006(87) | Shanghai, China | Cross-sectional | A sample survey of the new rural cooperative medical operation mode in the suburbs of Shanghai | 3494 | Household out-of-pocket accounts for more than 40% of household consumption expenditure | 3 |
| JX2015(88) | Yunnan Province, China | Cross-sectional | A sample survey of AIDS patients in Dalizhou, Yunnan Province | 461 | The average net income of households is the sum of the total net income of households/the total number of respondents, and the maximum capacity to pay is the net income of 1/2 households | 6 |
| CM2010(89) | Ningbo, China | Cross-sectional | Ningbo Cixi, Fenghua rural residents medical institutions file records | 1215300 | Paying for medical care exceeds a specific value to bring the standard of living below the poverty line | 5 |
| DY2017(90) | Fuzhou, China | Cross-sectional | China Health Statistics Yearbook, Fuzhou City Statistical Yearbook, Fuzhou City Health and Family Planning Commission's new agricultural joint management department records, Fuzhou City participating in the rural residents hospitalized patients or their families questionnaire | 1191 | A percentage of residents' out-of-pocket and health expenditure exceeds total household consumption expenditure (40%) | 6 |
| HJS2018(91) | Fuzhou, China | Cross-sectional | Monitoring data from the State Forestry Administration's "Reform Monitoring of Key State-owned Forest Areas" project in 2016 | 790 | Direct household medical expenses account for more than 25% of household non-food expenses | 5 |
| LH2015(92) | Hami City, Xinjiang, China | cross sectional | Data from the 2013 Health Services Survey in Hami, Xinjiang | 1845 | The ratio of household health expenditure to household consumption expenditure exceeds 40% | 5 |
| LvH2012(93) | Zhejiang, Hubei and Chongqing, China | Cross-sectional | Sample survey in Zhejiang, Hubei, and Chongqing, China, 2011 | 1697 | Out-of-pocket health expenditure as a percentage of income exceeds 30% | 5 |
| LHQ2016(94) | Hubei Province, China | Cross-sectional | Hubei Province A land household survey, new agricultural joint agency records | 441 | Health spending for people with significant illnesses accounts for more than 40% of household affordability | 4 |
| NYX2017(95) | Shandong Province, China | Cross-sectional | Two urban communities, Fushan District of Yantai City, Weifang City, Weicheng District, and four rural counties, Luyuan County, Liangshan County, Dairy Mountain City, Gaotang County, conducted a questionnaire survey | 2183 | Families with diabetes pay more than 40% of household non-food expenses | 5 |
| WHP2016(96) | Nine provinces and cities in China | Cross-sectional | The poverty alleviation task force investigated poor villages in poor counties | 1214 | Household out-of-pocket medical expenses exceed non-food expenses by 40% | 3 |
| WJJ2012(97) | Shijiazhuang, Hebei Province, China | Cross-sectional | Shijiazhuang City, urban households into the household survey | 305 | Household out-of-pocket medical expenses exceed non-food expenses by 40% | 5 |
| WHY2016(98) | China | Cross-sectional | Data on rural residents in the CHNS survey for 2011 | 787 | Health expenditure accounts for more than 10% of household income | 5 |
| SXZ2007(99) | Shandong Province, China | Cross-sectional | A sample survey of whole stratified groups in the pilot county of Xinnong joint in Shandong Province | 375 | 40.00% of household health expenditure exceeds the capacity to pay | 3 |
| WQC2016(100) | China | Cross-sectional | The 2014 China Household Tracking Survey surveyed rural households | 17587 | Health care accounts for 40% of household non-food spending | 6 |
| LJ2017(101) | Shanxi Province, China | Cross-sectional | A sample survey of households in Taiyuan City | 455 | Out-of-pocket medical expenses account for more than 40% of household non-food expenses | 5 |
| LXH2014(102) | Jiangsu Province, China | Cross-sectional | A stratified random sampling questionnaire in Jiangsu Province | 1960 | Out-of-pocket medical expenses account for more than 40% of household non-food expenses | 5 |
| SZJ2010(103) | Huaihua City, China | Cross-sectional | A random sample survey of Huaihua City | 320 | Out-of-pocket medical expenses account for more than 40% of household non-food expenses | 4 |
| XNZ2016(104) | Chongqing, China | Cross-sectional | The fifth NHSS Chongqing rural areas of hypertension population survey | 4244 | Out-of-pocket medical expenses for family members exceed 40% of household disposable income | 5 |
| YF2013(105) | Hebei, Henan and Yunnan, China | Cross-sectional | Families with maternal deaths in rural areas of Yunnan, Henan Province, Hebei Province | 195 | The disease costs more than 40% of household non-food expenses | 3 |
| WQC22016(106) | China | Cross-sectional | Data from the 2014 China Household Tracking Survey | 30062 | Household out-of-pocket payments for medical expenses account for more than 40% of household non-food consumption expenditure | 4 |
| WL2014(107) | Zunyi City, China | Cross-sectional | A random sample site survey in Zunyi City, Guizhou, in 2010 | 615 | The standard of living of families for paying for medical care is below the poverty line | 3 |
| ZYC2007(108) | Huairou District, Beijing, China | Cross-sectional | New agricultural statistics for Huairou District | 33411 | Health expenditure accounts for 40% of household income | 3 |
| XYL2011(109) | Guangdong Province, China | Cross-sectional | Guangdong Province, the new rural cooperative medical sustainable development research and analysis team in 2008 household survey | 209 | Residents earn less than the local minimum living security line after paying for medical care | 3 |
| YJ2017(110) | Tongchuan, China | Cross-sectional | A questionnaire survey of farmers in Indo-Tai District, Tongchuan City | 250 | The standard of living of the population is lower than the local poverty line because of the cost of health | 5 |
| SBT2013(111) | Yulong County, Yunnan Province, China | Cross-sectional | Yulong County sample survey | 300 | Households have paid more than 40% of their total medical expenses in the past year | 4 |
| ZML2016(112) | China | Cross-sectional | CHARLS 2013 survey data | 1387 | Health care costs paid in out-of-pocket account for 40 percent of household non-food expenditure | 6 |
| WXL2016(113) | Nanchang, China | Cross-sectional | A questionnaire survey of lung cancer patients in Nanchang | 203 | Household health care costs account for more than 40% of household non-food expenses | 6 |
| WXJ2018(114) | Guangxi Zhuang Autonomous Region, China | Cross-sectional | A hospital affiliated with a university in Guangxi was hospitalized for myocardial infarction in 2016-2017 | 500 | Out-of-pocket medical expenses account for more than 40% of household non-food expenditure throughout the year | 5 |
| Che Y2016(115) | Yunnan Province, China | Cross-sectional | Patients with no complications of chronic HBV over 20 years of age in Yunnan Province, patients with reparational HBV cirrhosis, non-compensation HBV cirrhosis, and hepatocellular carcinoma | 940 | Total household health expenditure exceeds 40 percent of household capacity to pay | 6 |
| Li, Y2012(116) | China | Cross-sectional | Fourth National Health Service Survey (NHSS) 2008 | 55556 | Household out-of-pocket medical expenses exceed 40% of household non-survival expenses | 5 |
| Li, X2013(117) | Shanghai, Changzhou, Weifang, China | Cross-sectional | 2008 Shanghai, Changzhou, Weifang City residents health utilization and cost survey | 11577 | Household out-of-pocket medical expenses account for more than 40% of household non-survival expenses | 6 |
| Zhang, W2015(118) | China | Cross-sectional | 2011-2012 China Health and Pensions Tracking Survey of families aged 60 and over | 2700 | Medical expenses account for 40 percent or more of households' ability to pay | 4 |
| Zhou, C2016(119) | Jiangsu Province, Hubei Province, Shaanxi Province, China | Cross-sectional | TB patients diagnosed in Yichang, Han Zhong, and Zhenjiang in 2012 | 347 | Household out-of-pocket medical expenses exceed 40% of non-food expenses | 6 |
| Zheng, A2018(120) | Liaoning Province, China | Cross-sectional | A multi-stage stratified random sample survey of residents of 6 types of cancer patients in Liaoning Province | 1344 | Household out-of-pocket medical expenses account for 40 percent of household non-survival expenses | 6 |
| Gwatidzo, S. D2017(121) | China | Cross-sectional | Global Aging and Adult Health Study 2007-2010 study data for people aged 50 and over-diagnosed with non-gestational diabetes questionnaire | 630 | Out-of-pocket medical expenses account for more than 40% of household non-food expenses | 5 |
| Yang, T2016(122) | Shandong Province, China | Cross-sectional | A stratified random sample survey of elderly families in Shandong Province | 2761 | Out-of-pocket medical expenses exceed 40% of the family's ability to pay | 5 |
| Mao, W2017(123) | Shanghai, Beijing, Fuzhou, Chongqing, China | Cross-sectional | A simple random sample of cancer patients in Shanghai, Beijing, Fuzhou, and Chongqing who were reimbursed using urban job insurance | 2408 | Household health expenditure > average size of households (per capita consumption expenditure - per capita food expenditure) | 6 |
| Wang, Q2014(124) | China | Cross-sectional | "Community Health Promotion in Rural Poor Areas of China" Hong Kong Kado program 2009 survey data of 21-90-year-old with hypertension and its complications | 947 | Out-of-pocket costs exceed 40% of household affordability | 6 |
| Sun, X2016(125) | China | Cross-sectional | A simple random sample of cancer patients in Shanghai, Beijing, Fuzhou, and Chongqing who were reimbursed using urban job insurance | 5511 | Oral health expenditure accounts for 20% of the annual household income | 5 |
| CMS2016(126) | Minority areas in western China, Qinghai, Xinjiang, Tibet | Case-control | Data from the 2014 final survey on the integration of rural health services in ethnic minority areas in the west | 3066 | Household residents out-of-pocket health expenditure as a proportion of household consumption more than 40% | 6 |
| MY2015(127) | Chenyi County, Xianyang City, Shaanxi Province, China | Case-control | In 2013, the status survey was conducted on the critical illnesses in The County and the patients with critical diseases provided by the New Agricultural Joint Economic Cooperation Center in the county | 314 | Household out-of-pocket health expenses account for more than 40% of household consumption expenditure | 4 |
| JRF2014(128) | Sangzhi County, Mayo County, and Blue Mountain County, Hunan Province, China | Case-control | Random group sampling and systematic sampling methods were used to extract families from Sangzhi County, Mayo County, and Blue Mountain County in Hunan Province | 400 | The ratio of household health expenditure to household consumption expenditure exceeds 40% | 6 |
| YJE2013(129) | Mei County, Baoji City, Shaanxi Province, China | Case-control | A sample survey of Mei County, Shaanxi Province, in 2011 | 1747 | Household out-of-pocket payments account for more than 40% of household non-food expenses | 4 |
| CRY2012(130) | Tengzhou City, Shandong Province, China | Case-control | A stratified random sample survey in Tengzhou City, Shandong Province | 179 | Health expenditure accounts for 40% of household non-food expenditure | 5 |
| YRL2012(131) | Mei County, Shaanxi Province, China | Case-control | Shaanxi Province eyebrows  County household survey | 1468 | Households pay more than 40% of non-food expenses in out-of-pocket | 4 |
| QDF2016(132) | Inner Mongolia, Qinghai, China | Case-control | Survey of patients with hypertension in the pilot area | 1731 | Residents' out-of-pocket health expenditure of medical expenses accounted for more than 40% of household consumption expenditure | 6 |
| FWJ2014(133) | Hangzhou, China | Case-control | Household survey of elderly people over 60 years of age in Hangzhou | 530 | More than 40 percent of household non-food expenditures for older persons | 6 |
| JYH2017(134) | Heilongjiang Province, China | Case-control | Data from Heilongjiang Province, the fifth national health service survey, 2013 | 6601 | More than 40 percent of household non-food expenditures for older persons | 4 |
| LC2016(135) | Hubei Province, China | Case- control | The household entry survey and the new agricultural joint system records in B City, Guizhou Province | 872 | Household out-of-pocket health care costs account for more than 40% of household non-food consumer spending | 5 |
| LD2005(136) | Shanxi Province, China | Case-control | "Rural Family Health Inquiry Into Household Baseline Survey Data" | 570 | Household out-of-pocket payments account for 40% of household non-food expenses | 5 |
| SJ2017(137) | Hubei Province, China | Case-control | A random sample survey of rural areas in M City, Hubei Province | 536 | Households pay 40 percent of household income for out-of-pocket medical care and 40 percent for non-survival expenses | 6 |
| SXJ2008(138) | Xining, Yinchuan, China | Case-control | Data from the 2006 UHPP Project Xining and Yinchuan City Household Survey | 1567 | Households' monthly health expenditure accounts for more than 40% of monthly consumption expenditure (excluding food expenditure) | 6 |
| XWJ2018(139) | China | Case-control | China Health and Pensions Tracking Survey 2015 | 9706 | Medical expenditure equals or exceeds 40% of disposable income after meeting basic survival expenses | 7 |
| HG2010(140) | Jiangxi Province, China | Case-control | In 2007, a questionnaire survey of low-security farmers who lived in Yuyuan County for more than half a year | 261 | Households pay 40 percent of household income for out-of-pocket medical care and 40 percent for non-survival expenses | 5 |
| FH2004(141) | China | Case-control | China Statistical Yearbook, Archives | 9700 | Total household health expenditure accounts for more than 40% of household affordability | 6 |
| HX2010(142) | Jiangxi Province, China | Case-control | A sample survey of Shushui, Luxi, and Yuyuan counties | 15458 | Household health spending exceeds 40% of household consumption expenditure | 6 |
| SD2016(143) | Xiangtan City, Hunan Province, China | Case- control | "Urban low-income families economic and health survey" task force in 2013 Xiangtan City household survey | 1165 | Household health spending exceeds 40% of household consumption expenditure | 6 |
| ZYL2008(144) | Shanghai, Nanning, | Case-control | Information on inpatients at Sanjia Hospital in Shanghai and Nanning City in 2007 | 928 | Medical expenses paid in out-of-pocket account for 40% of the family's annual income | 4 |
| ZWG2006(145) | Dongying City, China | Case-control | Investigation on the health status and factors of rural residents in Dongying City | 579 | More than 40% of household consumption is spent on health care in out-of-pocket | 6 |
| ZYX2004(146) | Heilongjiang Province, Gansu Province, China | Case-control | Survey of households in rural areas of Gansu and Heilongjiang provinces in 2002 | 3890 | Household out-of-pocket accounts for more than 40% of household expenses | 5 |
| YXL2013(147) | A province of China | Case-control | S Province 2008 Household Health Services Survey | 3958 | Out-of-pocket hygiene expenditure accounts for more than 40% of household non-food expenditure | 5 |
| YY2014(148) | Dali Bai Autonomous Prefecture, Yunnan Province, China | Case-control | Residents of Dali City, Midu County, and Lushan County, Yunnan Province, conducted on-site investigations | 3697 | Household health expenditure accounts for more than 40 percent of household affordability | 6 |
| HHY2018(149) | Dali Bai Autonomous Prefecture, Yunnan Province, China | Case-control | A stratified random sample survey was conducted on patients hospitalized with late blood assistance in Jiangsu Province from January 2012 to December 2014 | 2626 | Personal out-of-pocket medical expenses account for 40% of the family's annual net income per capita | 7 |
| QCG2015(150) | Gansu Province, China | Case-control | Data from Gansu Province, the fifth National Health Service Survey, 2013 | 7500 | Out-of-pocket payments for medical expenses account for more than 29% of household non-food expenses | 6 |
| Chen J2018(151) | Zhejiang Province, China | Case-control | A random sample survey of rural residents in Yuyao and Fenghua, Zhejiang Province | 4900 | Household out-of-home medical expenses as a percentage of household net income in the absence of third-party compensation | 7 |
| Xu, Y2018(152) | Shaanxi Province, China | Case-control | Data on chronic families surveyed by the National Health Service in Shaanxi Province in 2008 and 2013 | 9646 | Household out-of-pocket pay more than 40 percent of household non-subsistence expenses | 6 |
| Zhang, L2010(153) | Yuexi County, Submarine County, Datong County, China | Case-control | In 2006, a random sample survey of inpatients in Yuexi County, Submarine County, and Datong County was conducted | 881 | Household out-of-pocket health care costs account for more than 10% of total annual household expenditure | 7 |
| Zhen, X2018(154) | Zhejiang, Qinghai, China | Case-control | Qinghai Family Survey 2016 | 1598 | Out-of-pocket medical expenses exceed 40% of household disposable income | 5 |
| Liu, X2016(155) | Pingyin, Liangshan, Jun'an, Shandong, China | Case- control | In June 2011, a random sample survey of patients with hypertension and diabetes in Shandong Province was conducted | 1635 | Residential medical expenses exceed 40% of household non-food expenses | 8 |
| Wang, J2014(156) | Hubei, Chongqing, Zhejiang, China | Case-control | A sample survey of rural residents in Hubei, Chongqing, and Zhejiang | 1525 | Family out-of-pocket medical expenses exceed 40% of total household income | 6 |
| Sun, J2015(157) | Inner Mongolia, China | Case-control | Family questionnaires with members of cardiovascular disease in rural areas of Inner Mongolia | 949 | Household out-of-pocket medical expenses exceed 40% of household non-self-searching expenses | 7 |
| YHY2012(158) | 25 provinces and autonomous regions of China | Cohort | China Family Dynamic Tracking Survey | 47082 | Household out-of-pocket health expenditure exceeds 40% of household income or expenditure for a certain period | 5 |
| CJH2006(159) | Province, Ningxia Province, China | Cohort | Baseline on-site research and year-end on-site research data for the project "Building a Fair and Sustainable Health Security System in China and Vietnam." | 9415 | Household out-of-pocket health expenditure exceeds 40% of household income or expenditure for a certain period | 5 |
| Gu2009(160) | City, Jiangsu Province, China | Cohort | A random sample survey of a hierarchical group in Xuzhou City | 3220 | Household out-of-pocket medical expenses account for more than 40% of household non-survival expenses | 6 |
| Jing2006(161) | Province, Ningxia Hui Autonomous Region, China | Cohort | Household surveys of chronically ill patients were conducted in Shandong and Ningxia in 2006 and 2008 | 4685 | Health expenditure accounts for 40% of household non-food expenditure | 5 |
| Li2011(162) | China | Cohort | CHARLS surveyed data on patients with chronic and cardiovascular diseases in 2011 and 2013 | 5624 | Annual household health expenditure accounts for 40% and more of household non-food expenditure | 5 |

45. Huang J, Cai L, Li X, Cui W, Wang G, Lv S. Current situation of diabetes incidence and disease economic risk in rural elderly people in Midu County, Yunnan Province. Chinese Journal of Public Health (2019) 35(11):1461-4. doi: 10.11847/zgggws1118984.

46. Sun Y, Yan Y, Xue Q, Gao J. Catastrophic health expenditure and its inequality breakdown among diabetics in Shaanxi Province. Chinese Journal of Public Health. (2018) 34(8):1143-7. doi: 10.11847/zgggws1116888.

47. Huang S, Yin A. Catastrophic health expenditure of rural households in Shandong Province and its influencing factors. Chinese Journal of Public Health. (2018): 34(9):1221-3. doi: 10.11847/zgggws1116290.

48. Li X, Song H. Study on the economic risk of diseases in middle-aged and elderly population in Jiangsu Province. Medical Higher Vocational Education and Modern Nursing. (2018) 1(2):112-4. doi: 10.3969/j.issn.2096?510X.2018.02.016.

49. Lu X, Ci Q. Influencing factors of catastrophic health expenditure of poor households and medical assistance policy options. Guangxi Social Sciences. (2017) (266):152-7.

50. Li F, Wang C, Gan Y, Zhang X, Lin J, Cai D. Analysis of medical assistance in Shanghai in 2015. China Health Economics. (2017) 36(11):29-31. doi: 10.7664/CHE20171108.

51. Qing L, Le C, Wen l, Ding Y, Wen l, Yue L. Prevalence of four chronic diseases in rural residents of Yunnan Province and their impact on household economy. Chinese Journal of Public Health. (2018) 34(4):479-82. doi: 10.11847/zgggws1113224.

52. Huang X, Xiang G, Li T, Gu X. Research on the effect of medical security on reducing the economic risk of chronic diseases in poor urban families. Health Economics Research. (2017) (8):51-3.

53. Li X, Tang R. Current situation and influencing factors of catastrophic health expenditure of rural households in Yinchuan City. J J Primary Health Care. (2017) 31(7):1-3. doi: 10.3969/j.issn.1001-568X.2017.07.0001.

54. Wang Y, Qiu P, Tian F, Liu H, Zhang Q. Current situation and influencing factors of catastrophic health expenditure of rural households in Fushun County, Sichuan Province. Chinese Journal of Public Health. (2019) 35(2):152-6. doi: 10.11847/zgggws1116874.

55. Li Y, Li C, Yan M. Study on the influence of serious illness insurance on the burden of medical expenses of urban and rural residents. Chinese Journal of Social Medicine. (2017) 34(5):501-3. doi: 10.3969/j.issn.1673-5625.2017.05.025.

56. Gao M, Yang J, Yan H, Li S. Catastrophic health expenditure of rural households in Hubei Province and its influencing factors. China Health Statistics. (2016) 33(6):1008-9, 1013.

57. Peng M, Zeng L, Li J, Li J, Zhu C, Hu P. Analysis and enlightenment of the burden of medical treatment in tertiary hospitals of participating farmers: A case study of Zhongjiang County, Sichuan Province. Medicine and Philosophy (A). (2017) 38(04):47-50.

58. Wang H, Zhao Y, Ding T, Cao Z. Discussion on the Definition Standards of Rural Household Catastrophic Health Expenditure: A Case Study of Qianxi County, Hebei Province. Economist. (2016) (11):132-3.

59. Zhou Y, Tang Y, Zhang Q, Qiu P, Zhang Q. Study on the catastrophic health expenditure of rural households in Zigong City, Sichuan Province before and after the new rural cooperative medical compensation and its influencing factors. JOURNAL OF SICHUAN UNIVERSITY (HEALTH SCIENCES EDITION). (2016) 47(05):768-71.

60. Wang Z, Li X. Analysis of economic risks and influencing factors of rural elderly families with chronic diseases. Medicine and Philosophy. (2016) 37(13):55-7, 60. doi: 10.12014/j.issn.1002-0772.2016.07a.14.

61. Feng Z, Li D, Chen Y, Xiong J, Wang J, Li P. Analysis of household catastrophic health expenditure and its influencing factors in a city in western Hubei Province. Medicine and Society. (2017) 30(4):54-7. doi: 10.13723/j.yxysh.2017.04.016.

62. Xu X, Liu J, Fan S, Zhao Y. Analysis of catastrophic health expenditure of stroke patients in northern Guangdong and its influencing factors. Soft Science for Health. (2014) (8):491-3, 494. doi: 10.3969/j.issn.1003-2800.2014.08.001.

63. Li H. Analysis of economic risk of urban household disease and catastrophic health expenditure and influencing factors in Hami area. China Health Economics. (2015) 34(7).

64. Fu W, Li B, Zhang Q, Liu G. Analysis of catastrophic health expenditure of rural households in Heilongjiang Province. China Health Economics. (2014) (2):46-7. doi: 10.7664/CHE20140214.

65. Chen L, Wei W, Wang J, Zhang L. A Study on the Catastrophic Health Expenditure of Poor and Low-income Residents in New Rural Cooperatives: Based on a Sample Survey of Three Provinces. China Health Policy Research. (2014) 7(04):32-7.

66. Yin J, Zhao H, Wang Y, Yang Z, Huang B, Huang F, et al. Influencing factors of catastrophic health expenditure in rural households in Weishan County, Dali Prefecture. Soft Science for Health. (2014) 28(4):205-7.

67. Su B Shi C. A study on access to health services and equity in health care burden for low-income farmers. Soft Science for Health. (2014) 28(5):272-5.

68. Fu W, Zhang Q, Li B, Liu G. An empirical study on the degree of social medical insurance economic security in important target populations. China Health Economics. (2014) 33(02):41-2.

69. Wang L, Wang A, Wu N, Fang G, Si L, Jiang Q. Analysis of the impact of cash health expenditure on poverty among vulnerable rural population in Anhui Province and its related factors. China Health Economics. (2013) 32(5):69-71. doi: 10.7664/CHE20130522.

70. Qin J. Comparative Analysis of the Occurrence of Catastrophic Health Expenditure: A Health Inquiry Survey of Typical Urban Residents Based on Comprehensive Community Health Reform. Community Health Services. (2013) 32(9):65-8.

71. Yan J. A study on the poverty and disastrous impact of cash health expenditure of urban residents in Shaanxi Province. China Health Economics. (2012) 31(8):25-8.

72. Chen J, Zheng Y, DENG Haiju, LIU Yan. Analysis of household catastrophic health expenditure of new-onset tuberculosis patients in non-registered population. Shanghai Prev M. (2012) 24(11):590-2.

73. Li Y, Wu Q, Gao L. Analysis of the institutional causes of catastrophic health expenditure of rural residents in China. China Health Policy Research. (2012) 5(11):55-9. doi: 10.3969/j.issn.1674-2982.2012.11.009

74. Wang L, Jiang Q, Wang A, Wu N, Fang G, Si L. Analysis of catastrophic health expenditure of rural residents in Anhui Province. China Health Policy Research. (2012) 05(4):59-62. doi: 10.3969/j.issn.1674-2982.2012.04.011.

75. Wan Y, Luo M, Lin Y, Zhang J, Ying G, Gan H, et al. Analysis of influencing factors of catastrophic health expenditure of low-income households in rural Sichuan. Modern Preventive Medicine. (2011) 38(23):4889-91.

76. Cui Ying, Liu Jun'an, Ye Jianli, Wang Qi, Yang Li, Shi Shuhua, et al. Analysis of catastrophic health expenditure of households with hypertension and its comorbidities in poor rural areas. Primary health care in China. (2011) 25(3):37-9. doi: 10.3969/j.issn.1001-568X.2011.03.016.

77. Chen R, Yin A, Zhao W, Han Z, Wang W, Xu L, et al. Influencing factors of catastrophic health expenditure of rural residents in Tengzhou City. China Health Economics. (2012) 31(3):19-21. doi: 10.3969/j.issn.1003-0743.2012.03.006.

78. Zhang Y, Zhai T, Wei Q, Zhao Y. A case study on the relationship between the proportion of personal health expenditure and the economic burden of medical care of residents. Health Economics Research. (2011) (6):18-21.] doi: 10.3969/j.issn.1004-7778.2011.06.008.

79. Chen J, Qin Y, Xiao W, Xiong W. Analysis of the causes of poverty caused by disease in Ezhou City. Practical Preventive Medicine. (2018) 25(8):958-61. doi: 10.3969/j.issn.1006-3110.2018.08.016.

80. Chen Z, Jiang Y, Li W. The Impact of New Rural Cooperation on Rural Residents' Disastrous Medical Expenditure: Based on the Background Analysis of Universal Coverage. Journal of Finance and Economics. (2016) (12):110-20.

81. Wu Z and Duan H. A Study on the Poverty of the Elderly Population in Hebei Province: Based on a Field Survey of 14 Counties in Hebei Province. Journal of Hebei University. Philosophy and Social Sciences. (2018) 43(1):112-8.

82. Gao G, Ma C, Hu X, Yang X, Duan T, Jia J. Evaluation of the effect of the new rural cooperative serious illness insurance system on alleviating catastrophic health expenditure. Social Security Research. (2017) (02):69-76.

83. Guo N, Zhu D, Tor I, Wang J. Impact and equity of new agricultural cooperation on catastrophic health expenditures. Chinese Journal of Public Health. (2013) 29(11):1584-7.

84. Guo M, Wu Q, Li Y, Huang Z, Gao L, Hao Y, et al. Analysis of hospitalization expense compensation, out-of-pocket ratio and poverty due to illness of medical insurance patients. Chinese Hospital Management. (2014) 34(12):74-6.

85. He L, Zheng J, Han Y. Analysis of financing fairness of new rural cooperative medical families in Shanxi Province. China Rural Health Management. (2006) 26(11):3-6. doi: 10.3969/j.issn.1005-5916.2006.11.001.

86. He X, Chen Z, Ma A. Analysis of demographic characteristics of poverty caused by illness of farmers in China. Modern Commerce and Trade Industry. (2013) (9):58-60. doi: 10.3969/j.issn.1672-3198.2013.09.029.

87. Jia X, Chen X, Zhang W, Shao H. Research on the ability of new rural cooperative medical care in the suburbs of Shanghai to prevent poverty caused by illness. Journal of Health Economics. (2006) (1):34-5. doi: 10.3969/j.issn.1004-7778.2006.01.017.

88. Ji X, Huang L, Wan J, Yang G, Xu P, Wang Y. Analysis of HIV-infected patients and patients in areas with high incidence of HIV/AIDS. Medicine and Society. (2015) 28(03):33-6.

89. Chen. Research on the financing level of new rural cooperative medical goals [Master].: Ningbo University (2010).

90. Deng Y. Research on the benefits and influencing factors of farmers participating in the new rural cooperative medical treatment in Fuzhou City [Master].: Fujian Medical University (2017).

91. Hou J. Research on catastrophic health expenditure of workers in state-owned forest area of Greater Xing'anling and its influencing factors [Master].: Heilongjiang University of Traditional Chinese Medicine (2018).

92. Li H. Research on Cash Health Expenditure and Catastrophic Health Expenditure of Residents in Eastern Xinjiang [Master].: Shihezi University (2015).

93. Lv H. Research on medical security system for rural poor based on disease economic risk [Ph.D.].: Huazhong University of Science and Technology (2012).

94. Luo H. Analysis of Compensation Model and Implementation Effect of Serious Illness Insurance for Rural Residents Based on UHC Perspective [Master].: Huazhong University of Science and Technology (2016).

95. Niu Y. Current situation and influencing factors of catastrophic health expenditure of diabetic patients in urban and rural residents in Shandong Province [Master].: Shandong University (2017).

96. Wang H, Wang Z, Ma P. Analysis and Reflection on Poverty Caused by Illness in Rural Areas: Based on the survey data of 1214 households in 9 provinces and cities in western China. The Economist. (2016) (10):71-81.

97. Wang J. Research on the Current Situation of Urban Household Catastrophic Health Expenditure and Its Medical Security System: A Case Study of Shijiazhuang City [Master].: Hebei University of Economics and Business (2012).

98. Wang H. Research on the economic risk of disease of rural residents and the compensation effect of the new agricultural cooperation [Master].: Central China Normal University (2016).

99. X Sun, Adrian S, S Li, Gordon C, Sukhan J. Evaluation of the effect of the new rural cooperative medical care in protecting farmers from disease economic risks. China Health Economics. (2007) (02):14-7.

100. Wang Q. The Impact of Catastrophic Medical Expenditure on Rural Poverty in China: Based on the Chinese Household Longitudinal Survey. China Health Policy Research. (2016) 9(02):6-10.

101. Li J. Research on catastrophic health expenditure and economic risk of disease of Taiyuan residents [Master].: Shanxi Medical University (2017).

102. Liu X. Research on catastrophic medical expenditure, rural household vulnerability and medical security [Master].: Nanjing Agricultural University (2014).

103. Shen Z. Economic Risk Analysis of Disease of Farmers in Huaihua City [Master].: Hunan Agricultural University (2010).

104. Xiao N, Zhong X, Wang Y, Tang S. Economic risk of hypertension in rural Chongqing. Journal of Third Military Medical University. (2016) 38(23):2552-8. doi: 10.16016/j.1000-5404.201606036.

105. Ye F, Feng Y, Xu Y, Wang Y. Characteristics of poverty risk of rural maternal death households. China Health Economics. (2013) (12):24-6. doi: 10.7664/CHE20131206.

106. Wang Q. Financing needs to eliminate catastrophic health expenditure and their poverty reduction effects. Health Economics Research. (2016) (04):31-4.

107. Wei L, Tong X, Feng Y, Zhu X. Research on the ability of new rural cooperative medical care to solve poverty caused by illness in District H, Zunyi City. Special Economic Zone. (2014) (12):134-6.

108. Zhang Y. Problems and countermeasures of new rural cooperative medical care in China. Health Economics Research. (2007) (05):9-11.

109. Xu Y, Li Y, Wu L. Construction of the effect index of new rural cooperative medical treatment in alleviating poverty caused by illness. Journal of Finance and Economics. (2011) (10):108-16.

110. Yu J. Factors and countermeasures of poverty caused by illness among rural residents [Master].: Shaanxi Normal University (2017).

111. Shi B. Current situation of new rural cooperative medical system in Yulong County, Yunnan Province and its impact on the medical burden of farmers' families [Master].: Kunming Medical University (2013).

112. Zhao M. Research on the influencing factors of medical expenses of urban and rural residents [Master].: Shandong University (2016).

113. Wu X. Research on the Economic Burden of Lung Cancer Patients in Nanchang and the Impact of Medical Insurance Policy [Master].: Nanchang University (2016).

114. Wu X. Analysis of the preventive effect of basic medical insurance on catastrophic medical expenditure in China: A case study of hospitalized myocardial infarction patients [Master].: Guilin Medical College (2018).

115. Che Y, Chongsuvivatwong V, Li L, Sriplung H, Wang YY, You J, et al. Financial burden on the families of patients with hepatitis B virus-related liver diseases and the role of public health insurance in Yunnan province of China. Public Health. (2016) 130:13-20. doi: 10.1016/j.puhe.2015.03.015.

116. Li Y, Wu Q, Xu L, Legge D, Hao Y, Gao L, et al. Factors affecting catastrophic health expenditure and impoverishment from medical expenses in China: policy implications of universal health insurance. B World Health Organ. (2012) 90(9):664-71. doi: 10.2471/BLT.12.102178.

117. Li X, Shen JJ, Lu J, Wang Y, Sun M, Li C, et al. Household catastrophic medical expenses in eastern China: determinants and policy implications. Bmc Health Serv Res. (2013) 13:506. doi: 10.1186/1472-6963-13-506. PubMed PMID: 24308317.

118. Zhang W, Li G. Study on the occurrence of catastrophic health support in elderly families and its influencing factors. Journal of Shanghai Jiao Tong University (Health Sciences). (2015) 35(3):432-6. doi: 11.3969/j.issn.1674-8115.2015.03.025.

119. Zhou C, Long Q, Chen J, Xiang L, Li Q, Tang S, et al. The effect of NCMS on catastrophic health expenditure and impoverishment from tuberculosis care in China. Int J Equity Health. (2016) 15(1). doi: 10.1186/s12939-016-0463-0.

120. Zheng A, Duan W, Zhang L, Bao X, Mao X, Luo Z, et al. How great is current curative expenditure and catastrophic health expenditure among patients with cancer in China? A research based on “System of Health Account 2011”. Cancer Med-Us. (2018) 7(8):4036-43. doi: 10.1002/cam4.1590.

121. Williams S. Diabetes mellitus medication use and catastrophic healthcare expenditure among adults aged 50+ years in China and India: results from the WHO study on global AGEing and adult health (SAGE). Bmc Gastroenterol. (2017) 17(14).

122. Yang T, Chu J, Zhou C, Medina A, Li C, Jiang S, et al. Catastrophic health expenditure: a comparative analysis of empty-nest and non-empty-nest households with seniors in Shandong, China. Bmj Open. (2016) 6(7): e10992. doi: 10.1136/bmjopen-2015-010992.

123. Mao W, Tang S, Zhu Y, Xie Z, Chen W. Financial burden of healthcare for cancer patients with social medical insurance: a multi-centered study in urban China. Int J Equity Health. (2017) 16(1). doi: 10.1186/s12939-017-0675-y.

124. Qi W. Role of the new rural cooperative medical system in alleviating catastrophic medical payments for hypertension, stroke and coronary heart disease in poor rural areas of China. Bmc Public Health. (2014) 907(14).

125. Sun X, Bernabé E, Liu X, Gallagher JE, Zheng S. Determinants of Catastrophic Dental Health Expenditure in China. Plos One. (2016) 11(12): e168341. doi: 10.1371/journal.pone.0168341.

126. Chen M. A Study on Catastrophic Health Expenditure of Residents in Ethnic Minority Areas in Western China: A Comparison Based on the Implementation of the Integrated Model of Rural Health Services. China Health Policy Research. (2016) 9(10):25-30.

127. Mao Y, Zhu B, Liu J, Wu J, Jing P, Li Y, et al. Evaluation of serious illness insurance policy in China: An empirical study based on Xunyi County. China Health Economics. (2015) 34(08):10-4.

128. Jing R, Wang X, Cui Y, Feng R, Feng J, Liu S, et al. The impact of new rural cooperative medical care on the catastrophic health expenditure of rural residents in three counties of Hunan Province. China Health Economics. (2014) (2):48-50. doi: 10.7664/CHE20140215.

129. Yan J, Hao N, Liao S, Li Y, Shi F. Changes and influencing factors of disastrous health expenditure of rural households before and after the new medical reform: Based on a sample survey in Meixian County, Shaanxi Province. Health Policy Research in China. (2013) 6(02):30-3.

130. Chen R, Yin A, Zhao W, Han Z, Wang W, Xu L, et al. Association between economic risk of disease and catastrophic health expenditure of rural residents. Health Economics Research. (2012) (3):26-9. doi: 10.3969/j.issn.1004-7778.2012.03.008.

131. Yan J, Yan Y, Hao N, Yang J, Gao J, Li Q, et al. An empirical study on the effect of three basic medical security systems on improving catastrophic health expenditure. China Health Economics. (2012) 31(01):26-8.

132. Chen M, Qian D, Feng Z. A study on catastrophic health expenditure of hypertensive patients in ethnic minority areas in western China. China Health Economics. (2016) 35(12):73-5.

133. Fan W. Research on the utilization of health services of elderly residents with different basic medical insurance [Master].: Fudan University (2014).

134. Jia Y, Qi Y, Han Y, Xie Z, Ge J. Analysis of basic household hygiene in some areas of Heilongjiang Province. China Medical Herald. (2017) 14(18):56-8.

135. Li C. Research on the fairness of medical assistance for major diseases of rural residents [Master].: Huazhong University of Science and Technology (2016).

136. Lv D. Analysis of farmers' fundraising willingness and ability in pilot counties of new rural cooperative medical care in Shanxi Province [Master].: Shanxi Medical University (2005).

137. Sun J, Xie J, Yao Q, Yao L. Research on the Definition Method of Poverty Caused by Illness in China's Medical Assistance for Major and Extraordinarily Serious Diseases——Based on Empirical Evidence in M City, Hubei Province. China Health Policy Research. (2017) 10(04):1-7.

138. Sun X, Rehnbegr C, Meng Q. A study on catastrophic health expenditure of urban residents in Xining and Yinchuan. China Health Service Management. (2008) 25(1):12-5. doi: 10.3969/j.issn.1004-4663.2008.01.005.

139. Xu W, Chu F. Study on the level and influencing factors of catastrophic health expenditure--analysis based on CHARLS data. Social Security Research (5): 64-72 (2018).

140. Xu G, Yuan Z, Zhu H. A study on the changes of medical and health service demand and utilization of poor farmers under the influence of the new agricultural cooperation system. Modern Prev M. (2010) 37(21):4063-5.

141. Fang H. Research on Measuring Health Financing Equity: Analysis of Household Catastrophic Health Expenditure [Master].: Anhui Medical University (2004).

142. Hao X. Research on the Current Situation and Sustainable Development of New Rural Cooperative Medical Care in Jiangxi Province [Master].: Nanchang University; School of Medicine, Nanchang University (2010).

143. Shi D. Research on the economic risk of disease in low-income urban families: A case study of Xiangtan City, Hunan Province [Master].: Guangxi Medical University (2016).

144. Zuo Y, Wang X, Dai T. A study of catastrophic medical expenditure of hospitalized patients with six diseases in three cities. Health Economics Research. (2008) (11):28-9.

145. Zheng W, Zhao Z, Xu L. A comparative analysis of the economic burden of hospitalization of rural residents with other diseases. China Health Economics. (2006) (06):22-4.

146. Zhao Y, Tao S, Wan Q, Zhang Y, Huang J, Wang L. A case study of catastrophic health expenditure in rural households. China Health Economics. (2004) 23(4):5-8. doi: 10.3969/j.issn.1003-0743.2004.04.002.

147. Yang X. Research on Health Financing Equity Based on Financing System Function——A Case Study of Health Financing in S Province [Ph.D.].: Shandong University (2013).

148. Yang Y. Research on health financing equity of rural residents in Dali Bai Autonomous Prefecture, Yunnan Province [Master].: Kunming Medical University (2014).

149. Hua H. Evaluation of poverty alleviation effect of medical assistance project for advanced schistosomiasis patients in Jiangsu Province. Chinese Journal of Schistosomiasis Control. (2018):1-5.

150. Zang C. Measurement and influencing factors of catastrophic health expenditure of urban and rural residents in Gansu Province [Master].: North China University of Science and Technology (2016).

151. Chen J, Dong H, Yu H, Gu Y, Zhang T. Impact of new rural cooperative medical scheme on the equity of health services in rural China. Bmc Health Serv Res. (2018) 18(1). doi: 10.1186/s12913-018-3288-2.

152. Xu Y, Ma J, Wu N, Fan X, Zhang T, Zhou Z, et al. Catastrophic health expenditure in households with chronic disease patients: A pre-post comparison of the New Health Care Reform in Shaanxi Province, China. Plos One. (2018) 13(3): e194539. doi: 10.1371/journal.pone.0194539.

153. Zhang L, Cheng X, Tolhurst R, Tang S, Liu X. How effectively can the New Cooperative Medical Scheme reduce catastrophic health expenditure for the poor and non-poor in rural China? Trop Med Int Health. (2010). doi: 10.1111/j.1365-3156.2010.02469.x.

154. Zhen X, Zhang H, Hu X, Gu S, Li Y, Gu Y, et al. A comparative study of catastrophic health expenditure in Zhejiang and Qinghai province, China. Bmc Health Serv Res. (2018) 18(1). doi: 10.1186/s12913-018-3658-9.

155. Liu X, Sun X, Zhao Y, Meng Q. Financial protection of rural health insurance for patients with hypertension and diabetes: repeated cross-sectional surveys in rural China. Bmc Health Serv Res. (2016) 16(1). doi: 10.1186/s12913-016-1735-5.

156. Wang J, Chen L, Ye T, Zhang Z, Ma J. Financial protection effects of modification of China's New Cooperative Medical Scheme on rural households with chronic diseases. Bmc Health Serv Res. (2014) 14:305. doi: 10.1186/1472-6963-14-305. PubMed PMID: 25023600.

157. Sun J, Liabsuetrakul T, Fan Y, McNeil E. Protecting patients with cardiovascular diseases from catastrophic health expenditure and impoverishment by health finance reform. Trop Med Int Health. (2015) 20(12):1846-54. doi: 10.1111/tmi.12611.

158. Yang H, Huang M. Urban-rural disparities and allocation sensitivity of catastrophic health expenditure. China Health Policy Research. (2018) 11(07):24-9.

159. Chu J. Research on the impact of the adjustment of the new agricultural cooperation program on the utilization and benefit distribution of health services among rural residents with different incomes [Master].: Shandong University (2010).

160. Gu H, Kou Y, Yan Z, Ding Y, Shieh J, Sun J, et al. Income related inequality and influencing factors: a study for the incidence of catastrophic health expenditure in rural China. Bmc Public Health. (2017) 17(1). doi: 10.1186/s12889-017-4713-x.

161. Jing S, Yin A, Shi L, Liu J. Whether New Cooperative Mmedical Schemes reduce the economic burden of chronic disease in rural China. Plos One. (2013) 8(1): e53062. doi: 10.1371/journal.pone.0053062. PubMed PMID: 23326382.

162. C Li. Association of socioeconomic status with financial burden of disease among elderly patients with cardiovascular disease: evidence from the China Health and Retirement Longitudinal Survey. Bmj Open. (2017):1-10.


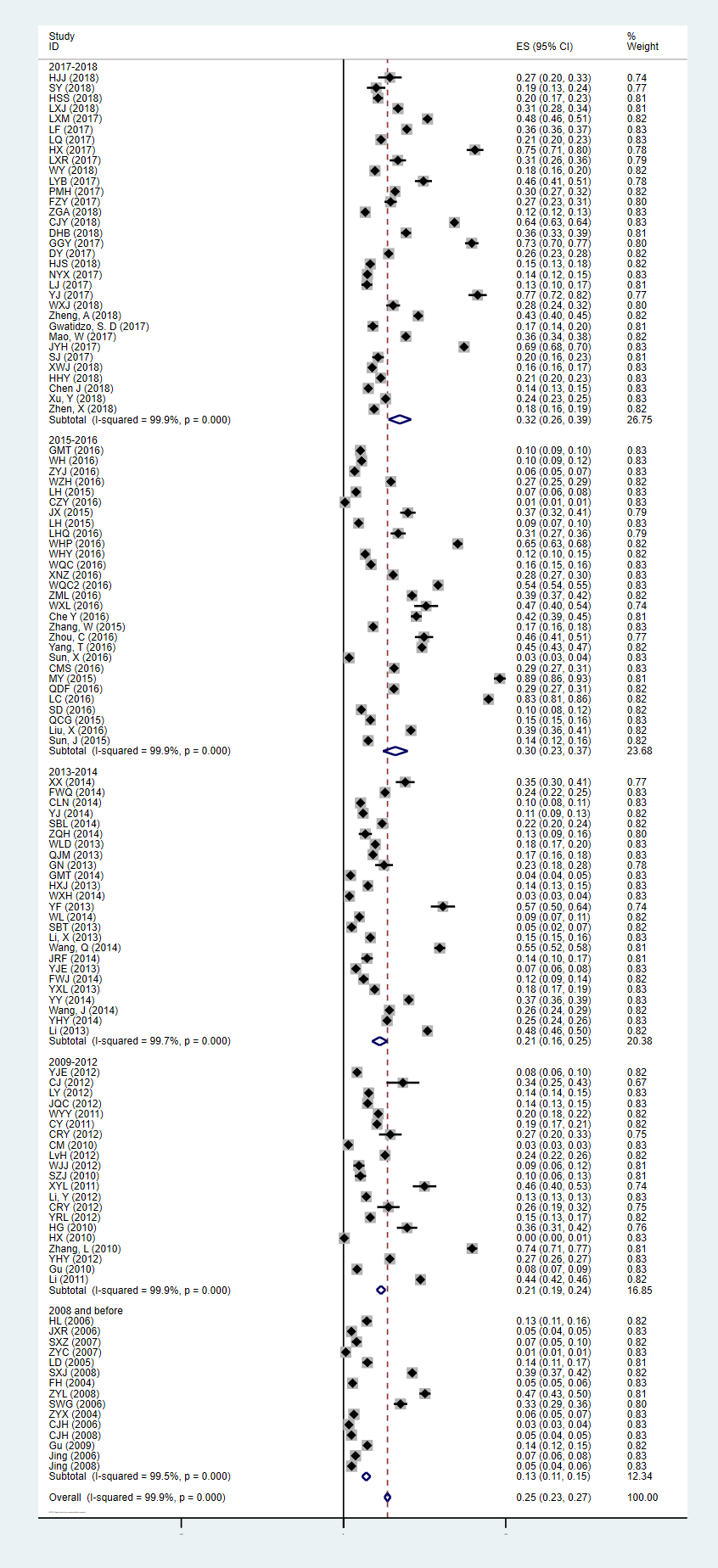


Appendix Figure 1. Secular trend of catastrophic health expenditure of all included studies


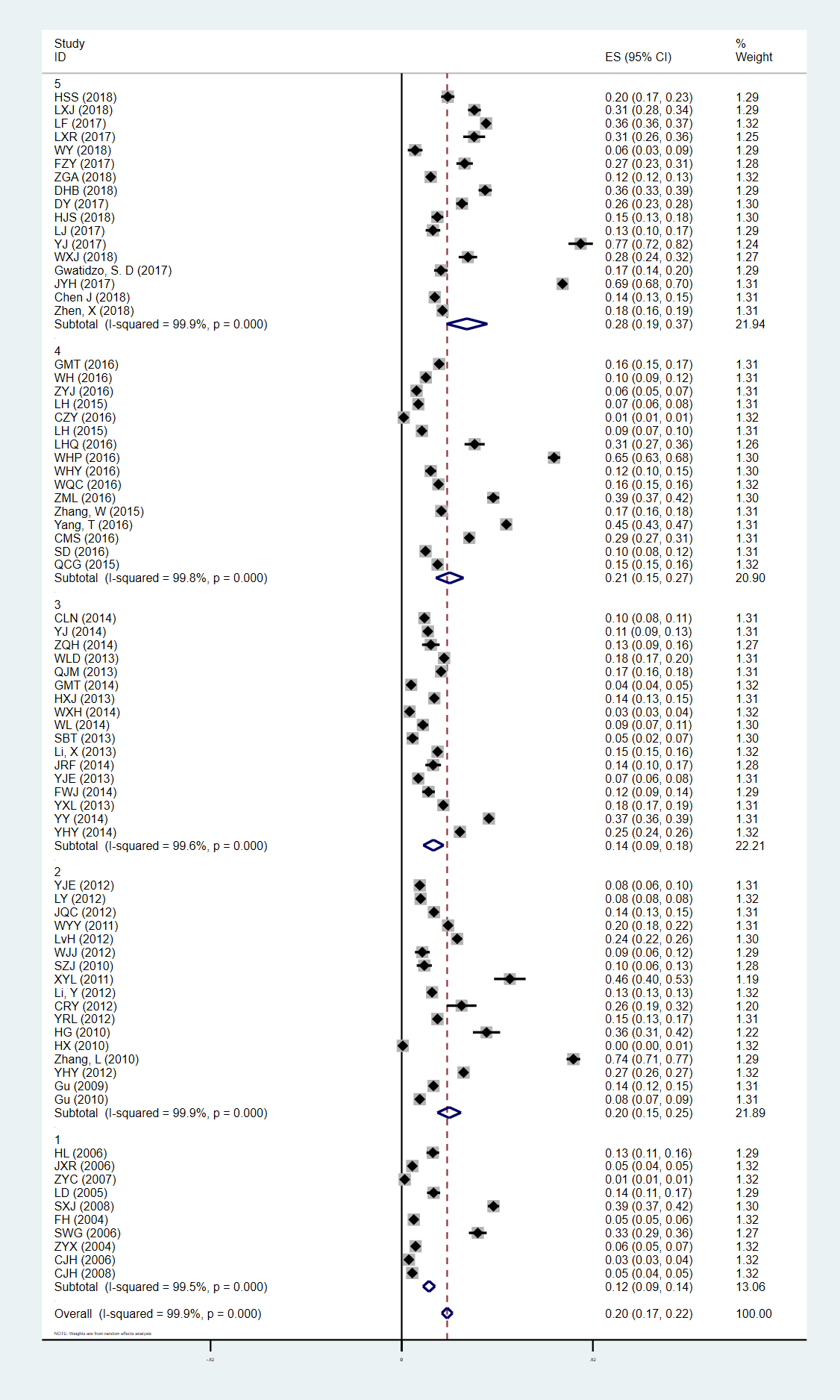


Appendix Figure 2. Secular trend of catastrophic health expenditure by the general population


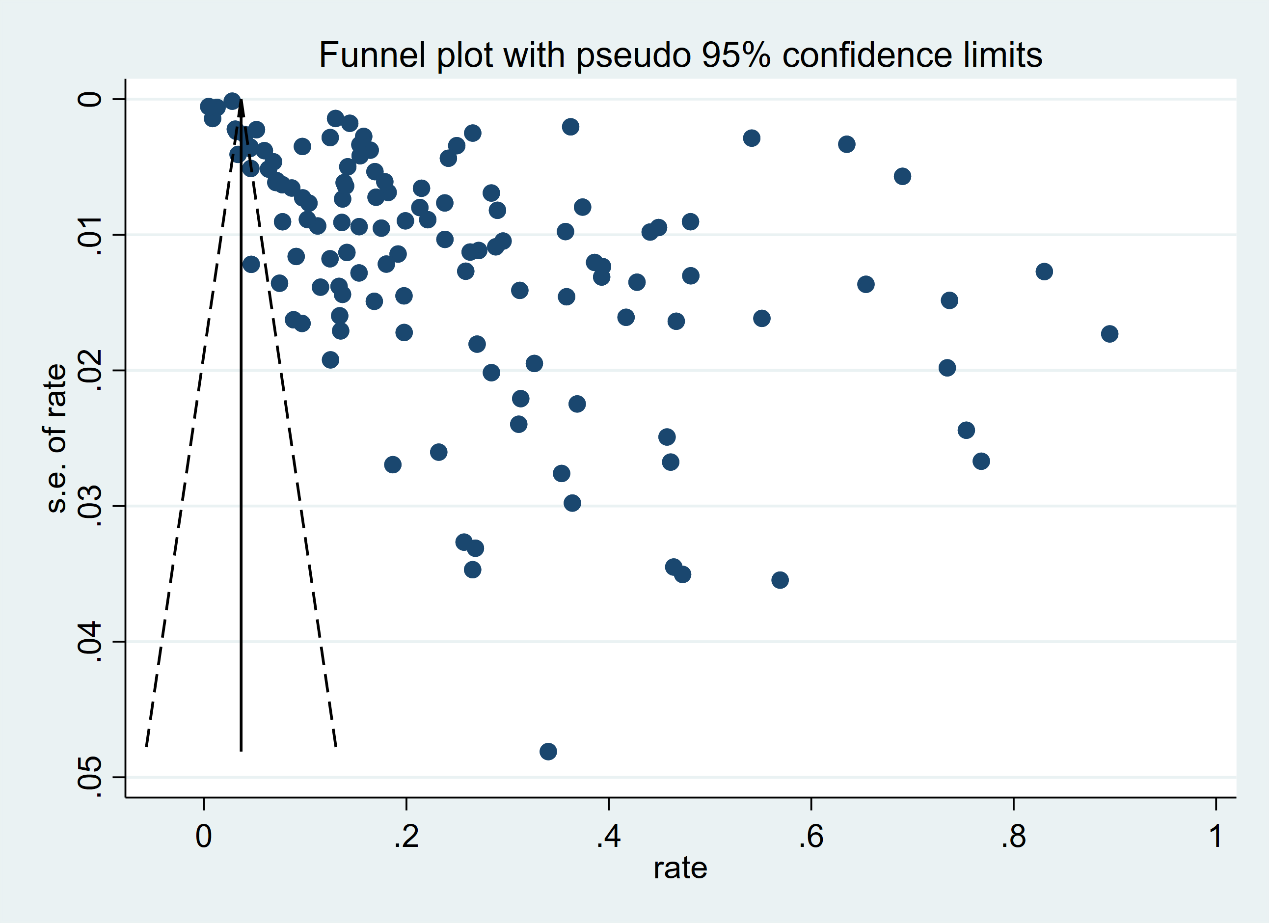


Appendix Figure 3. Publication bias funnel plot


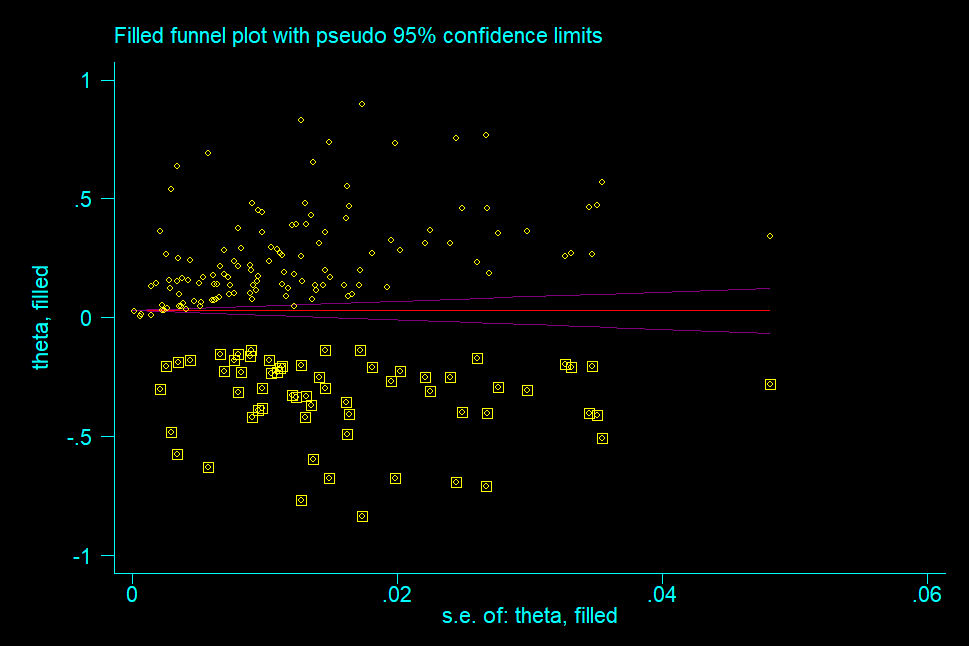


Appendix Figure 4. Trim and fill analysis funnel chart


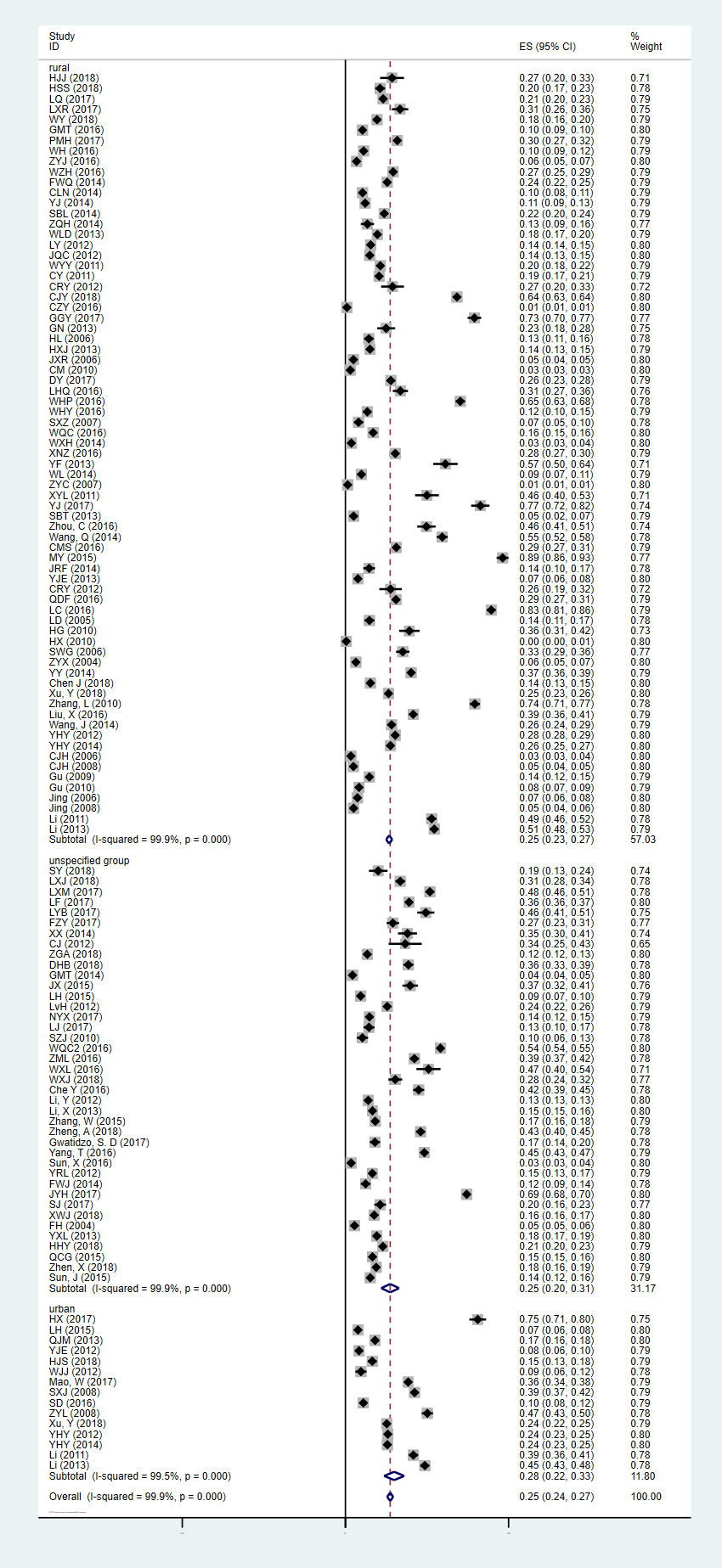


Appendix Figure 5. Rate of catastrophic health expenditure by Urban-rural differences


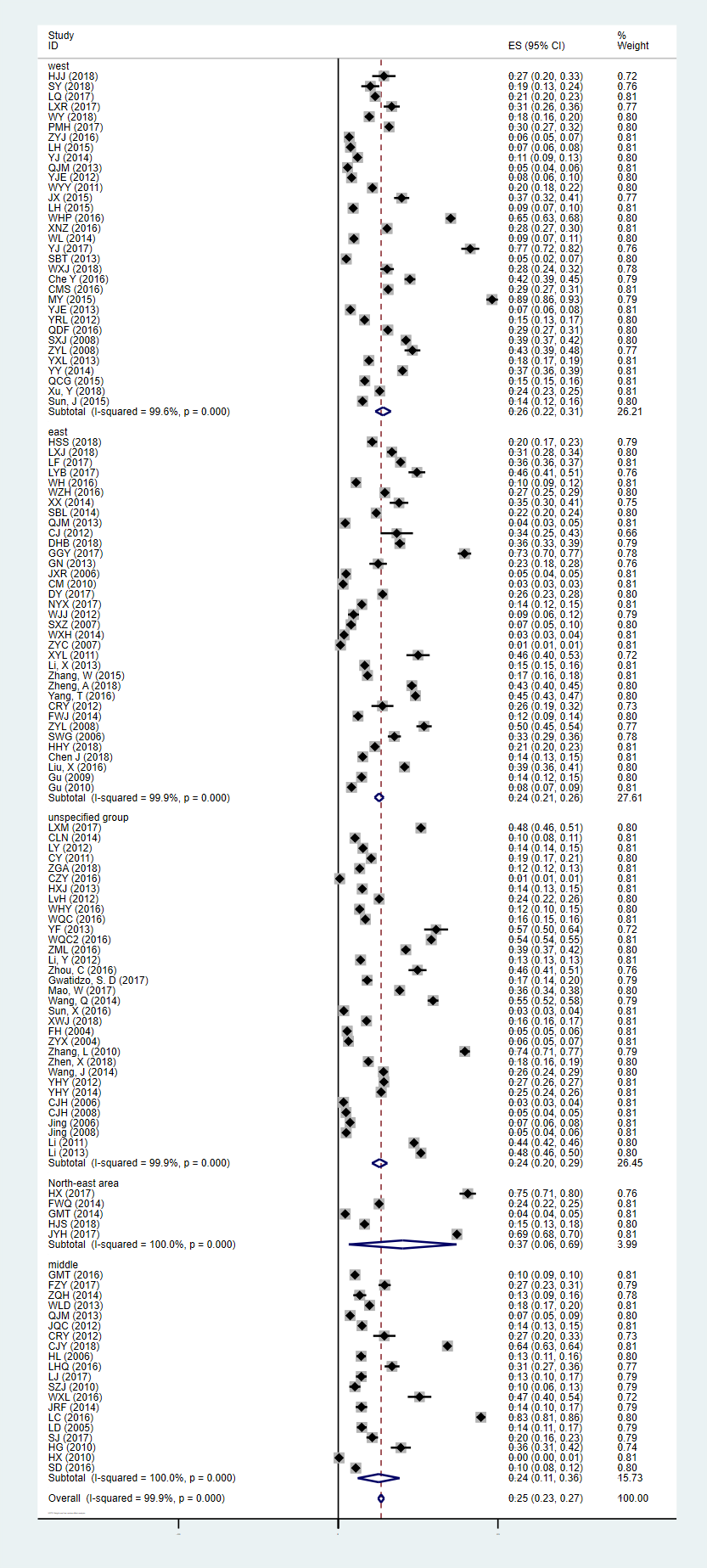


Appendix Figure 6. Rate of catastrophic health expenditure by the level of socio-economic status

Abbreviations: The eastern region included: Beijing, Tianjin, Hebei Provinces, Shandong Provinces, Jiangsu Provinces, Shanghai Provinces, Zhejiang Provinces, Fujian Provinces, Guangdong Provinces, and Hainan Provinces;

The Central region includes: Shanxi Provinces, Henan Provinces, Hubei Provinces, Hunan Provinces, Jiangxi Province, Anhui Province;

The western regions included: Chongqing City, Sichuan Province, Guangxi Zhuang Autonomous Region, Guizhou Province, Yunnan Province, Shaanxi Province, Gansu Province, Inner Mongolia Autonomous Region, Ningxia Hui Autonomous Region, Xinjiang Uygur Autonomous Region, Qinghai Province, Tibet Autonomous Region;

The northeast region included: Heilongjiang Province, Jilin Province, and Liaoning Province.


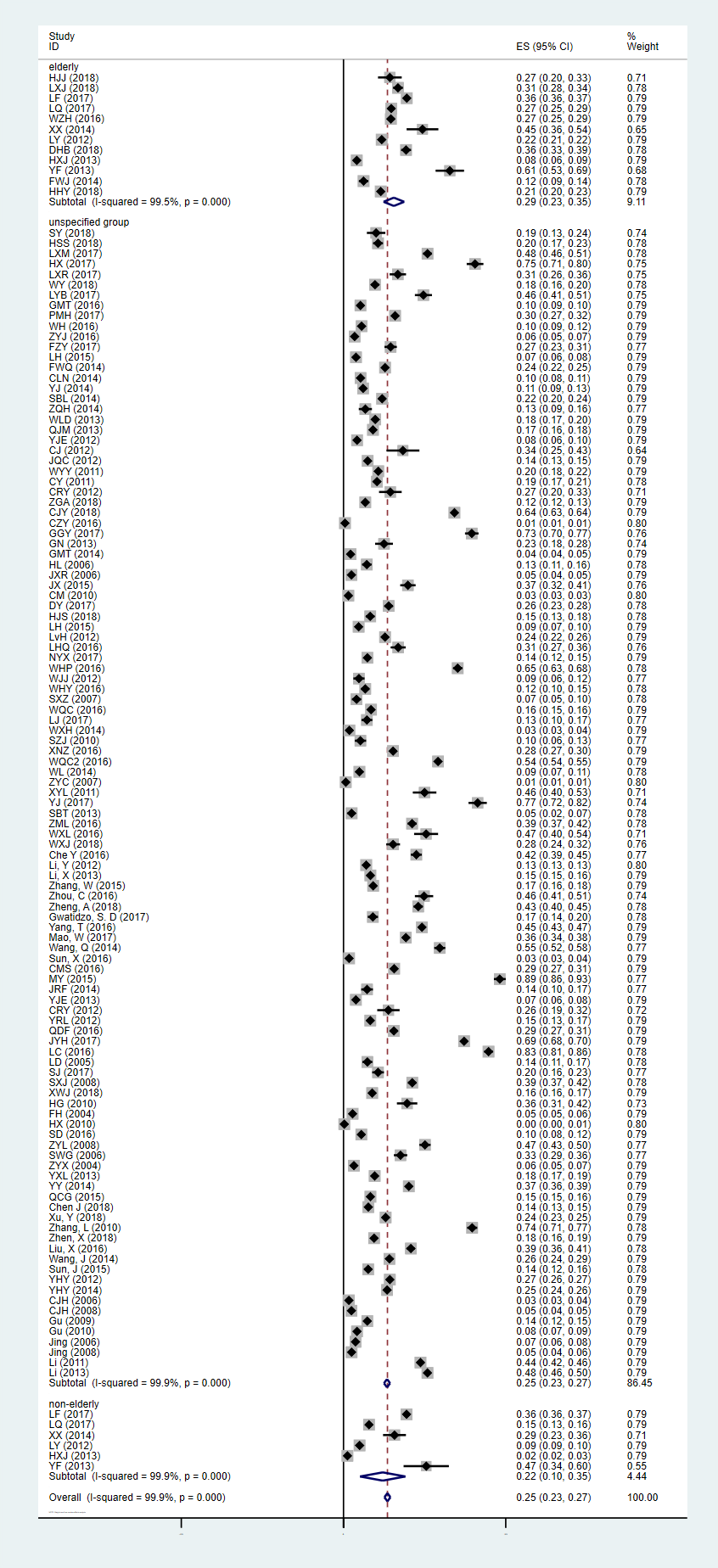


Appendix Figure 7. Rate of catastrophic health expenditure by age


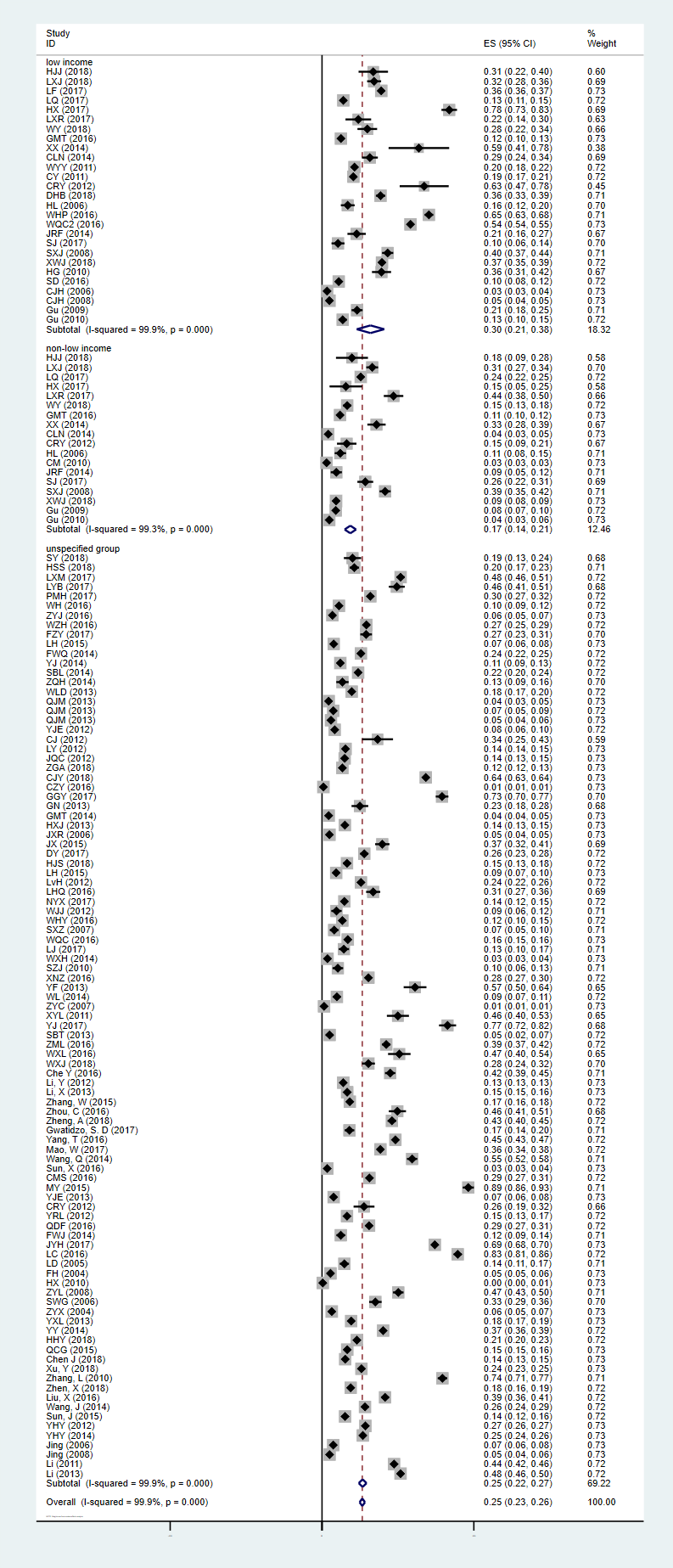


Appendix Figure 8. Rate of catastrophic health expenditure by income level


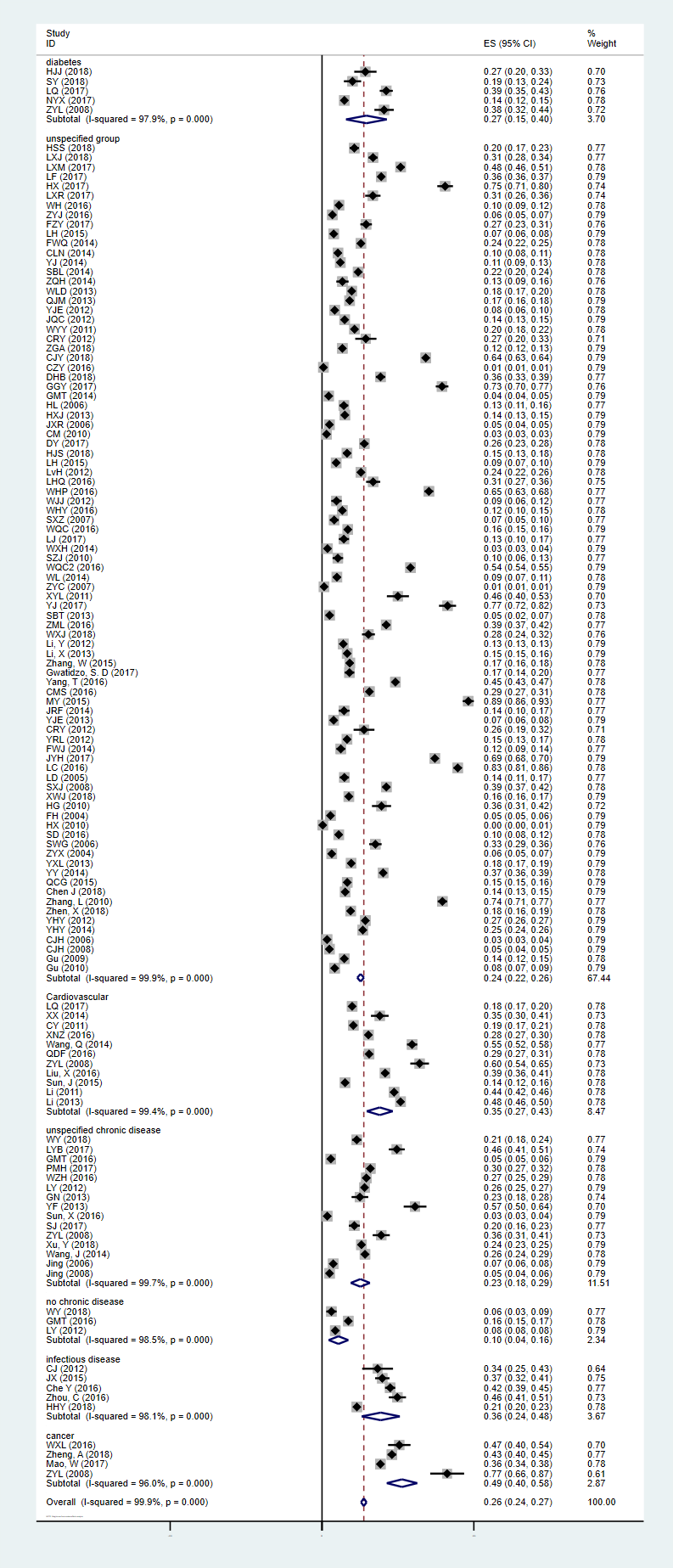


Appendix Figure 9. Rate of catastrophic health expenditure by diseases


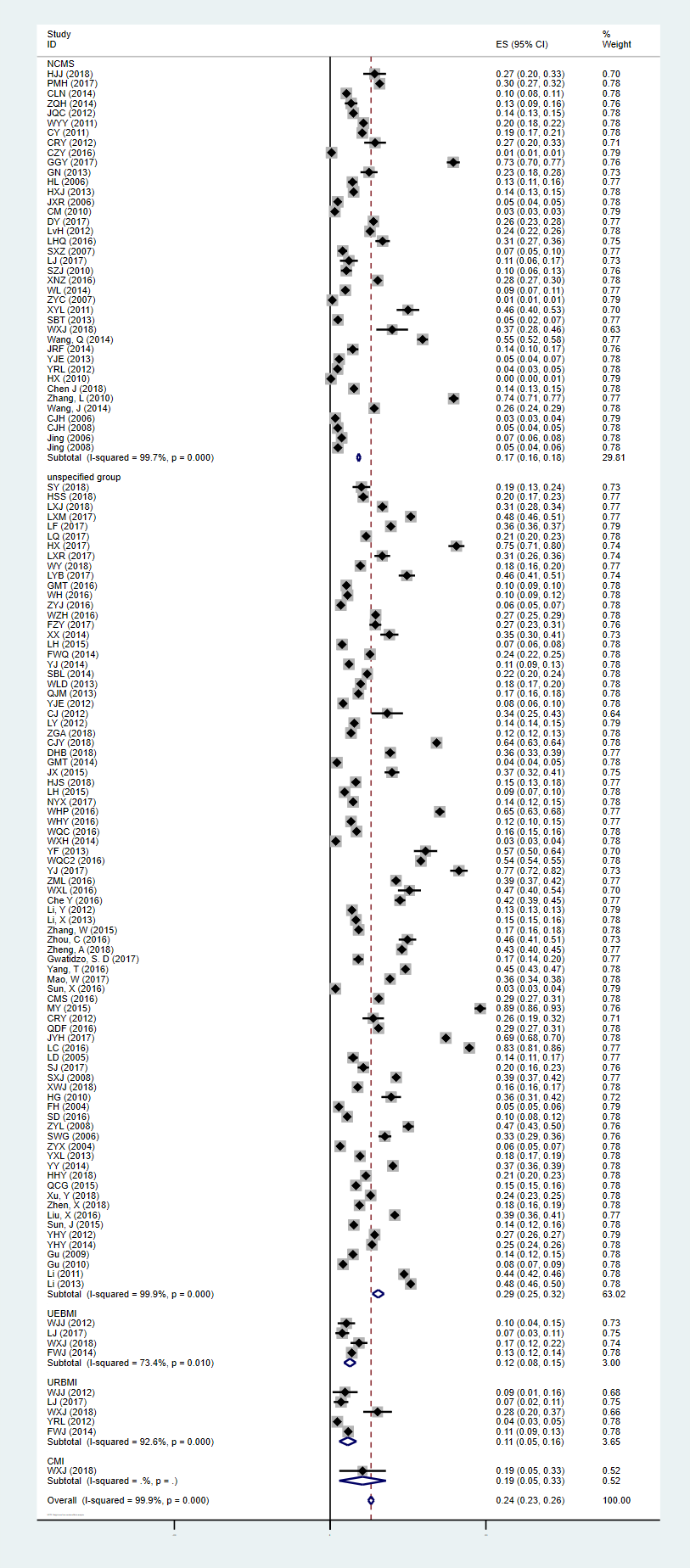


Appendix Figure 10. Rate of catastrophic health expenditure by medical insurance


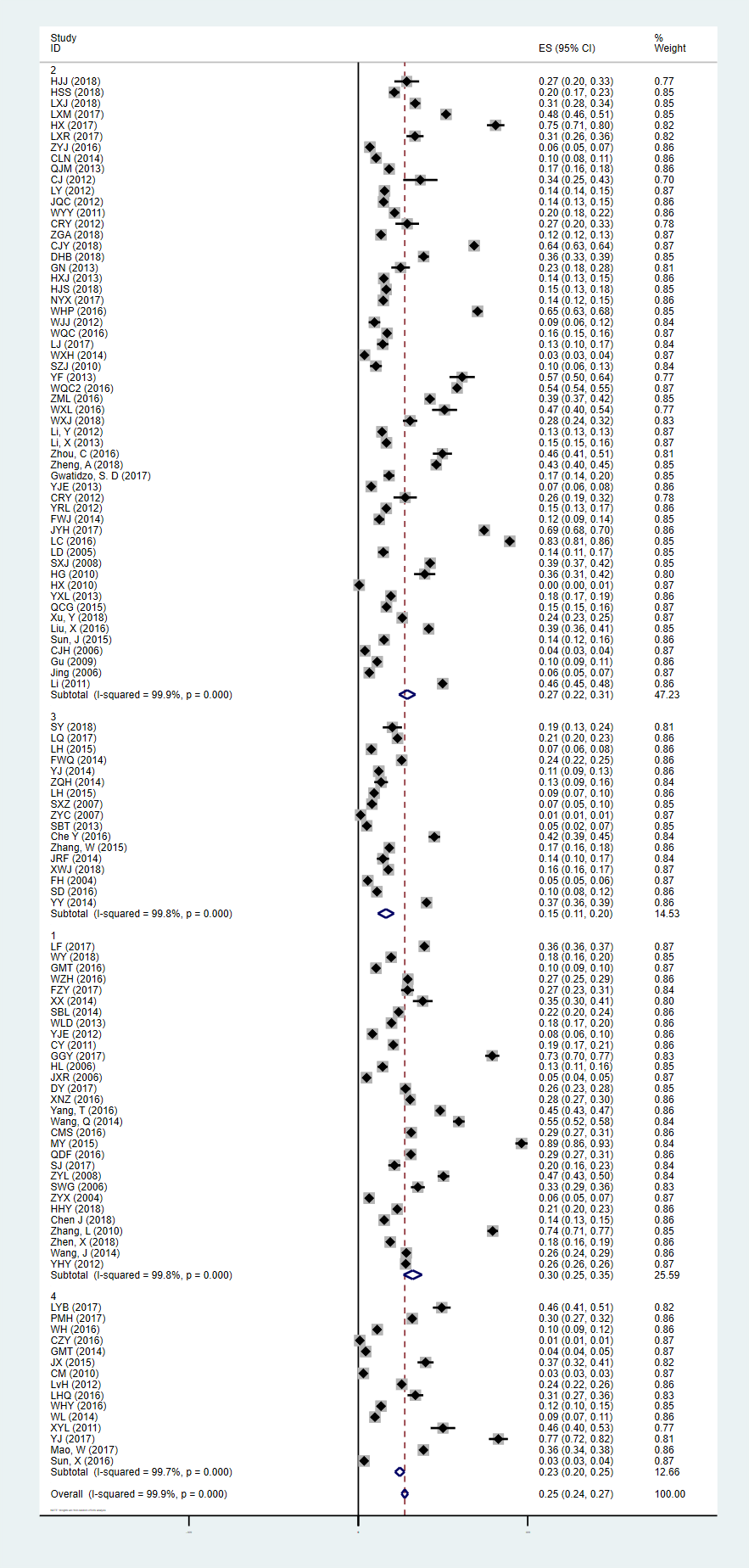


Appendix Figure 11. Rate of catastrophic health expenditure by its definition
