# Supplementary material for: Catastrophic health expenditure, incidence, trend and socioeconomic risk factors in China: A systematic review and meta-analysis
Source: Front Public Health. 2023 Jan 4;10:997694. doi: 10.3389/fpubh.2022.997694 (PMC9846062; doi:10.3389/fpubh.2022.997694)
Supplement: Supplementary file 2 [file Table_2.DOCX]

AppendixTable 1. Search strategy

| Keywords |
| --- |
| 1、Catastrophic health expenditure  2、Catastrophic medical expenses  3、Poverty-causing health expenditure  4、Poverty due to illness  5、Return to poverty due to illness  6、1 or 2 or 3 or 4 or 5  7、China  8、6 and 7 |

Appendix Table 2. Meta regression analysis of the influencing factors of catastrophic health expenditure

| Variable name | Coefficient | t | P | [95%Conf.Interval] | |
| --- | --- | --- | --- | --- | --- |
| rural | 0.00 | -0.07 | 0.947 | -0.08 | 0.07 |
| urban | 0.02 | 0.4 | 0.688 | -0.09 | 0.14 |
| Eastern region | 0.01 | 0.2 | 0.84 | -0.10 | 0.12 |
| North-east region | 0.16 | 1.83 | 0.07 | -0.01 | 0.33 |
| Western region | 0.03 | 0.56 | 0.574 | -0.08 | 0.14 |
| Unspecified group | 0.01 | 0.11 | 0.911 | -0.10 | 0.11 |
| Elderly | -0.04 | -0.42 | 0.674 | -0.24 | 0.15 |
| Unspecified group | -0.05 | -0.57 | 0.568 | -0.21 | 0.12 |
| Low-income | 0.12 | 2.04 | 0.043 | 0.00 | 0.23 |
| Unspecified group | 0.07 | 1.48 | 0.141 | -0.02 | 0.17 |
| Cancer | 0.40 | 2.73 | 0.007 | 0.11 | 0.69 |
| Cardio-cerebro-vascular diseases | 0.25 | 2.03 | 0.044 | 0.01 | 0.50 |
| Diabetes | 0.17 | 1.23 | 0.223 | -0.11 | 0.45 |
| Major infectious disease | 0.26 | 1.85 | 0.067 | -0.02 | 0.54 |
| Unspecific chronic disease | 0.14 | 1.13 | 0.262 | -0.10 | 0.38 |
| Unspecified group | 0.14 | 1.22 | 0.224 | -0.09 | 0.36 |
| NCMS | 0.07 | 0.81 | 0.42 | -0.10 | 0.25 |
| UEBMI | 0.00 | 0.01 | 0.992 | -0.25 | 0.25 |
| CMI | 0.08 | 0.36 | 0.721 | -0.35 | 0.50 |
| Unspecified group | 0.17 | 1.97 | 0.050 | 0.00 | 0.34 |
| Definition 1 | 0.15 | 2.53 | 0.013 | 0.03 | 0.26 |
| Definition 2 | 0.12 | 2.2 | 0.030 | 0.01 | 0.22 |
| Definition 4 | 0.09 | 1.36 | 0.175 | -0.04 | 0.23 |

Appendix Table 3. Study quality scores

| Quality scores | Research type | | | | | |
| --- | --- | --- | --- | --- | --- | --- |
|  | Cross-sectional | | Case-control | | Cohort | |
|  | Numbers | Weight (%) | Numbers | Weight (%) | Numbers | Weight (%) |
| 1 | 0 | 0.0 | 0 | 0.0 | 0 | 0.0 |
| 2 | 1 | 1.2 | 0 | 0.0 | 0 | 0.0 |
| 3 | 12 | 14.8 | 0 | 0.0 | 0 | 0.0 |
| 4 | 17 | 21.0 | 5 | 15.6 | 0 | 0.0 |
| 5 | 32 | 39.5 | 7 | 21.9 | 4 | 80.0 |
| 6 | 19 | 23.5 | 14 | 43.8 | 1 | 20.0 |
| 7 | 0 | 0.0 | 5 | 15.6 | 0 | 0.0 |
| 8 | 0 | 0.0 | 1 | 3.1 | 0 | 0.0 |
| total | 81 | 100.0 | 32 | 100.0 | 5 | 100.0 |

Appendix Table 4. GRADE summary of findings

| **Certainty assessment** | | | | | | | **Certainty** | **Effect**  **(95%CI)** |
| --- | --- | --- | --- | --- | --- | --- | --- | --- |
| **№ of studies** | **Study design** | **Risk of bias** | **Inconsistency** | **Indirectness** | **Imprecision** | **Other considerations** |  |  |
| 118 | Observational  studies | serious^a^ | serious^b^ | Not serious | Not serious | High publication bias^c^ | ⨁◯◯◯ Very low | 25.2%  (23.4%-26.9%) |

**CI:** Confidence interval

**Explanations**

a. Downgraded by one level for serious risk of bias

b. Downgraded for high inconsistency

c. Downgraded for high publication bias

Appendix Table 5. Characteristics of included studies

| Author/ year | region | study design | participants | N | definition of CHE | quality score |
| --- | --- | --- | --- | --- | --- | --- |
| HJJ2018(41) | Midu County, Yunnan Province, China | Cross-sectional | Eight townships/towns within the jurisdiction of Midu County, Yunnan Province, randomly sampled diabetic patients in multiple stages according to income level | 162 | Households have paid more than 40% of their disposable income (total annual household income - food and education expenditure) in the past year | 6 |
| SY2018(42) | Shaanxi Province, China | Cross-sectional | People with diabetes in the fifth National Health Service Survey conducted in Shaanxi Province from September to October 2013 | 209 | Household medical and health expenditure accounts for 40 percent of household ≥ income-expenditure | 5 |
| HSS2018(43) | Shandong Province, China | Cross-sectional | In Shandong Province, random sampling was stratified according to the economic level | 754 | Out-of-pocket medical expenses account for 40% of household non-food expenses | 6 |
| LXJ2018(44) | Jiangsu Province, China | Cross-sectional | China Health and Pension Tracking CHALS survey 2013 Jiangsu Province follow-up survey of people over 45 years of age | 1080 | Families pay for medical expenses in household non-food consumption spending exceeds 40% | 4 |
| LXM2017(45) | China | Cross-sectional | The Ministry of Education's major research project on philosophy and social sciences, "Perfecting the Research on social assistance system,” is a low-security survey | 1472 | Household out-of-pocket pays more than 40% of household consumption for health care | 6 |
| LF2017(46) | Shanghai, China | Cross-sectional | In 2015, shanghai civil medical assistance for low-insured, low-income families, severely disabled and unemployed, and particular relief objects for civil affairs | 55500 | Out-of-pocket medical expeness≥ 40% of total personal consumption expenditure | 3 |
| LQ2017(47) | Rural areas of Yunnan Province, China | Cross-sectional | From January to September 2015, a random sample of multi-stage stratified random samples according to economic level was selected to conduct a questionnaire survey in Yunnan Province | 3909 | For a certain period, households spend more on medical care than a certain proportion of disposable household capacity, which has a disastrous impact on average household consumption, at 40% standards. | 6 |
| HX2017(48) | Jilin Province, China | Cross-sectional | Low-income families identified by the local Civil Administration as having low-security families with chronic diseases who had been treated for chronic diseases in the year before the survey | 312 | Household out-of-pocket payments for medical expenses account for more than 40% of household non-food consumption expenditure | 4 |
| LXR2017(49) | Yinchuan City, Gansu Province, China | Cross-sectional | A random group sampling method to extract the residents of Yinchuan city's rural areas | 373 | Household health expenditure accounts for 40% of household non-food consumption | 6 |
| WY2018(50) | Fushun County, Sichuan Province, China | Cross-sectional | In Fushun County, Sichuan Province, a random sample of rural residents participating in the new agricultural joint was selected at multiple stages | 999 | Household out-of-pocket payments for health care account for more than 40% of household consumer spending | 6 |
| LYB2017(51) | Jiangsu Province, China | Cross-sectional | Randomly collected 400 patients who had been admitted to a Chinese medicine hospital in a city in Jiangsu Province in 2014 because of a severe illness | 400 | In this study, the occurrence of catastrophic expenditure on inpatients in rural residents was measured by the medical expenses of rural residents exceeding 3083 yuan | 2 |
| GMT2016(52) | Rural areas of Hubei Province, China | Cross-sectional | "Fifth Health Service Survey in Hubei Province" in 2013 | 7202 | Residents' out-of-pocket and health expenditures account for more than 40% of households' ability to pay | 5 |
| PMH2017(53) | Zhongjiang County, Sichuan Province, China | Cross-sectional | In the new agricultural joint information system, the information of the third-level hospital in Deyang City in 2010 was derived from the farmers participating in the county | 1902 | Out-of-pocket out-of-the-way expenses exceed the annual per capita net income of farmers | 6 |
| WH2016(54) | Qianxi County, Hebei Province, China | Cross-sectional | The health inquiry data of the new rural cooperative medical family were collected in the county of Qianxi, Hebei Province | 1581 | The ROC curve of the result of poverty due to disease is drawn by the burden of household health expenditure, which corresponds to the critical value of the burden of household health expenditure when the index is at its highest, which is 38% in this paper | 5 |
| ZYJ2016(55) | Sichuan Province, China | Cross-sectional | Rural families in Fushun County, Zigong, Sichuan Province, 2012 | 2244 | Household out-of-pocket hygiene expenditure accounts for 40% of household non-food consumption expenditure | 6 |
| WZH2016(56) | Rural areas of Jiangsu Province, China | Cross-sectional | Data from the 2013 survey on the economic burden of disease in rural areas of Jiangsu Province were relevant to the financial burden of illness in elderly chronically ill families | 1588 | Out-of-payments health care expenditure as a percentage of household consumption expenditure exceeds 40% | 5 |
| FZY2017(57) | A city in western Hubei Province, China | Cross-sectional | Household survey data obtained in 2015 from a stratified random sample in the city | 604 | Household medical expenses (oop, out-of-pocket expenditure portion) as a percentage of household affordability more than 40% | 4 |
| XX2014(58) | Guangdong Province, China | Cross-sectional | In 2012, 300 patients with stroke were diagnosed at the Hospital of the Stroke Screening and Prevention Base in northern Guangdong Province, where the hukou is located in Shaoguan City | 300 | Household out-of-pocket payments for health care (OOP) account for more than 40% of household expenditure | 5 |
| LH2015(59) | Hami City, Xinjiang, China | Cross-sectional | 2013 Hami District Residents' Family Health Inquiry Survey | 1845 | The ratio of household health expenditure to household consumption expenditure exceeds 40% | 5 |
| FWQ2014(60) | Rural areas of Heilongjiang Province, China | Cross-sectional | Rural families surveyed by the Fourth Health Service survey in Heilongjiang Province in 2008 | 3098 | Health expenditure accounts for more than 40% of household consumption expenditure | 3 |
| CLN2014(61) | Zhejiang, Hubei and Chongqing, China | Cross-sectional | An on-site investigation in Chongqing, Hubei Province, Zhejiang Province, China | 1661 | Household out-of-pocket health care costs account for more than 40% of household non-food consumer spending | 5 |
| YJ2014(62) | Rural Lushan County, Dalizhou, Yunnan Province, China | Cross-sectional | Rural families in Lushan County, Dalizhou | 1140 | 40 percent of household health financing contribution (household health expenditure/household affordable household > 40 percent | 4 |
| SBL2014(63) | Jinan, Shandong Province, China | Cross-sectional | Survey data on the demand and utilization of health services for rural residents in Jinan in 2012 | 2180 | Family members' out-of-pocket and health expenditures account for more than 40% of household consumption | 4 |
| ZQH2014(64) | Heilongjiang Province, China | Cross-sectional | Families who suffered catastrophic health expenditures and received insurance compensation in rural Heilongjiang Province in 2007 | 296 | 40 percent is used as a criterion for the classification of catastrophic health expenditure, based on the standard of family expenditure representing family life | 3 |
| WLD2013(65) | New Agricultural Co-pilot County, Anhui Province, China | Cross-sectional | A random sample of six pilot counties in Xinnong, Anhui Province, was randomly selected according to the economic level | 3149 | Personal out-of-pocket health expenditure accounts for more than 40% of household consumption | 4 |
| QJM2013(66) | Eight cities in China | Cross-sectional | A random sample of six pilot counties in Xinnong, Anhui Province, was randomly selected according to the economic level | 4915 | Household out-of-pocket payments account for 40% of non-food expenses | 5 |
| YJE2012(67) | Mei County, Baoji City, Shaanxi Province | Cross-sectional | A sample household survey of urban residents in Mei County, Shaanxi Province | 876 | Households pay more than 40 percent of household disposable income for health care in out-of-pocket | 3 |
| CJ2012(68) | Shanghai, China | Cross-sectional | In 2008, the first treatment of active tuberculosis patients from the non-resident population was newly registered and completed in Putuo District | 97 | Household health expenditure exceeds 40% of household non-food expenditure | 4 |
| LY2012(69) | Rural China | Cross-sectional | The Fourth National Health Service Survey of Rural Families | 38945 | Household out-of-pocket hygiene expenditure exceeds 40% of household non-food expenditure | 4 |
| JQC2012(70) | Rural areas of Anhui Province, China | Cross-sectional | In 2009, Anhui Province stratified random group sampling survey | 3149 | Family members' out-of-pocket hygiene expenditure accounts for more than 40% of household non-food consumption expenditure | 5 |
| WYY2011(71) | Rural areas of Sichuan Province, China | Cross-sectional | Family health was investigated in the Fourth Health Service Survey of Sichuan Province in 2008  Ask about the survey of rural low-income families in the western expansion date | 1980 | Household out-of-pocket hygiene expenditure accounts for 40% of household non-food expenditure | 5 |
| CY2011(72) | Ledu County, Qinghai Province, Gansu Province, and Zheng County, Shanxi Province, Tocheng County, and Ping lu County | Cross-sectional | The on-site investigation of the demonstration village of hypertension system management has confirmed the patients with primary hypertension and stroke, and coronary heart disease who township hospitals have diagnosed and above medical institutions | 1189 | Household health expenditure in out-of-pocket accounts for more than 40% of household income | 5 |
| CRY2012(73) | Tengzhou City, Shandong Province, China | Cross-sectional | A sample survey was conducted in 2 towns in Tengzhou City, Shandong Province | 179 | Household out-of-pocket hygiene expenditure accounts for more than 40% of household non-food expenditure | 5 |
| ZGA2018(74) | China | Cross-sectional | 2014 CFPS survey data | 13602 | Household out-of-pocket accounts for 40% or more of household non-food expenses | 5 |
| CJY2018(75) | Ezhou City, Hubei Province, China | Cross-sectional | Ezhou City 2016 "due to disease poverty, due to illness back to poverty" population data | 20961 | Household out-of-pocket accounts for 40% or more of household non-food expenses | 4 |
| CZY2016(76) | China | Cross-sectional | CHNS database collected jointly by the University of North Carolina's Carolina Population Center and the China Centers for Disease Control and Prevention | 4120 | Higher-income households: Health expenditure accounts for more than 25% of household per capita income | 4 |
| DHB2018(77) | Hebei Province, China | Cross-sectional | Field investigation field interview in Hebei Province | 1083 | Household out-of-pocket accounts for 40% or more of household non-food expenses | 3 |
| GGY2017(78) | China | Cross-sectional | A sample survey of the whole group of patients with critical illnesses in Beijing | 497 | Out-of-pocket health expenditure accounts for 40% of household consumption | 3 |
| GN2013(79) | Zhangqiu City, Changqing District, Pinyin County, Shandong Province, China | Cross-sectional | A stratified random sampling survey | 263 | Household out-of-pocket hygiene expenditure accounts for more than 40% of household non-food expenditure | 4 |
| GMT2014(80) | Heilongjiang Province, China | Cross-sectional | Data from Heilongjiang Province in the Fourth National Health Service Survey | 5661 | When the total household expenditure is equal to or greater than the basic living expenses, and the entire household expenditure excluding out-of-pocket health expenditure is less than the basic living expenses | 5 |
| HL2006(81) | Shanxi Province, China | Cross-sectional | In 2004, 607 peasant families in poor counties in Shanxi Province conducted a quantitative survey on the financing and willingness to participate in new rural cooperative medical care | 607 | Household out-of-pocket payments account for more than 40% of household consumption | 5 |
| HXJ2013(82) | China | Cross-sectional | CHNS-related data for 2009 | 2931 | Household out-of-pocket accounts for more than 40% of household consumption expenditure | 4 |
| JXR2006(83) | Shanghai, China | Cross-sectional | A sample survey of the new rural cooperative medical operation mode in the suburbs of Shanghai | 3494 | Household out-of-pocket accounts for more than 40% of household consumption expenditure | 3 |
| JX2015(84) | Yunnan Province, China | Cross-sectional | A sample survey of AIDS patients in Dalizhou, Yunnan Province | 461 | The average net income of households is the sum of the total net income of households/the total number of respondents, and the maximum capacity to pay is the net income of 1/2 households | 6 |
| CM2010(85) | Ningbo, China | Cross-sectional | Ningbo Cixi, Fenghua rural residents medical institutions file records | 1215300 | Paying for medical care exceeds a specific value to bring the standard of living below the poverty line | 5 |
| DY2017(86) | Fuzhou, China | Cross-sectional | China Health Statistics Yearbook, Fuzhou City Statistical Yearbook, Fuzhou City Health and Family Planning Commission's new agricultural joint management department records, Fuzhou City participating in the rural residents hospitalized patients or their families questionnaire | 1191 | A percentage of residents' out-of-pocket and health expenditure exceeds total household consumption expenditure (40%) | 6 |
| HJS2018(87) | Fuzhou, China | Cross-sectional | Monitoring data from the State Forestry Administration's "Reform Monitoring of Key State-owned Forest Areas" project in 2016 | 790 | Direct household medical expenses account for more than 25% of household non-food expenses | 5 |
| LH2015(88) | Hami City, Xinjiang, China | cross sectional | Data from the 2013 Health Services Survey in Hami, Xinjiang | 1845 | The ratio of household health expenditure to household consumption expenditure exceeds 40% | 5 |
| LvH2012(89) | Zhejiang, Hubei and Chongqing, China | Cross-sectional | Sample survey in Zhejiang, Hubei, and Chongqing, China, 2011 | 1697 | Out-of-pocket health expenditure as a percentage of income exceeds 30% | 5 |
| LHQ2016(90) | Hubei Province, China | Cross-sectional | Hubei Province A land household survey, new agricultural joint agency records | 441 | Health spending for people with significant illnesses accounts for more than 40% of household affordability | 4 |
| NYX2017(91) | Shandong Province, China | Cross-sectional | Two urban communities, Fushan District of Yantai City, Weifang City, Weicheng District, and four rural counties, Luyuan County, Liangshan County, Dairy Mountain City, Gaotang County, conducted a questionnaire survey | 2183 | Families with diabetes pay more than 40% of household non-food expenses | 5 |
| WHP2016(92) | Nine provinces and cities in China | Cross-sectional | The poverty alleviation task force investigated poor villages in poor counties | 1214 | Household out-of-pocket medical expenses exceed non-food expenses by 40% | 3 |
| WJJ2012(93) | Shijiazhuang, Hebei Province, China | Cross-sectional | Shijiazhuang City, urban households into the household survey | 305 | Household out-of-pocket medical expenses exceed non-food expenses by 40% | 5 |
| WHY2016(94) | China | Cross-sectional | Data on rural residents in the CHNS survey for 2011 | 787 | Health expenditure accounts for more than 10% of household income | 5 |
| SXZ2007(94) | Shandong Province, China | Cross-sectional | A sample survey of whole stratified groups in the pilot county of Xinnong joint in Shandong Province | 375 | 40.00% of household health expenditure exceeds the capacity to pay | 3 |
| WQC2016(96) | China | Cross-sectional | The 2014 China Household Tracking Survey surveyed rural households | 17587 | Health care accounts for 40% of household non-food spending | 6 |
| LJ2017(97) | Shanxi Province, China | Cross-sectional | A sample survey of households in Taiyuan City | 455 | Out-of-pocket medical expenses account for more than 40% of household non-food expenses | 5 |
| LXH2014(98) | Jiangsu Province, China | Cross-sectional | A stratified random sampling questionnaire in Jiangsu Province | 1960 | Out-of-pocket medical expenses account for more than 40% of household non-food expenses | 5 |
| SZJ2010(99) | Huaihua City, China | Cross-sectional | A random sample survey of Huaihua City | 320 | Out-of-pocket medical expenses account for more than 40% of household non-food expenses | 4 |
| XNZ2016(100) | Chongqing, China | Cross-sectional | The fifth NHSS Chongqing rural areas of hypertension population survey | 4244 | Out-of-pocket medical expenses for family members exceed 40% of household disposable income | 5 |
| YF2013(101) | Hebei, Henan and Yunnan, China | Cross-sectional | Families with maternal deaths in rural areas of Yunnan, Henan Province, Hebei Province | 195 | The disease costs more than 40% of household non-food expenses | 3 |
| WQC22016(102) | China | Cross-sectional | Data from the 2014 China Household Tracking Survey | 30062 | Household out-of-pocket payments for medical expenses account for more than 40% of household non-food consumption expenditure | 4 |
| WL2014(103) | Zunyi City, China | Cross-sectional | A random sample site survey in Zunyi City, Guizhou, in 2010 | 615 | The standard of living of families for paying for medical care is below the poverty line | 3 |
| ZYC2007(104) | Huairou District, Beijing, China | Cross-sectional | New agricultural statistics for Huairou District | 33411 | Health expenditure accounts for 40% of household income | 3 |
| XYL2011(105) | Guangdong Province, China | Cross-sectional | Guangdong Province, the new rural cooperative medical sustainable development research and analysis team in 2008 household survey | 209 | Residents earn less than the local minimum living security line after paying for medical care | 3 |
| YJ2017(106) | Tongchuan, China | Cross-sectional | A questionnaire survey of farmers in Indo-Tai District, Tongchuan City | 250 | The standard of living of the population is lower than the local poverty line because of the cost of health | 5 |
| SBT2013(107) | Yulong County, Yunnan Province, China | Cross-sectional | Yulong County sample survey | 300 | Households have paid more than 40% of their total medical expenses in the past year | 4 |
| ZML2016(108) | China | Cross-sectional | CHARLS 2013 survey data | 1387 | Health care costs paid in out-of-pocket account for 40 percent of household non-food expenditure | 6 |
| WXL2016(109) | Nanchang, China | Cross-sectional | A questionnaire survey of lung cancer patients in Nanchang | 203 | Household health care costs account for more than 40% of household non-food expenses | 6 |
| WXJ2018(110) | Guangxi Zhuang Autonomous Region, China | Cross-sectional | A hospital affiliated with a university in Guangxi was hospitalized for myocardial infarction in 2016-2017 | 500 | Out-of-pocket medical expenses account for more than 40% of household non-food expenditure throughout the year | 5 |
| Che Y2016(111) | Yunnan Province, China | Cross-sectional | Patients with no complications of chronic HBV over 20 years of age in Yunnan Province, patients with reparational HBV cirrhosis, non-compensation HBV cirrhosis, and hepatocellular carcinoma | 940 | Total household health expenditure exceeds 40 percent of household capacity to pay | 6 |
| Li, Y2012(112) | China | Cross-sectional | Fourth National Health Service Survey (NHSS) 2008 | 55556 | Household out-of-pocket medical expenses exceed 40% of household non-survival expenses | 5 |
| Li, X2013(113) | Shanghai, Changzhou, Weifang, China | Cross-sectional | 2008 Shanghai, Changzhou, Weifang City residents health utilization and cost survey | 11577 | Household out-of-pocket medical expenses account for more than 40% of household non-survival expenses | 6 |
| Zhang, W2015(114) | China | Cross-sectional | 2011-2012 China Health and Pensions Tracking Survey of families aged 60 and over | 2700 | Medical expenses account for 40 percent or more of households' ability to pay | 4 |
| Zhou, C2016(115) | Jiangsu Province, Hubei Province, Shaanxi Province, China | Cross-sectional | TB patients diagnosed in Yichang, Han Zhong, and Zhenjiang in 2012 | 347 | Household out-of-pocket medical expenses exceed 40% of non-food expenses | 6 |
| Zheng, A2018(116) | Liaoning Province, China | Cross-sectional | A multi-stage stratified random sample survey of residents of 6 types of cancer patients in Liaoning Province | 1344 | Household out-of-pocket medical expenses account for 40 percent of household non-survival expenses | 6 |
| Gwatidzo, S. D2017(117) | China | Cross-sectional | Global Aging and Adult Health Study 2007-2010 study data for people aged 50 and over-diagnosed with non-gestational diabetes questionnaire | 630 | Out-of-pocket medical expenses account for more than 40% of household non-food expenses | 5 |
| Yang, T2016(118) | Shandong Province, China | Cross-sectional | A stratified random sample survey of elderly families in Shandong Province | 2761 | Out-of-pocket medical expenses exceed 40% of the family's ability to pay | 5 |
| Mao, W2017(119) | Shanghai, Beijing, Fuzhou, Chongqing, China | Cross-sectional | A simple random sample of cancer patients in Shanghai, Beijing, Fuzhou, and Chongqing who were reimbursed using urban job insurance | 2408 | Household health expenditure > average size of households (per capita consumption expenditure - per capita food expenditure) | 6 |
| Wang, Q2014(120) | China | Cross-sectional | "Community Health Promotion in Rural Poor Areas of China" Hong Kong Kado program 2009 survey data of 21-90-year-old with hypertension and its complications | 947 | Out-of-pocket costs exceed 40% of household affordability | 6 |
| Sun, X2016(121) | China | Cross-sectional | A simple random sample of cancer patients in Shanghai, Beijing, Fuzhou, and Chongqing who were reimbursed using urban job insurance | 5511 | Oral health expenditure accounts for 20% of the annual household income | 5 |
| CMS2016(122) | Minority areas in western China, Qinghai, Xinjiang, Tibet | Case-control | Data from the 2014 final survey on the integration of rural health services in ethnic minority areas in the west | 3066 | Household residents out-of-pocket health expenditure as a proportion of household consumption more than 40% | 6 |
| MY2015(122) | Chenyi County, Xianyang City, Shaanxi Province, China | Case-control | In 2013, the status survey was conducted on the critical illnesses in The County and the patients with critical diseases provided by the New Agricultural Joint Economic Cooperation Center in the county | 314 | Household out-of-pocket health expenses account for more than 40% of household consumption expenditure | 4 |
| JRF2014(124) | Sangzhi County, Mayo County, and Blue Mountain County, Hunan Province, China | Case-control | Random group sampling and systematic sampling methods were used to extract families from Sangzhi County, Mayo County, and Blue Mountain County in Hunan Province | 400 | The ratio of household health expenditure to household consumption expenditure exceeds 40% | 6 |
| YJE2013(125) | Mei County, Baoji City, Shaanxi Province, China | Case-control | A sample survey of Mei County, Shaanxi Province, in 2011 | 1747 | Household out-of-pocket payments account for more than 40% of household non-food expenses | 4 |
| CRY2012(126) | Tengzhou City, Shandong Province, China | Case-control | A stratified random sample survey in Tengzhou City, Shandong Province | 179 | Health expenditure accounts for 40% of household non-food expenditure | 5 |
| YRL2012(127) | Mei County, Shaanxi Province, China | Case-control | Shaanxi Province eyebrows  County household survey | 1468 | Households pay more than 40% of non-food expenses in out-of-pocket | 4 |
| QDF2016(128) | Inner Mongolia, Qinghai, China | Case-control | Survey of patients with hypertension in the pilot area | 1731 | Residents' out-of-pocket health expenditure of medical expenses accounted for more than 40% of household consumption expenditure | 6 |
| FWJ2014(129) | Hangzhou, China | Case-control | Household survey of elderly people over 60 years of age in Hangzhou | 530 | More than 40 percent of household non-food expenditures for older persons | 6 |
| JYH2017(130) | Heilongjiang Province, China | Case-control | Data from Heilongjiang Province, the fifth national health service survey, 2013 | 6601 | More than 40 percent of household non-food expenditures for older persons | 4 |
| LC2016(131) | Hubei Province, China | Case- control | The household entry survey and the new agricultural joint system records in B City, Guizhou Province | 872 | Household out-of-pocket health care costs account for more than 40% of household non-food consumer spending | 5 |
| LD2005(132) | Shanxi Province, China | Case-control | "Rural Family Health Inquiry Into Household Baseline Survey Data" | 570 | Household out-of-pocket payments account for 40% of household non-food expenses | 5 |
| SJ2017(133) | Hubei Province, China | Case-control | A random sample survey of rural areas in M City, Hubei Province | 536 | Households pay 40 percent of household income for out-of-pocket medical care and 40 percent for non-survival expenses | 6 |
| SXJ2008(134) | Xining, Yinchuan, China | Case-control | Data from the 2006 UHPP Project Xining and Yinchuan City Household Survey | 1567 | Households' monthly health expenditure accounts for more than 40% of monthly consumption expenditure (excluding food expenditure) | 6 |
| XWJ2018(135) | China | Case-control | China Health and Pensions Tracking Survey 2015 | 9706 | Medical expenditure equals or exceeds 40% of disposable income after meeting basic survival expenses | 7 |
| HG2010(136) | Jiangxi Province, China | Case-control | In 2007, a questionnaire survey of low-security farmers who lived in Yuyuan County for more than half a year | 261 | Households pay 40 percent of household income for out-of-pocket medical care and 40 percent for non-survival expenses | 5 |
| FH2004(137) | China | Case-control | China Statistical Yearbook, Archives | 9700 | Total household health expenditure accounts for more than 40% of household affordability | 6 |
| HX2010(138) | Jiangxi Province, China | Case-control | A sample survey of Shushui, Luxi, and Yuyuan counties | 15458 | Household health spending exceeds 40% of household consumption expenditure | 6 |
| SD2016(139) | Xiangtan City, Hunan Province, China | Case- control | "Urban low-income families economic and health survey" task force in 2013 Xiangtan City household survey | 1165 | Household health spending exceeds 40% of household consumption expenditure | 6 |
| ZYL2008(140) | Shanghai, Nanning, | Case-control | Information on inpatients at Sanjia Hospital in Shanghai and Nanning City in 2007 | 928 | Medical expenses paid in out-of-pocket account for 40% of the family's annual income | 4 |
| ZWG2006(141) | Dongying City, China | Case-control | Investigation on the health status and factors of rural residents in Dongying City | 579 | More than 40% of household consumption is spent on health care in out-of-pocket | 6 |
| ZYX2004(142) | Heilongjiang Province, Gansu Province, China | Case-control | Survey of households in rural areas of Gansu and Heilongjiang provinces in 2002 | 3890 | Household out-of-pocket accounts for more than 40% of household expenses | 5 |
| YXL2013(143) | A province of China | Case-control | S Province 2008 Household Health Services Survey | 3958 | Out-of-pocket hygiene expenditure accounts for more than 40% of household non-food expenditure | 5 |
| YY2014(144) | Dali Bai Autonomous Prefecture, Yunnan Province, China | Case-control | Residents of Dali City, Midu County, and Lushan County, Yunnan Province, conducted on-site investigations | 3697 | Household health expenditure accounts for more than 40 percent of household affordability | 6 |
| HHY2018(145) | Dali Bai Autonomous Prefecture, Yunnan Province, China | Case-control | A stratified random sample survey was conducted on patients hospitalized with late blood assistance in Jiangsu Province from January 2012 to December 2014 | 2626 | Personal out-of-pocket medical expenses account for 40% of the family's annual net income per capita | 7 |
| QCG2015(146) | Gansu Province, China | Case-control | Data from Gansu Province, the fifth National Health Service Survey, 2013 | 7500 | Out-of-pocket payments for medical expenses account for more than 29% of household non-food expenses | 6 |
| Chen J2018(147) | Zhejiang Province, China | Case-control | A random sample survey of rural residents in Yuyao and Fenghua, Zhejiang Province | 4900 | Household out-of-home medical expenses as a percentage of household net income in the absence of third-party compensation | 7 |
| Xu, Y2018(148) | Shaanxi Province, China | Case-control | Data on chronic families surveyed by the National Health Service in Shaanxi Province in 2008 and 2013 | 9646 | Household out-of-pocket pay more than 40 percent of household non-subsistence expenses | 6 |
| Zhang, L2010(149) | Yuexi County, Submarine County, Datong County, China | Case-control | In 2006, a random sample survey of inpatients in Yuexi County, Submarine County, and Datong County was conducted | 881 | Household out-of-pocket health care costs account for more than 10% of total annual household expenditure | 7 |
| Zhen, X2018(150) | Zhejiang, Qinghai, China | Case-control | Qinghai Family Survey 2016 | 1598 | Out-of-pocket medical expenses exceed 40% of household disposable income | 5 |
| Liu, X2016(151) | Pingyin, Liangshan, Jun'an, Shandong, China | Case- control | In June 2011, a random sample survey of patients with hypertension and diabetes in Shandong Province was conducted | 1635 | Residential medical expenses exceed 40% of household non-food expenses | 8 |
| Wang, J2014(152) | Hubei, Chongqing, Zhejiang, China | Case-control | A sample survey of rural residents in Hubei, Chongqing, and Zhejiang | 1525 | Family out-of-pocket medical expenses exceed 40% of total household income | 6 |
| Sun, J2015(153) | Inner Mongolia, China | Case-control | Family questionnaires with members of cardiovascular disease in rural areas of Inner Mongolia | 949 | Household out-of-pocket medical expenses exceed 40% of household non-self-searching expenses | 7 |
| YHY2012(154) | 25 provinces and autonomous regions of China | Cohort | China Family Dynamic Tracking Survey | 47082 | Household out-of-pocket health expenditure exceeds 40% of household income or expenditure for a certain period | 5 |
| CJH2006(155) | Province, Ningxia Province, China | Cohort | Baseline on-site research and year-end on-site research data for the project "Building a Fair and Sustainable Health Security System in China and Vietnam." | 9415 | Household out-of-pocket health expenditure exceeds 40% of household income or expenditure for a certain period | 5 |
| Gu2009(156) | City, Jiangsu Province, China | Cohort | A random sample survey of a hierarchical group in Xuzhou City | 3220 | Household out-of-pocket medical expenses account for more than 40% of household non-survival expenses | 6 |
| Jing2006(157) | Province, Ningxia Hui Autonomous Region, China | Cohort | Household surveys of chronically ill patients were conducted in Shandong and Ningxia in 2006 and 2008 | 4685 | Health expenditure accounts for 40% of household non-food expenditure | 5 |
| Li2011(158) | China | Cohort | CHARLS surveyed data on patients with chronic and cardiovascular diseases in 2011 and 2013 | 5624 | Annual household health expenditure accounts for 40% and more of household non-food expenditure | 5 |


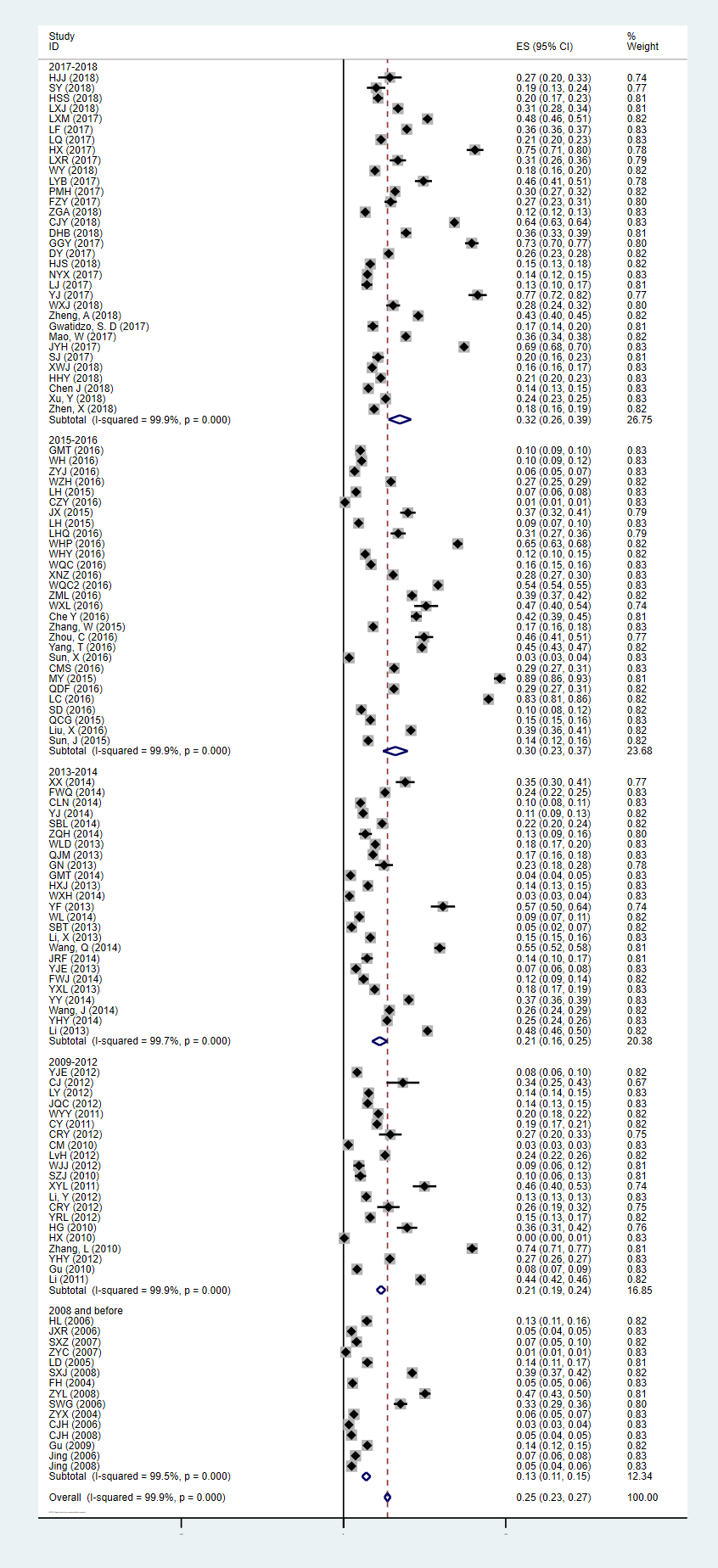


Appendix Figure 1. Secular trend of catastrophic health expenditure of all included studies


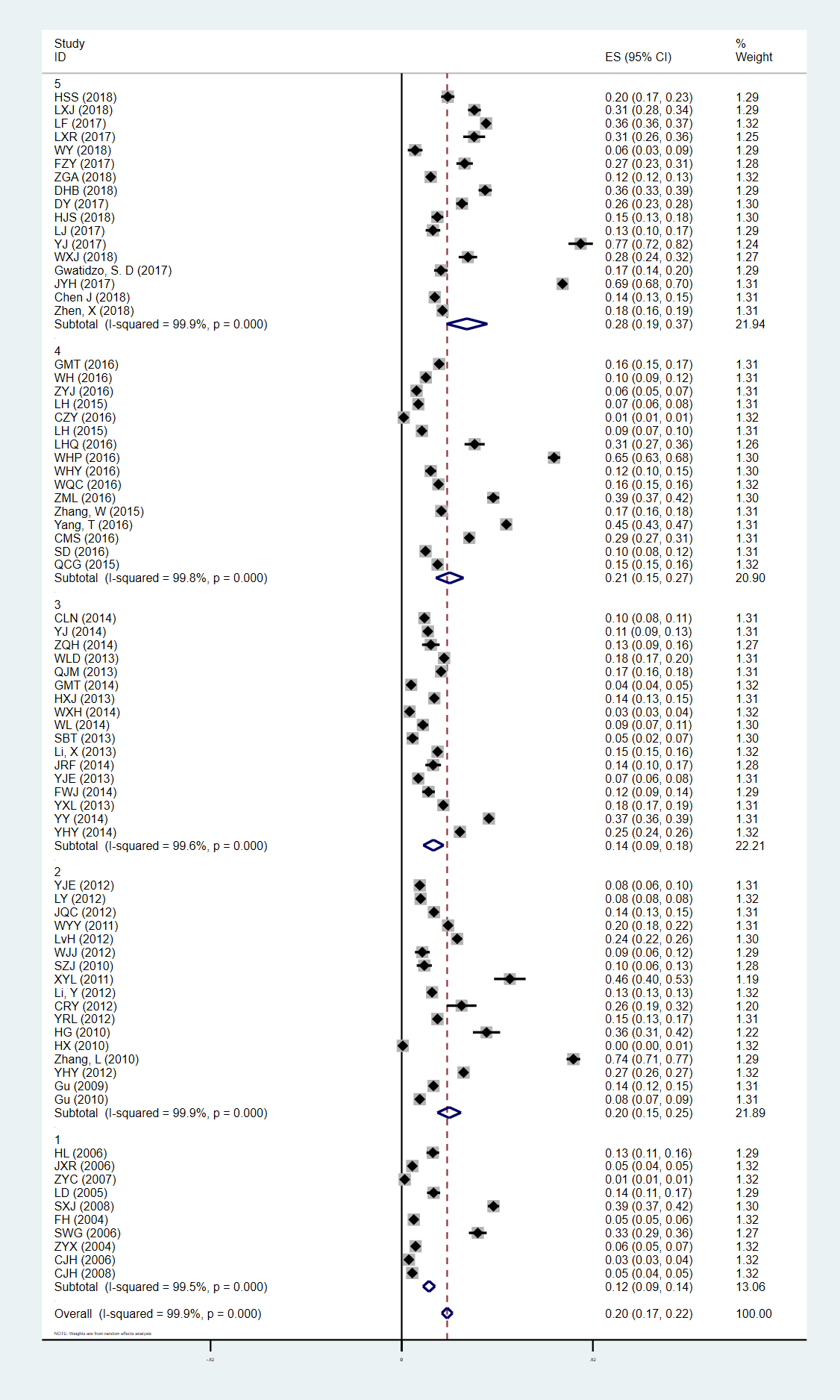


Appendix Figure 2. Secular trend of catastrophic health expenditure by the general population


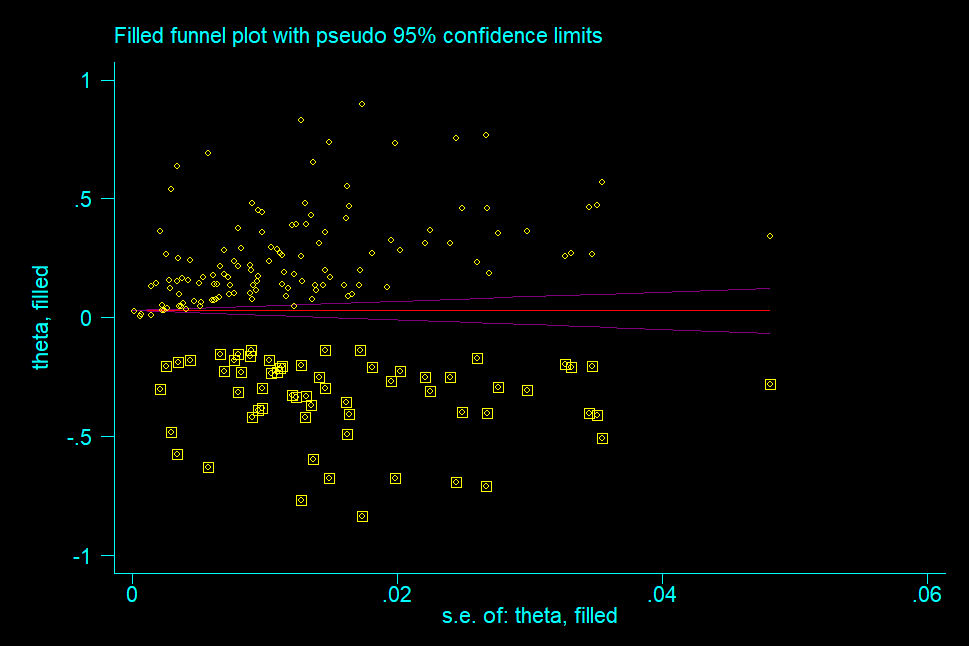


Appendix Figure 3. Trim and fill analysis funnel chart


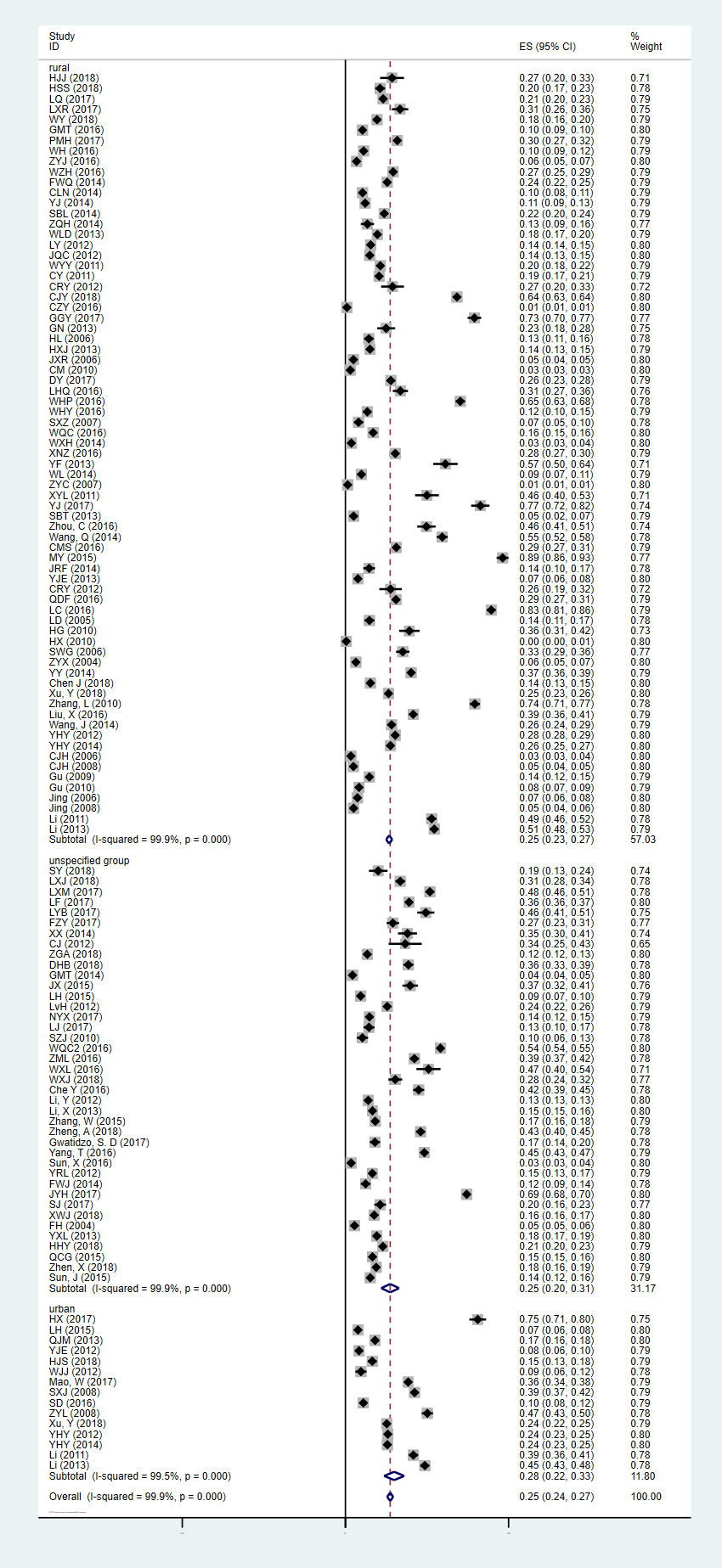


Appendix Figure 4. Rate of catastrophic health expenditure by Urban-rural differences


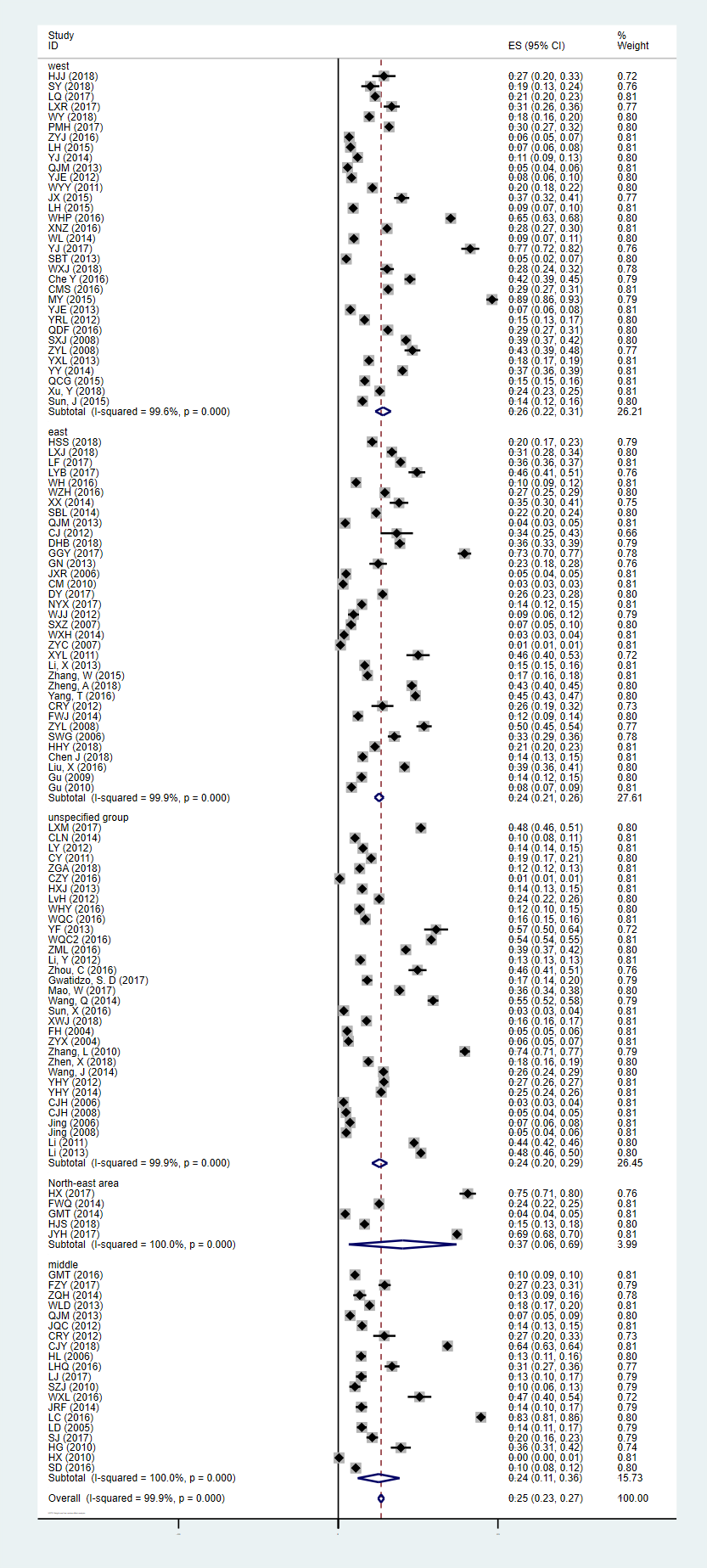


Appendix Figure 5. Rate of catastrophic health expenditure by the level of socio-economic status

Abbreviations: The eastern region included: Beijing, Tianjin, Hebei Provinces, Shandong Provinces, Jiangsu Provinces, Shanghai Provinces, Zhejiang Provinces, Fujian Provinces, Guangdong Provinces, and Hainan Provinces;

The Central region includes: Shanxi Provinces, Henan Provinces, Hubei Provinces, Hunan Provinces, Jiangxi Province, Anhui Province;

The western regions included: Chongqing City, Sichuan Province, Guangxi Zhuang Autonomous Region, Guizhou Province, Yunnan Province, Shaanxi Province, Gansu Province, Inner Mongolia Autonomous Region, Ningxia Hui Autonomous Region, Xinjiang Uygur Autonomous Region, Qinghai Province, Tibet Autonomous Region;

The northeast region included: Heilongjiang Province, Jilin Province, and Liaoning Province.


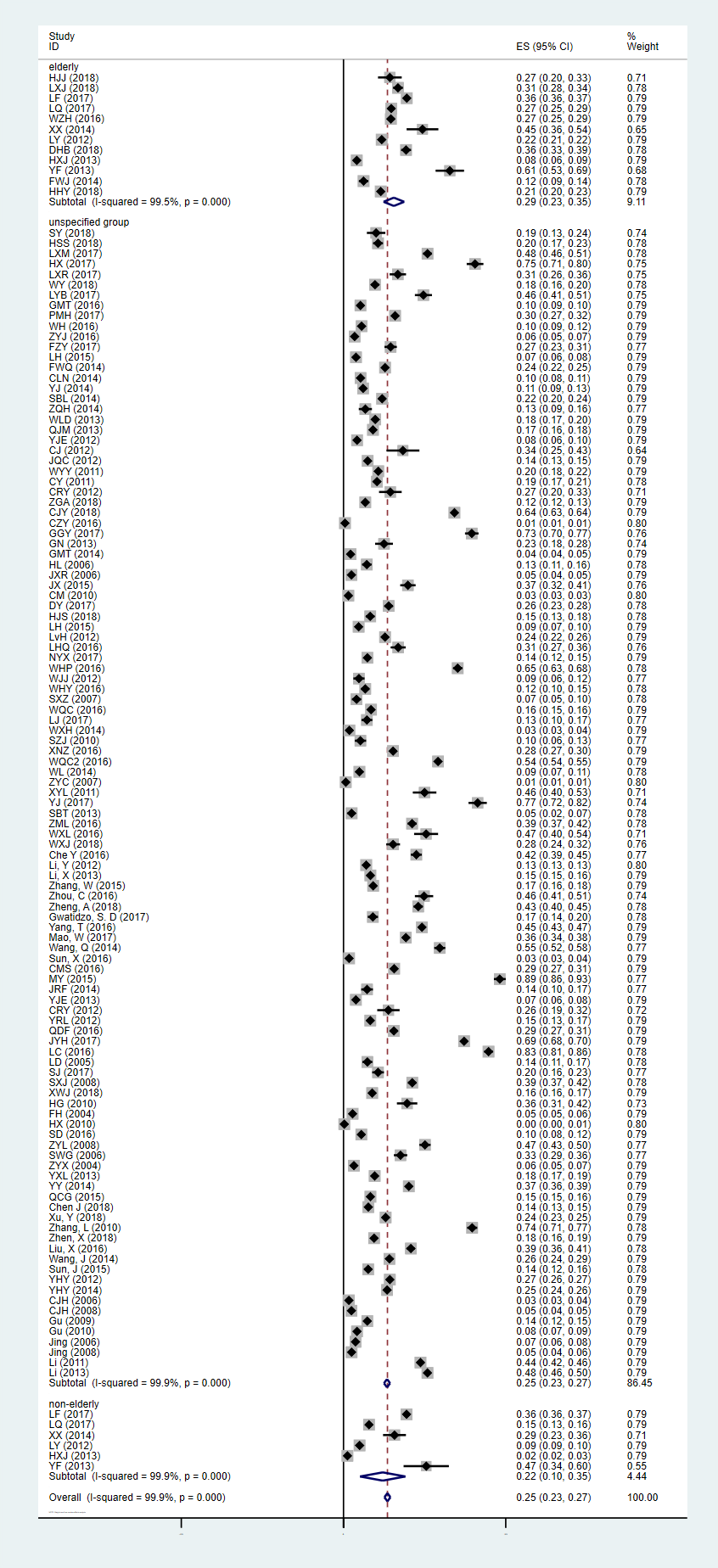


Appendix Figure 7. Rate of catastrophic health expenditure by age


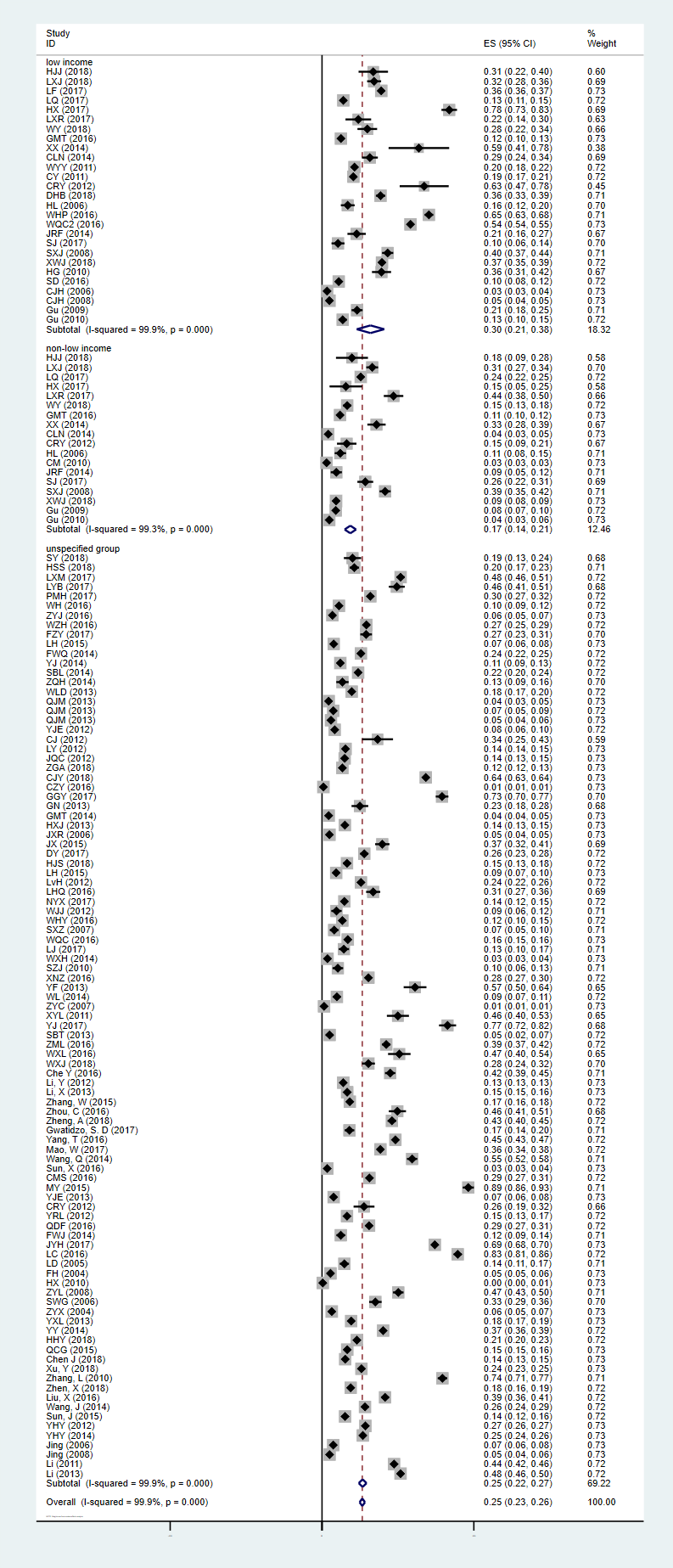


Appendix Figure 8. Rate of catastrophic health expenditure by income level


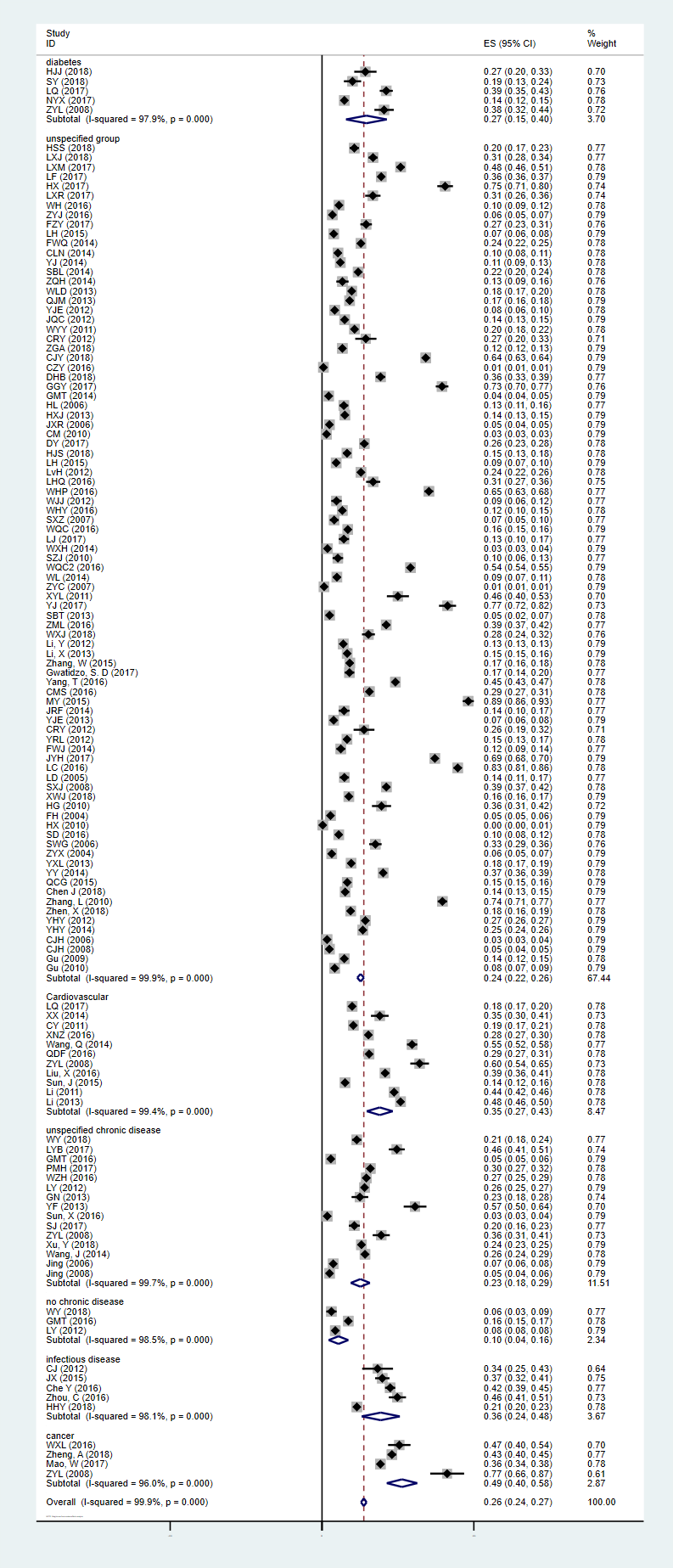


Appendix Figure 9. Rate of catastrophic health expenditure by diseases


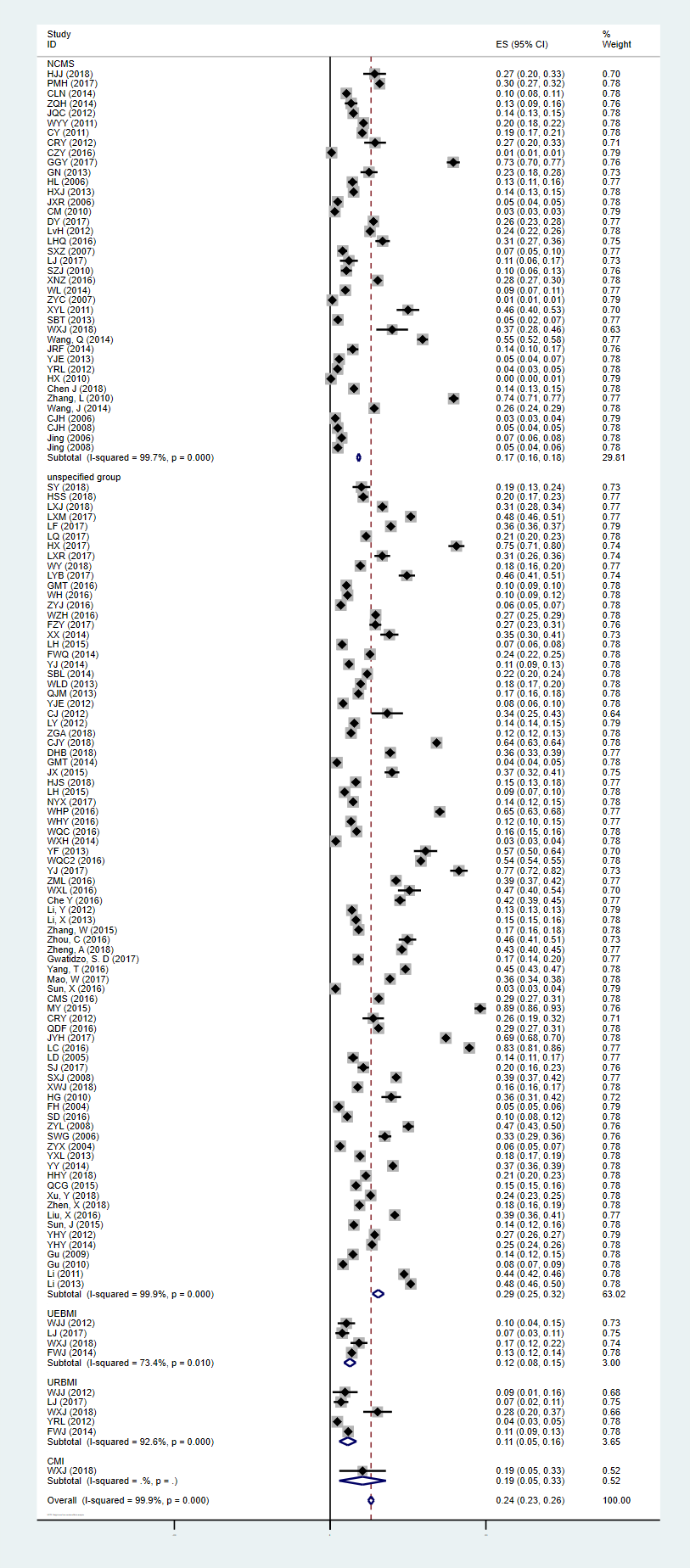


Appendix Figure 10. Rate of catastrophic health expenditure by medical insurance


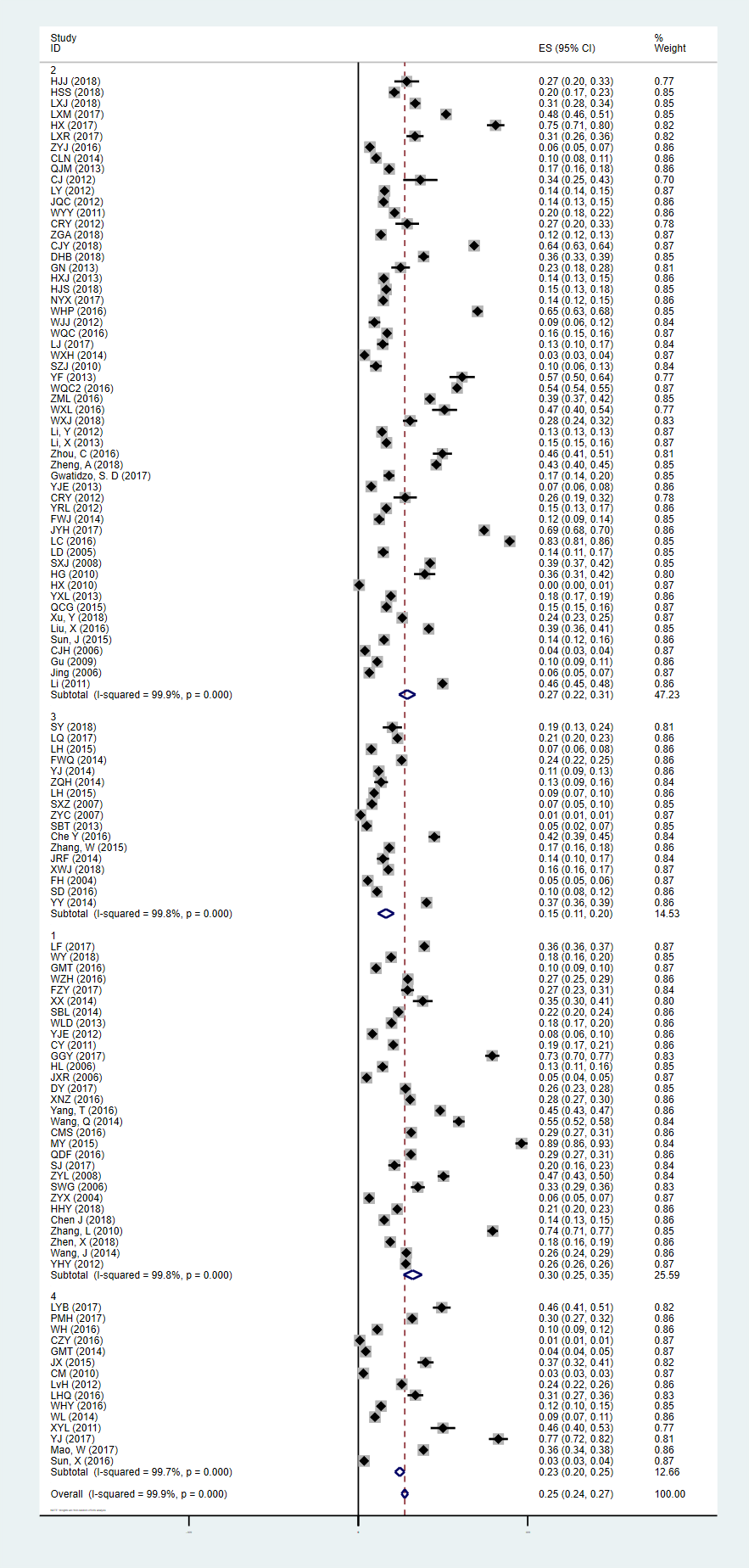


Appendix Figure 11. Rate of catastrophic health expenditure by its definition
